# An indirect comparison of efficacy including histologic assessment and safety in biologic therapy in ulcerative colitis: Systemic review and network meta-analysis

**DOI:** 10.1371/journal.pone.0293655

**Published:** 2023-11-02

**Authors:** Kyungsun Chae, Yeon Sook Seo, Yun Mi Yu, Min Jung Chang, Junjeong Choi

**Affiliations:** 1 Graduate Program of Industrial Pharmaceutical Sciences, Yeonsu-gu, Incheon, South Korea; 2 Department of Pharmacy and Yonsei Institute of Pharmaceutical Sciences, College of Pharmacy, Yonsei University, Yeonsu-gu, Incheon, South Korea; University of Glasgow School of Medicine: University of Glasgow School of Medicine Dentistry and Nursing, UNITED KINGDOM

## Abstract

**Backgrounds and aims:**

There are currently no studies comparing histologic remission of FDA-approved biologics for moderate to severe ulcerative colitis (UC), except for one head-to-head VARSITY trial. The current study employs a network meta-analysis to compare the efficacy, including histologic remission and safety of biologic agents for UC.

**Methods:**

Using four electronic databases, including Pubmed, EMBASE, The Cochrane Library, and ClinicalTrials.gov, a search was conducted of all literature published until September 2022. Included were studies of randomized controlled trials with adult patients with moderate to severe UC using biologics approved by the FDA. An odd ratio with a 95 percent credible interval and ranking information was calculated for each endpoint.

**Results:**

The results of the network meta-analysis did not reveal statistically significant differences among biological agents. However, the ranking information for each biological agent exhibited the following patterns. Vedolizumab was ranked first for overall efficacy endpoints in the maintenance phase, including histologic remission. Except for histologic remission, Ustekinumab was identified as the top-ranked drug for induction phase efficacy endpoints other than histologic remission. Adalimumab was identified as the top-ranked drug for maintenance phase corticosteroid-free remission. Vedolizumab was identified as the top-ranked drug in the induction phase for Treatment Emergent Adverse Events (TEAE). Adalimumab was identified as the top-ranked drug in the induction phase for infection. For TEAE and infection in the maintenance phase and Treatment Emergent Severe Adverse Events (TESAE) in both the induction and maintenance phases, Ustekinumab was determined to be the top-ranked medication.

**Conclusions:**

Including histologic remission, for the overall efficacy endpoints in the maintenance phase, VDZ was identified as the first rank drug, but there was no statistically significant difference between biologics. Therefore, the generalization of the results of this study is bounded due to the intrinsic limitations of the study provided.

## Introduction

### Ulcerative colitis

Ulcerative colitis (UC) is a chronic, inflammatory bowel disease characterized by alternating relapse and remission periods [1]. The most prevalent UC symptoms are diarrhea and blood in the stool. Patients may also experience varying degrees of abdominal pain, mucus discharge, urgency, and/or extraintestinal symptoms, depending on the severity and location of the disease [2,3]. In addition to being associated with an increased risk of colorectal cancer, UC can lead to colectomy, which provides symptomatic relief but no cure and is associated with complications in up to one-third of patients [4,5]. As the characteristics and prognosis of UC can have an impact on the long-term quality of life and work productivity, it is of the utmost importance to establish an appropriate treatment objective and strategy. Especially in the context of moderate to severe UC where biologics are employed, the selection and strategy of treatment become crucial. The use of biologic agents can significantly impact the disease course and management, potentially altering the trajectory of relapses and remissions.

### Treatment target of UC

#### Clinical target

Clinical targets are the resolution of rectal bleeding and the normalization of bowel [6]. They are non-invasive, do not have any additional costs, so it has been used to estimate disease activity in UC management for a long time. However, UC management that only focuses on clinical targets is not sufficient as symptomatic control may leave less active or smoldering disease lingering, increasing the risk of relapse [6,7].

#### Endoscopic target

Endoscopic target is absence of ulceration based on the results of endoscopy [6]. As an objective evidence of inflammation, endoscopic target is associated with in lower incidences of relapse, hospitalization and colectomy, as well as lower rates of dysplasia and colorectal cancer than clinical targets [8]. However, it does not address histologic inflammation. A meta-analysis of 1573 UC patients showed that the endoscopic target was worse at predicting clinical outcomes than the histologic target [9].

#### Histologic target

However, recently, it has become increasingly popular and been recognized as an important prognostic factor. Histologic target is the normalization of active histological inflammation. There is still debate over whether the histologic target should be considered as an additional treatment target, as its clinical utility is still limited in its clinical utility and received a low rating as an independent treatment target by the Delphi group [10–12]. In spite of this, histologic target has emerged as an important prognostic factor and potential treatment target in patients with UC recently [13,14]. Multiple studies support the incorporation of histologic remission into treatment targets of both clinical trials and practice of UC [15,16]. In addition, the U.S. Food and Drug Administration (FDA) recommended histologic response/remission as exploratory endpoints in clinical trials for UC treatments under development through the guideline in April 2022 [17].

#### Aim of this study

Currently, the FDA-approved biologics for moderate to severe UC are Adalimumab (ADA), Golimumab (GOL), Infliximab (IFX), Ustekinumab (UST), and Vedolizumab (VDZ). Among these biologics, only UST which is latest approved, set histologic remission as an efficacy endpoint in its pivotal clinical trial. For some new drugs, histological remission has now become an endpoint for pivotal clinical trials in UC.

However, despite the growing importance of histologic remission as a treatment target for UC, there are no direct comparisons of histologic remission in the FDA-approved biologics for UC, with the exception of one head-to-head VARSITY trial (adalimumab [ADA] vs vedolizumab [VDZ]) [18]. Additionally, indirect comparisons of histologic remission have not been conducted systematically. There are no reference data for selecting biologics when histologic remission is the treatment goal.

The purpose of the study is to compare the efficacy and safety of biologic therapy for moderate to severe UC in terms of efficacy, including histologic remission, in order to provide reliable evidence that can be considered when selecting biologics with a therapeutic target for histologic remission.

## Material and methods

### Search strategy and study selection

This study was conducted according to the Preferred Reporting Items for Systematic Review and Network Meta-analysis (PRISMA NMA) checklist (S1 File), which is an extension of traditional pairwise meta-analysis [19]. For efficient evidence collection, the research question was set based on the PICO-SD (P: Population, I: Intervention, C: Comparator, O: Outcome, SD: Study Design) framework (S2 File) [20].

The primary question was "Is there a difference in efficacy including histologic assessment, and safety between FDA-approved biologic therapies for moderate to severe UC in adult patients with moderate-to-severe UC?". The participants were adults with moderate to severe UC. The intervention and comparison will be FDA-approved biologics for moderate to severe UC (ADA, GOL, IFX, UST, and VDZ) and placebo, respectively. The design of the study was restricted to human RCTs. In the case of the protocol, it was developed during the paper submission process after the completion of this study. The study protocol which includes details of this study is publicly available on protocols.io [21].

Using 4 electronic databases (Pubmed, EMBASE, and The Cochrane Library, as well as the ClinicalTrials.gov site that provides clinical information) considered major database in medical science topics, and have a larger platform than other databases, a search was conducted on literature published up until September 2022. The full search strategies for each database are included in S3 File.

On the basis of the collected literature, two researchers (KSC, YSS) independently selected the literature. In the event of a disagreement during the literature review selection procedure, the final decision was reached through discussion. Full-text review was used to select the literatures for analysis based on the following criteria: a study of adult patients with moderately to severely UC, a study using biologics in the same regimen as the FDA-approved regimen, a study that includes the efficacy and/or safety results of the induction and maintenance phase after administration of biologics, and a randomized controlled trial. As it was difficult to accurately determine whether a study was an RCT or non-RCT based solely on the abstract and excluding hundreds of papers in the initial stages was challenging due to the possibility that even partial information relevant to our study might be present, 868 papers full-text based on the PRISMA flowchart was reviewed. As a result, RCTs, review studies, observational studies, case studies, academic abstracts, correspondence, and ongoing studies without reported results that utilized biologics were excluded. List of excluded studies is included in S4 File.

### Outcome assessment

Histologic remission and other efficacy endpoints such as clinical remission, corticosteroid-free remission, and endoscopic improvement were the primary outcome measures of interest. Treatment emergent adverse event (TEAE), treatment emergent serious adverse event (TESAE), and infection were of interest as safety outcome measures (TESAE).

Except for histologic remission, all outcome measures have identical or nearly identical definitions. There is currently no standard definition for histologic remission, so the definitions and terms of histologic assessment established for each study are not exactly identical. Definitions of efficacy and safety outcome measures are presented in S5 File [22–24].

### Data extraction

Two independent reviewers (KSC, JC) extracted all data from ten selected clinical trials into a separate Microsoft Excel spreadsheet. Extracted were the trial’s identifier, eligibility criteria, phase with duration, posology of the treatment group, sample size, and baseline patient characteristics. When available, data for pre-specified outcome measures were extracted at the conclusion of the induction and maintenance phases for each study.

### Quality assessment and risk of bias

Risk of Bias (RoB) 2, a tool developed by the Cochrane group specifically for randomized controlled trials, was utilized. Following items were assessed: 1) bias resulting from the randomization procedure, 2) bias resulting from deviations from intended interventions, 3) bias resulting from missing outcome data, 4) bias in measurement of the outcome, and 5) bias in selection of the reported result. There were three levels of risk of bias assessment: Low risk of bias, Some concerns, and High risk of bias [25].

### Data synthesis and statistical analysis

To compare the effects of each biologics at the same time, network meta-analysis (NMA) based on the Bayesian framework by integrating all available study results was conducted [26]. All NMA were analyzed using R software version 4.2.0 (R foundation for Statistical Computing, Vienna, Austria) and GEMTC package was used.

The network was set up based on the study results available for each endpoint, and a random-effect model was applied in consideration of the heterogeneity in endpoints and study design of each study. Odd ratio (OR) and 95% credible interval (CrI) of the results were derived, since the efficacy and safety endpoints are all binary variables (proportion [%]). In addition, ranking information including that one treatment is ranked higher than another treatment for each endpoint were also derived through probability.

### Sensitivity analysis

In order to evaluate the effect of race on the 2 clinical trials performed only on Japanese subjects, sensitivity analysis was conducted with the same simulation settings as the main analysis using R software version 4.2.0, GEMTC package.

## Results

**Fig 1:**
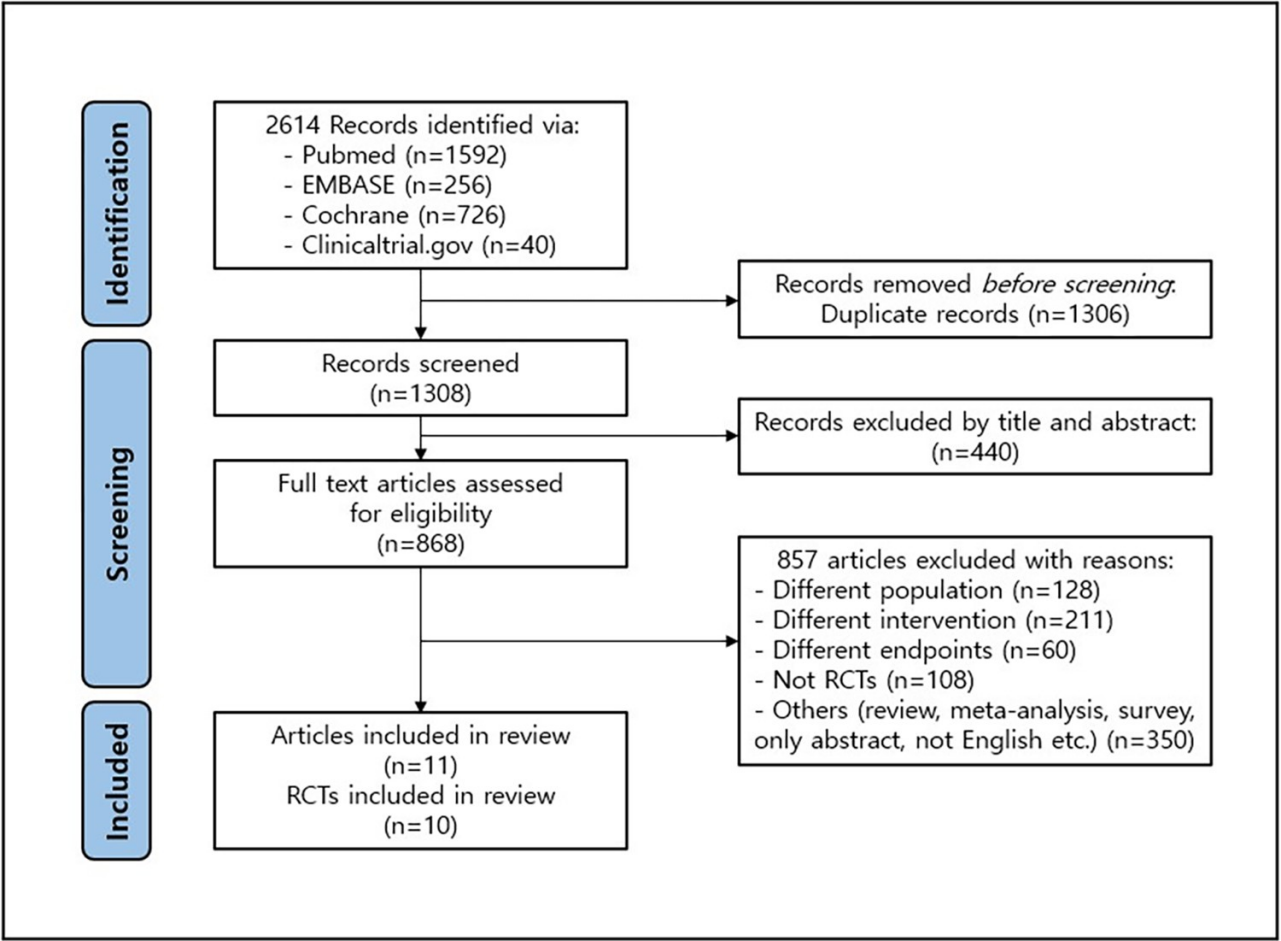
PRISMA flow chart of the study.

A total of 10 RCTs of 11 literatures were selected for this study [18,27–36]. The flow chart of the literature review is as shown in Fig 1. Regarding the intervention drugs, IFX and GOL were excluded from this analysis as there was no study that could confirm the histologic assessment results, even though there were other efficacy results of them.

All enrolled patients were aged ≥ 18 years and diagnosed with moderately to severely UC, defined as a total of Mayo score 6 to 12 points. Unlike others, Suzuki et al. 2014, HIBISCUS 1 and 2 trials [28,29] were conducted only on biologic naïve patients, and other clinical trials were conducted on patients who had failed TNF⍺-I or biologics. In addition, Suzuki et al. 2014 and Motoya et al. 2019 trials [30,35] were conducted on Japanese only. For the treatment groups, PBO group was set for all trials except for the VARSITY trial [18], which is the only head-to-head trial (ADA vs. VDZ). In Motoya et al. 2019 and VISIBLE1 trials [30,32], the number of patients per treatment group was small, approximately 50 patients, but other clinical trials included approximately 100 or more patients per treatment group.

The results of histologic remission, key endpoint of this study, were available in 5 clinical trials, UNIFI, VARSITY, VISIBLE1, HIBISCUS 1 and 2 trials [18,27,32,36], but the definitions were not exactly identical in each clinical trial, as mentioned above. About the study period, ULTRA1, HIBISCUS1 and 2 trials [33,36] were conducted only during the induction phase, and others were conducted during the induction and maintenance phases. The details of patient baseline characteristic and outcome included in this study were presented in Tables 1 and 2, respectively.

**Table 1:** Baseline characteristics of studies included in the network meta-analysis.

Study name, Identifier (Author. year)	Key Eligibility	Phase (Time point)	Posology of Treatment group	Sample size, n	Male Sex, n (%)	Mean Age (yr)	Mean Disease duration (yr)	Mean MS	Concomitant Medication	
									IMM n (%)	CS n (%)
**Ustekinumab**										
UNIFI, NCT02407236 (Sands et al. 2019 [19], Li et al. 2020 [20])	biologic naïve or have failed biologic therapy	I (W8)	PBO	319	197 (61.8)	41	8.0	8.9	89 (27.9)	157 (49.2)
			UST (IV) 6mg/kg at W4	322	195 (60.6)	42	8.2	8.9	89 (27.6)	168 (52.2)
		M (W52)	PBO	175	61 (34.9)	42	7.5	NR	49 (28.0)	95 (54.3)
			UST (SC) 90mg Q8W	176	53 (30.1)	40	8.1	NR	45 (25.6)	95 (54.0)
**Vedolizumab**										
GEMINI1, NCT00783718 (Feagan et al. 2013 [21])	biologic naïve or have failed TNF⍺-I therapy	I (W6)	PBO	149	92 (61.7)	41	7.1	8.6	44 (29.5)	84 (56.3)
			VDZ (IV) 300 mg at W0, 2	225	132 (58.7)	40	6.1	8.5	80 (35.4)	120 (53.2)
		M (W52)	PBO	126	70 (55.6)	40	NR	7.8	50 (40.0)	72 (57.0)
			VDZ (IV) 300 mg Q8W	122	70 (57.3)	41	NR	6.2	44 (36.0)	70 (57.0)
NCT02039505 (Motoya et al. 2019 [22])	biologic naïve or have failed TNF⍺-I therapy, Japanese	I (W10)	PBO	82	66 (67.1)	44	8.6	8.1	43 (52.5)	25 (30.5)
			VDZ (IV) 300 mg at W0, 2, and W6	164	99 (60.4)	42	7.2	8.3	80 (48.8)	52 (31.7)
		M (W60)	PBO	42	23 (54.8)	43	8.7	7.9	21 (50.0)	15 (35.7)
			VDZ (IV) 300 mg Q8W	41	21 (51.2)	43	8.6	8.1	22 (53.8)	13 (31.8)
VARSITY, NCT02497469 (Sands et al. 2019 [16], P.B et al. 2021 [23])	biologic naïve or have failed TNF⍺-I therapy	I (W14) & M (W52)	ADA (SC) 160mg at W0, 80mg at W2, 40mg Q2W	386	216 (56.0)	41	6.4	8.7	100 (25.9)	140 (36.3)
			VDZ (IV) 300 mg at W0, 2, 6 and Q8W	385	234 (60.8)	41	7.3	8.7	101 (26.2)	139 (36.1)
VISIBLE1, NCT02611830 (Sandborn et al. 2020 [24])	biologic naïve or have failed TNF⍺-I therapy	I & M (W52)	PBO	56	34 (60.7)	40	7.4	9.0*	NR	24 (42.9)
			VDZ (IV) 300 mg at W0, 2, 6 and Q8W	54	31 (57.4)	42	8.2	9.0*	NR	21 (38.9)
**Adalimumab**										
ULTRA1, NCT00385736 (Renisch et al. 2011 [25])	biologic naïve	I (W8)	PBO	130	82 (63.1)	37	5.4	8.7	52 (39.9)	88 (67.6)
			ADA (SC) 160mg at W0, 80mg at W2, 40mg at W4, and W6	130	83 (63.8)	37	6.1	8.8	51 (39.2)	71 (54.6)
ULTRA2, NCT00408629 (Sandborn et al. 2013 [26])	biologic naïve or have failed TNF⍺-I therapy	I (W8), M (W52)	PBO	246	152 (61.8)	41	8.5	8.9	125 (50.8)	185 (75.2)
			ADA (SC) 160mg at W0, 80mg at W2, 40mg Q2W	248	142 (57.3)	40	8.1	8.9	143 (57.7)	200 (80.7)
NCT00853099 (Suzuki et al. 2014 [27])	biologic naïve, Japanese	I (W8)	PBO	96	70 (72.9)	41	7.8	8.5	NR	NR
			ADA (SC) 160mg at W0, and 80mg at W2	90	61 (67.8)	43	8.3	8.6	NR	NR
		M (W52)	PBO	96	70 (72.9)	41	7.8	8.5	52** (54.2)	58** (60.4)
			ADA (SC) 40mg Q2W	177	111 (62.7)	43	8.0	8.6	81** (45.6)	112** (63.3)
HIBISCUS1, NCT02163759 (Rubin et al. 2022 [28])	biologic naïve	I (W10)	PBO	72	39 (54.2)	38	NR	NR	15 (20.8)	25 (34.7)
			ADA (SC) 160mg at W0, 80mg at W2, 40mg at W4, 6, and W8	142	82 (57.7)	42	NR	NR	30 (21.1)	46 (32.4)
HIBISCUS2, NCT02171429 (Rubin et al. 2022 [28])	biologic naïve	I (W10)	PBO	72	38 (52.8)	40	NR	NR	14 (19.4)	23 (31.9)
			ADA (SC) 160mg at W0, 80mg at W2, 40mg at W4, 6, and W8	143	81 (56.6)	40	NR	NR	28 (19.6)	42 (29.4)

Abbreviations: ADA; adalimumab, CS; corticosteroid; n; the number of patients, I; induction, IMM; immunomodulatory, IV; intravenous, M; maintenance, MS; Mayo score, NR; not reported, PBO; placebo, Q2W; every 2 weeks, Q8W; every 8 weeks, SC; subcutaneous, TNF⍺-I; tumor necrosis factor alpha inhibitor, UST; ustekinumab, VDZ; vedolizumab, W; week, yr; years.

*median results were reported due to data limitation.

** Results during both induction and maintenance period were reported due to data limitation.

**Table 2:** Efficacy and safety results of studies included in the network meta-analysis.

Study name, Identifier (Author. year)	Time point	Study drug	Efficacy result				Safety result*		
			Clinical remission, n/N (%)	Corticosteroid-free remission, n/N (%)	Endoscopic improvement, n/N (%)	Histologic remission, n/N (%)	TEAE, n/N (%)	TESAE, n/N (%)	Infection, n/N (%)
**Ustekinumab**									
UNIFI, NCT02407236 (Sands et al. 2019 [19], Li et al. 2020 [20])	W8	PBO	17/319 (5.3)	N/A	44/319 (13.8)	65/297 (21.9)	153/319 (48.0)	22/319 (6.9)	49/319 (15.4)
		UST	50/322 (15.5)	N/A	87/322 (27.0)	105/295 (35.6)	162/320 (50.6)	11/320 (3.4)	51/321 (15.9)
	W52	PBO	42/175 (24.0)	41/175 (23.4)	50/175 (28.6)	55/167 (32.9)	138/175 (78.9)	17/175 (9.7)	81/175 (46.3)
		UST	77/176 (43.8)	74/176 (42.0)	90/176 (51.1)	99/167 (59.3)	136/176 (77.3)	15/176 (8.5)	86/176 (48.9)
**Vedolizumab**									
GEMINI1, NCT00783718 (Feagan et al. 2013 [21])	W6	PBO	8/149 (5.4)	N/A	37/149 (24.8)	N/A	NR	NR	NR
		VDZ	38/225 (16.9)	N/A	92/225 (40.9)	N/A	NR	NR	NR
	W52	PBO	20/126 (15.9)	10/72 (13.9)	25/126 (19.8)	N/A	67/149 (45.0)	17/149 (11.4)	29/149 (19.5)
		VDZ	51/122 (41.8)	22/70 (31.4)	63/122 (51.6)	N/A	69/126 (54.8)	20/126 (15.9)	39/126 (31.0)
NCT02039505 (Motoya et al. 2019 [22])	W10	PBO	10/82 (12.2)	N/A	25/82 (30.5)	N/A	43/82 (52.4)	4/82 (4.9)	10/82 (12.2)
		VDZ	30/164 (18.3)	N/A	60/164 (36.6)	N/A	82/164 (50.0)	10/164 (6.1)	24/164 (14.6)
	W60	PBO	13/42 (31.0)	3/15 (20.0)	14/42 (33.3)	N/A	33/42 (78.6)	3/42 (7.1)	11/42 (26.2)
		VDZ	23/41 (56.1)	6/13 (46.2)	26/41 (63.4)	N/A	36/41 (87.8)	4/41 (9.8)	19/41 (46.3)
VARSITY, NCT02497469 (Sands et al. 2019 [16], P-B et al. 2021 [23])	W14	ADA	82/386 (21.2)	N/A	N/A	118/386 (30.6)	NR	NR	NR
		VDZ	102/383 (26.6)	N/A	N/A	173/383 (45.2)	NR	NR	NR
	W52	ADA	87/386 (22.5)	26/119 (21.8)	107/386 (27.7)	119/386 (30.8)	267/386 (69.2)	53/386 (13.7)	55/386 (14.2)
		VDZ	120/383 (31.3)	14/111 (12.6)	152/383 (39.7)	175/383 (45.7)	240/383 (62.7)	42/383 (11.0)	54/383 (14.1)
VISIBLE1, NCT02611830 (Sandborn et al. 2020 [24])	W52	PBO	8/56 (14.3)	2/24 (8.3)	12/56 (21.4)	4/56 (7.1)	43/56 (76.8)	6/56 (10.7)	14/56 (25.0)
		VDZ	23/54 (42.6)	6/21 (28.6)	29/54 (53.7)	6/54 (11.1)	41/54 (75.9)	7/54 (13.0)	15/54 (27.8)
**Adalimumab**									
ULTRA1, NCT00385736 (Renisch et al. 2011 [25])	W8	PBO	12/130 (9.2)	N/A	54/130 (41.5)	N/A	108/223 (48.4)	17/223 (7.6)	35/223 (15.7)
		ADA	24/130 (18.5)	N/A	61/130 (46.9)	N/A	112/223 (50.2)	9/223 (8.5)	32/223 (14.3)
ULTRA2, NCT00408629 (Sandborn et al. 2013 [26])	W8	PBO	23/246 (9.3)	N/A	78/246 (31.7)	N/A	163/246 (66.3)	21/246 (8.5)	51/246 (20.7)
		ADA	41/248 (16.5)	N/A	102/248 (41.1)	N/A	144/247 (58.3)	15/247 (6.1)	50/247 (20.2)
	W52	PBO	21/246 (8.5)	8/51 (15.7)	38/246 (15.4)	N/A	142/260 (54.6)	32/260 (12.3)	48/260 (18.5)
		ADA	38/123 (30.9)	19/90 (21.1)	53/123 (43.1)	N/A	139/257 (54.1)	31/257 (12.6)	60/257 (23.3)
NCT00853099 (Suzuki et al. 2014 [27])	W8	PBO	11/96 (11.5)	N/A	30/96 (31.2)	N/A	45/96 (46.9)	7/96 (7.3)	15/96 (15.6)
		ADA	10/90 (11.1)	N/A	44/90 (48.9)	N/A	40/90 (44.4)	4/90 (4.4)	17/90 (18.9)
	W52	PBO	7/96 (7.3)	4/58 (6.9)	16/96 (16.7)	N/A	51/96 (53.1)	13/96 (13.5)	32/96 (33.3)
		ADA	23/177 (13.0)	17/120 (14.2)	29/177 (16.4)	N/A	107/177 (60.5)	24/177 (13.6)	70/177 (40.0)
HIBISCUS1, NCT02163759 (Rubin et al. 2022 [28])	W10	PBO	5/72 (6.9)	N/A	16/72 (22.2)	10/62 (16.1)	26/62 (41.9)	2/62 (3.2)	7/72 (9.7)
		ADA	32/142 (22.5)	N/A	47/142 (33.1)	34/142 (23.9)	61/142 (43.0)	3/142 (2.1)	17/142 (12.0)
HIBISCUS2, NCT02171429 (Rubin et al. 2022 [28])	W10	PBO	8/72 (11.1)	N/A	22/72 (30.6)	13/62 (21.0)	14/72 (19.4)	5/72 (6.9)	13/72 (18.1)
		ADA	35/143 (24.5)	N/A	61/142 (43.0)	50/114 (43.9)	23/143 (16.1)	3/143 (2.1)	18/143 (12.6)

Abbreviations: ADA; adalimumab, n; the number of patients, N/A; not applicable, NR; not reported, PBO; placebo, TEAE; treatment emergent adverse event, TESAE; treatment emergent serious adverse event, UST; ustekinumab, VDZ; vedolizumab, W; week.

*Safety results were assessed at the end of study visit or at the end of the maintenance phase.

### Efficacy: Histologic remission

In ADA, UST, and VDZ, there was no statistically significant difference in achieving histologic remission compared to PBO in both induction and maintenance phases. In comparison between biologics, there was no statistically significant difference in achieving histologic remission in both induction and maintenance phases as well. The 1^st^ rank drug for histologic remission was identified as VDZ in both induction and maintenance phases through rank probability. (Table 3, Table 1 in S6 File)

**Table 3:** Network meta-analysis results of efficacy outcome.

	**Maintenance of Histologic remission**			
**Induction of Histologic remission**	VDZ^I^^,^ ^M^	2.40 [0.50, 11.9]	1.66 [0.14, 23.5]	4.34 [0.59, 35.0]
	1.81 [0.50, 6.31]	ADA	0.692 [0.03,15.3]	2.60 [0.52, 13.2]
	2.29 [0.30, 18.2]	1.25 [0.26, 6.25]	UST	1.78 [0.14, 23.8]
	4.19 [0.82, 20.0]	2.32 [0.86, 6.12]	1.85 [0.50, 6.60]	PBO
	**Maintenance of Clinical remission**			
**Induction of Clinical** **remission**	VDZ ^M^	1.39 [0.64, 2.89]	1.69 [0.42, 5.77]	**4.21 [2.10, 7.68]**
	1.30 [0.69, 2.32]	ADA	1.23 [0.33, 4.29]	**3.08 [1.40, 6.00]**
	0.82 [0.27, 2.44]	0.63 [0.22, 1.86]	UST ^I^	2.51 [0.84, 7.86]
	**2.66 [1.48, 4.85]**	**2.05 [1.35, 3.24]**	**3.26 [1.31, 8.66]**	PBO
	**Maintenance of Corticosteroid-free remission**			
**Induction of Corticosteroid-free remission**	ADA ^M^	1.17 [0.36, 3.02]	1.17 [0.23, 6.30]	**2.77 [1.01, 7.53]**
	N/A	VDZ	1.00 [0.22, 5.65]	**2.39 [1.04, 6.25]**
	N/A	N/A	UST	2.35 [0.60, 9.30]
	N/A	N/A	N/A	PBO
	**Maintenance of Endoscopic remission**			
**Induction of Endoscopic** **remission**	VDZ ^M^	1.56 [0.29, 7.90]	1.78 [0.67, 4.78]	**4.06 [1.85, 9.00]**
	0.75 [0.34, 1.63]	UST ^I^	1.14 [0.20, 6.21]	2.60 [0.60, 11.2]
	1.09 [0.58, 1.97]	1.45 [0.71, 2.86]	ADA	2.30 [0.90, 5.58]
	**1.73 [1.04,2.89]**	**2.31 [1.22, 4.37]**	**1.60 [1.18, 2.22]**	PBO

Abbreviations: ADA; adalimumab, N/A; not applicable PBO; placebo, UST; ustekinumab, VDZ; vedolizumab.

Note: Comparison should be read from left to right. Bold numbers with highlighted background are statistically significant.

^I^: The 1^st^ rank drug of induction phase.

^M^: The 1^st^ rank drug of maintenance phase.

### Efficacy: Clinical remission

ADA, UST, and VDZ were found to be statistically significantly effective in achieving clinical remission compared to PBO, in general. However, there was no statistically significant difference in achieving clinical remission between UST (OR: 2.51 [95% CrI: 0.834, 7.86]) compared to PBO in the maintenance phase. In comparison between biologics, there was no statistically significant difference in achieving clinical remission in both induction and maintenance phases. The 1^st^ rank drug for clinical remission was identified as UST in the induction phase and VDZ in the maintenance phase through rank probability. (Table 3, Table 2 in S6 File)

### Efficacy: Corticosteroid-free remission

ADA and VDZ were found to be statistically significantly effective in achieving corticosteroid-free remission compared to PBO. However, there was no statistically significant difference in achieving corticosteroid-free remission between UST (OR: 2.35 [95% CrI:0.597, 9.30]) compared to PBO. In comparison between biologics, there was no statistically significant difference in achieving corticosteroid-free remission in the maintenance phase. The 1^st^ rank drug for clinical remission was identified as ADA through rank probability. (Table 3, Table 3 in S6 File).

### Efficacy: Endoscopic remission

ADA, UST, and VDZ were found to be statistically significantly effective in achieving endoscopic improvement compared to PBO in the induction phase. However, there was no statistically significant difference in achieving endoscopic improvement in ADA (OR: 2.30 [95% CrI:0.901, 5.58]) and UST (OR:2.60 [95% CrI:0.604, 11.2]) compared to PBO in the maintenance phase. In comparison between biologics, there was no statistically significant difference in achieving endoscopic improvement in both induction and maintenance phases. The 1^st^ rank drug for endoscopic improvement was identified as UST in the induction phase and VDZ in the maintenance phase through rank probability (Table 3, Table 4 in S6 File).

### Safety: TEAE

In ADA, UST, and VDZ, there was no statistically significant difference in TEAE incidence compared to PBO in both induction and maintenance phases. In comparison between biologics, there was no statistically significant difference in TEAE incidence in both induction and maintenance phases as well. The 1^st^ rank drug for TEAE was identified as VDZ in the induction phase and UST in the maintenance phase through rank probability (Table 4, Table 5 in S6 File).

**Table 4:** Network meta-analysis results of safety outcome.

	**Maintenance of TEAE**			
**Induction of TEAE**	UST M	0.91 [0.42, 2.00]	0.79 [0.31, 1.94]	0.72 [0.30, 1.83]
	1.11 [0.68, 1.77]	PBO	0.87 [0.51, 1.34]	0.79 [0.49, 1.24]
	1.20 [0.55, 2.62]	1.08 [0.59, 2.07]	VDZ I	0.92 [0.55, 1.60]
	1.25 [0.71, 2.09]	1.12 [0.86, 1.46]	1.04 [0.51, 2.00]	ADA
	Maintenance of TESAE			
**Induction of TESAE**	UST I, M	0.88 [0.37, 2.00]	1.24 [0.49, 3.39]	0.76 [0.29, 1.93]
	0.48 [0.15, 1.54]	PBO	0.92 [0.56, 1.48]	0.87 [0.55, 1.36]
	0.36 [0.05, 2.57]	0.76 [0.15, 3.20]	VDZ	0.95 [0.59, 1.52]
	0.86 [0.23, 3.49]	1.83 [0.98, 3.45]	2.40 [0.51, 15.2]	ADA
	Maintenance of Infection			
**Induction of Infection**	PBO M	1.11 [0.56, 2.15]	1.40 [0.92, 2.20]	1.51 [0.97, 2.42]
	1.04 [0.57, 1.87]	UST	1.31 [0.54, 3.21]	1.47 [0.62, 3.45]
	0.96 [0.54, 1.74]	0.92 [0.48, 1.82]	ADA I	1.11 [0.67, 1.92]
	1.23 [0.53, 3.05]	1.19 [0.43, 3.47]	1.30 [0.52, 3.28]	VDZ

Abbreviations: ADA; adalimumab, PBO; placebo, TEAE; treatment emergent adverse event, TESAE; treatment emergent serious adverse event, UST; ustekinumab, VDZ; vedolizumab.

Note: Comparison should be read from left to right.

^I^: The 1^st^ rank drug of induction phase.

^M^: The 1^st^ rank drug of maintenance phase.

### Safety: TESAE

In ADA, UST, and VDZ, there was no statistically significant difference in TESAE incidence compared to PBO in both induction and maintenance phases. In comparison between biologics, there was no statistically significant difference in TESAE incidence in both induction and maintenance phases as well. The 1^st^ rank drug for TESAE was identified as UST in both induction and maintenance phases. (Table 4, Table 6 in S6 File).

### Safety: Infection

In ADA, UST, and VDZ, there was no statistically significant difference in infection incidence compared to PBO in both induction and maintenance phases. In comparison between biologics, there was no statistically significant difference in infection incidence in both induction and maintenance phases as well. The 1^st^ rank drug for infection was identified as ADA in the induction phase and PBO in maintenance phase. Excluding PBO, UST was considered as the top ranked drug among the biologics in the maintenance phase. (Table 4, Table 7 in S6 File).

### Risk of bias assessment

The risk of bias of the 10 clinical trials that were included in this analysis was evaluated using ROB 2 (S7 File).

VISIBLE1 trial was evaluated that there was some concern on bias arising from the randomization process, because the induction phase is open-label. VARSITY trial was evaluated that there was some concern on bias in selection of the reported result, because the results applied with non-responder imputation was not reported despite the absence of maintenance phase entry criteria. Lastly Motoya et al. and VISIBLE1 trials were evaluated that there was some concern on overall bias though it is not clear but the number of patients per treatment group was relatively small, at about 50.

All clinical trials included in this study were evaluated to have low risk of bias on overall bias.

### Sensitivity analysis

To evaluate the effect of race on the biologics’ efficacy and safety, sensitivity analyses were performed by excluding Motoya et al. 2019 and Suzuki et al. 2014 trials [30,35], respectively. A sensitivity analysis was also performed by excluding the both 2 trials. Forest plots of each endpoint generally showed the same trend as the forest plots of the main analysis. All 1^st^ rank drugs of each endpoint was same as the 1^st^ rank drugs of the main analysis. As a result, it was confirmed that the influence of race was insignificant (S8 File).

## Discussion/Conclusion

The results of the network meta-analysis did not reveal statistically significant differences among biologic agents. However, the ranking information for each biologic agent exhibited the patterns. Including histologic remission, for the overall efficacy endpoints in the maintenance phase, VDZ was identified as the 1^st^ rank drug.

In addition, due to the following limitations, there may be some difficulties in generalizing the results of this study.

Due to the results of histologic assessment of studies were limited, the number of selected studies was small. There was heterogeneity in key eligibility and concomitant medication utilization of patients included in this analysis. HIBISCUS1 and 2 trials [35,36] were conducted only on biologic naïve patients, but other clinical trials included not only biologic naïve patients but also patients who had failed TNF⍺-I or biologics. In addition, for concomitant medication that may affect efficacy outcome, the percentage of patients between treatment arms within the study were similar, but the one between studies were varied. There were heterogeneities in definition and index of histologic remission. Although the current definition of histologic remission is not clear, they all included the findings of the absence of neutrophils in epithelium as well as erosion or ulceration. Definition of histologic remission used in VDZ studies was the most conservative. In all selected studies, the diagnosis criteria for moderate to severe UC and efficacy endpoints used in this study could not reflected the latest established recommendation. Diagnosis for moderate to severe UC and clinical remission was defined by not modified Mayo score but original Mayo score, including physician’s global assessment. Corticosteroid-free remission also did not include pre-specified duration for corticosteroid-free before assessment. Lastly there was a limitation in general safety analysis. In this analysis, only infections that required close monitoring due to the association of biologic therapy was included. AEs of special interest other than infection were not included in this analysis due to the data limitation and very small number of events. In other words, the safety results of this analysis need attention in interpretation including comprehensive safety results, regardless of their causal relationship to drugs from the analysis. Furthermore, considering that the protocol was developed during the paper submission process after the completion of this study, the potential biases arising from the absence of a protocol in this study also serve as limitations of the present research.

However, the significance of this study lies in the fact that it is the first study to attempt an indirect comparison of histologic remission of biologics agents in UC, using NMA. This analysis is anticipated to provide more specific and reliable evidence for selecting drugs to be compared in the head-to-head study of biologics, as well as serve as a resource for moderate to severe UC treatment decisions in the real world. If future research is conducted to address the limitations of this study, it will be possible to obtain stronger evidence for the selection of biologics when histologic remission is the primary treatment objective. As histologic remission is the efficacy endpoint for all currently unapproved drugs, it is also anticipated that this study can be used to appropriately compare the efficacy of the conventional treatment with novel agents.

## Supporting information

S1 File PRISMA NMA checklist. (DOCX)

S2 File PICO-SD framework. (DOCX)

S3 File Search strategies. (DOCX)

S4 File List of excluded studies. (DOCX)

S5 File Definitions of efficacy and safety outcome. (DOCX)

S6 File Network Meta-analysis result. (DOCX)

S7 File Risk of bias 2 assessment. (DOCX)

S8 File Network Meta-analysis result of sensitivity analysis. (DOCX)

## Abbreviations

ADA: adalimumab, CrI; credible interval
FDA: U.S Food and Drug Administration
GOL: golimumab
IFX: infliximab
NMA: network meta-analysis
OR: odd ratio
PBO: placebo
RCT: randomized controlled trial
RoB: risk of bias
TEAE: treatment emergent adverse event
TESAE: treatment emergent serious adverse event
UST: ustekinumab
UC: ulcerative colitis
VDZ: vedolizumab

## References

[1] Cosnes J., Gower–Rousseau C., Seksik P., & Cortot A. (2011). Epidemiology and the natural history of inflammatory bowel diseases. *Gastroenterology*, 140(6), 1785–1794. 21530745 10.1053/j.gastro.2011.01.055

[2] Danese S., & Fiocchi C. (2011). Medical progress in treating ulcerative colitis. *New England Journal of Medicine*, 365(18), 1713–1725. 22047562 10.1056/NEJMra1102942

[3] Ford A. C., Moayyedi P., & Hanauer S. B. (2013). Management of ulcerative colitis shows the failings in chronic disease management in the current NHS. *Br*. *Med*. *J*, 346, f432. 23444437 10.1136/bmj.f1189

[4] Fumery M., Singh S., Dulai P. S., Gower-Rousseau C., Peyrin-Biroulet L., & Sandborn W. J. (2018). Natural history of adult ulcerative colitis in population-based cohorts: a systematic review. *Clinical Gastroenterology and Hepatology*, 16(3), 343–356. doi: 10.1016/j.cgh.2017.06.016 28625817 PMC6658168

[5] Peyrin‐Biroulet L., Germain A., Patel A. S., & Lindsay J. O. (2016). Systematic review: outcomes and post‐operative complications following colectomy for ulcerative colitis. Alimentary pharmacology & therapeutics, 44(8), 807–816. doi: 10.1111/apt.13763 27534519

[6] Peyrin-Biroulet L., Sandborn W., Sands B. E., Reinisch W., Bemelman W., Bryant R. V., et al. (2015). Selecting therapeutic targets in inflammatory bowel disease (STRIDE): determining therapeutic goals for treat-to-target. Official journal of the American College of Gastroenterology| ACG, 110(9), 1324–1338. doi: 10.1038/ajg.2015.233 26303131

[7] Pineton de Chambrun G., Peyrin-Biroulet L., Lémann M., & Colombel J. F. (2010). Clinical implications of mucosal healing for the management of IBD. *Nature reviews Gastroenterology & hepatology*, 7(1), 15–29. doi: 10.1038/nrgastro.2009.203 19949430

[8] Boal Carvalho P., & Cotter J. (2017). Mucosal healing in ulcerative colitis: a comprehensive review. *Drugs*, 77(2), 159–173. doi: 10.1007/s40265-016-0676-y 28078646

[9] Park S., Abdi T., Gentry M., & Laine L. (2016). Histological disease activity as a predictor of clinical relapse among patients with ulcerative colitis: systematic review and meta-analysis. *Official journal of the American College of Gastroenterology|* *ACG*, 111(12), 1692–1701. 10.1038/ajg.2016.418 27725645

[10] Rubin DT, Ananthakrishnan AN, Siegel CA, Sauer BG, Long MD. ACG Clinical Guideline: Ulcerative Colitis in Adults. Am J Gastroenterol. 2019;114(3):384–413. doi: 10.14309/ajg.0000000000000152 . 30840605

[11] Feuerstein JD, Isaacs KL, Schneider Y, Siddique SM, Falck-Ytter Y, Singh S, et al. AGA Clinical Practice Guidelines on the Management of Moderate to Severe Ulcerative Colitis. Gastroenterology. 2020;158(5):1450–61. Epub 20200113. doi: 10.1053/j.gastro.2020.01.006 ; PubMed Central PMCID: PMC7175923. 31945371 PMC7175923

[12] Lamb CA, Kennedy NA, Raine T, Hendy PA, Smith PJ, Limdi JK, et al. British Society of Gastroenterology consensus guidelines on the management of inflammatory bowel disease in adults. Gut. 2019;68(Suppl 3):s1–s106. Epub 20190927. doi: 10.1136/gutjnl-2019-318484 ; PubMed Central PMCID: PMC6872448. 31562236 PMC6872448

[13] Peyrin-Biroulet L, Sandborn W, Sands BE, Reinisch W, Bemelman W, Bryant RV, et al. Selecting Therapeutic Targets in Inflammatory Bowel Disease (STRIDE): Determining Therapeutic Goals for Treat-to-Target. Am J Gastroenterol. 2015;110(9):1324–38. Epub 20150825. doi: 10.1038/ajg.2015.233 . 26303131

[14] Turner D, Ricciuto A, Lewis A, D’Amico F, Dhaliwal J, Griffiths AM, et al. STRIDE-II: An Update on the Selecting Therapeutic Targets in Inflammatory Bowel Disease (STRIDE) Initiative of the International Organization for the Study of IBD (IOIBD): Determining Therapeutic Goals for Treat-to-Target strategies in IBD. Gastroenterology. 2021;160(5):1570–83. Epub 20210219. doi: 10.1053/j.gastro.2020.12.031 . 33359090

[15] Park S, Abdi T, Gentry M, Laine L. Histological Disease Activity as a Predictor of Clinical Relapse Among Patients With Ulcerative Colitis: Systematic Review and Meta-Analysis. Am J Gastroenterol. 2016;111(12):1692–701. Epub 20161011. doi: 10.1038/ajg.2016.418 . 27725645

[16] Bryant RV, Burger DC, Delo J, Walsh AJ, Thomas S, von Herbay A, et al. Beyond endoscopic mucosal healing in UC: histological remission better predicts corticosteroid use and hospitalisation over 6 years of follow-up. Gut. 2016;65(3):408–14. Epub 20150518. doi: 10.1136/gutjnl-2015-309598 . 25986946

[17] U.S Food and Drug Administration (2022). Ulcerative Colitis: Developing Drugs for Treatment Guidance for Industry https://www.fda.gov/regulatory-information/search-fda-guidance-documents/ulcerative-colitis-developing-drugs-treatment.

[18] Sands BE, Peyrin-Biroulet L, Loftus EV Jr, Danese S Colombel JF, Toruner M, et al. Vedolizumab versus Adalimumab for Moderate-to-Severe Ulcerative Colitis. N Engl J Med. 2019;381(13):1215–26. doi: 10.1056/NEJMoa1905725 . 31553834

[19] PRISMA (2015). PRISMA for Network Meta-Analyses http://www.prisma-statement.org/Extensions/NetworkMetaAnalysis.

[20] Eriksen M. B., & Frandsen T. F. (2018). The impact of patient, intervention, comparison, outcome (PICO) as a search strategy tool on literature search quality: a systematic review. *Journal* of the Medical Library Association: JMLA, 106(4), 420. doi: 10.5195/jmla.2018.345 30271283 PMC6148624

[21] Protocol of An Indirect Comparison of Efficacy including Histologic Assessment and Safety in Biologic Agents in Ulcerative Colitis: Systematic Review and Network Meta-analysis V.2 doi: 10.17504/protocols.io.q26g7y8dkgwz/v237917756

[22] Mosli MH, Feagan BG, Zou G, Sandborn WJ, D’Haens G, Khanna R, et al. Development and validation of a histological index for UC. Gut. 2017;66(1):50–8. Epub 20151016. doi: 10.1136/gutjnl-2015-310393 . 26475633

[23] Marchal-Bressenot A, Salleron J, Boulagnon-Rombi C, Bastien C, Cahn V, Cadiot G, et al. Development and validation of the Nancy histological index for UC. Gut. 2017;66(1):43–9. Epub 20151013. doi: 10.1136/gutjnl-2015-310187 . 26464414

[24] Schroeder KW, Tremaine WJ, Ilstrup DM. Coated oral 5-aminosalicylic acid therapy for mildly to moderately active ulcerative colitis. A randomized study. N Engl J Med. 1987;317(26):1625–9. doi: 10.1056/NEJM198712243172603 . 3317057

[25] Sterne JAC, Savovic J, Page MJ, Elbers RG, Blencowe NS, Boutron I, et al. RoB 2: a revised tool for assessing risk of bias in randomised trials. BMJ. 2019;366:l4898. Epub 20190828. doi: 10.1136/bmj.l4898 . 31462531

[26] Hu D O’Connor AM, Wang C, Sargeant JM, Winder CB. How to Conduct a Bayesian Network Meta-Analysis. Front Vet Sci. 2020;7:271. Epub 20200519. doi: 10.3389/fvets.2020.00271 ; PubMed Central PMCID: PMC7248597. 32509807 PMC7248597

[27] Sands BE, Sandborn WJ, Panaccione R, O’Brien CD, Zhang H, Johanns J, et al. Ustekinumab as Induction and Maintenance Therapy for Ulcerative Colitis. N Engl J Med. 2019;381(13):1201–14. doi: 10.1056/NEJMoa1900750 . 31553833

[28] Li K, Marano C, Zhang H, Yang F, Sandborn WJ, Sands BE, et al. Relationship Between Combined Histologic and Endoscopic Endpoints and Efficacy of Ustekinumab Treatment in Patients With Ulcerative Colitis. Gastroenterology. 2020;159(6):2052–64. Epub 20200825. doi: 10.1053/j.gastro.2020.08.037 . 32853634

[29] Feagan BG, Rutgeerts P, Sands BE, Hanauer S, Colombel JF, Sandborn WJ, et al. Vedolizumab as induction and maintenance therapy for ulcerative colitis. N Engl J Med. 2013;369(8):699–710. doi: 10.1056/NEJMoa1215734 . 23964932

[30] Motoya S, Watanabe K, Ogata H, Kanai T, Matsui T, Suzuki Y, et al. Vedolizumab in Japanese patients with ulcerative colitis: A Phase 3, randomized, double-blind, placebo-controlled study. PLoS One. 2019;14(2):e0212989. Epub 20190226. doi: 10.1371/journal.pone.0212989 ; PubMed Central PMCID: PMC6391030. 30807613 PMC6391030

[31] Peyrin-Biroulet L, Loftus EV Jr, Colombel JF, Danese S, Rogers R, Bornstein JD, et al. Histologic Outcomes With Vedolizumab Versus Adalimumab in Ulcerative Colitis: Results From An Efficacy and Safety Study of Vedolizumab Intravenous Compared to Adalimumab Subcutaneous in Participants With Ulcerative Colitis (VARSITY). Gastroenterology. 2021;161(4):1156–67 e3. Epub 20210616. doi: 10.1053/j.gastro.2021.06.015 . 34144047

[32] Sandborn WJ, Baert F, Danese S, Krznaric Z, Kobayashi T, Yao X, et al. Efficacy and Safety of Vedolizumab Subcutaneous Formulation in a Randomized Trial of Patients With Ulcerative Colitis. Gastroenterology. 2020;158(3):562–72 e12. Epub 20190828. doi: 10.1053/j.gastro.2019.08.027 . 31470005

[33] Reinisch W, Sandborn WJ, Hommes DW, D’Haens G, Hanauer S, Schreiber S, et al. Adalimumab for induction of clinical remission in moderately to severely active ulcerative colitis: results of a randomised controlled trial. Gut. 2011;60(6):780–7. Epub 20110105. doi: 10.1136/gut.2010.221127 . 21209123

[34] Sandborn WJ, Colombel JF, D’Haens G, Van Assche G, Wolf D, Kron M, et al. One-year maintenance outcomes among patients with moderately-to-severely active ulcerative colitis who responded to induction therapy with adalimumab: subgroup analyses from ULTRA 2. Aliment Pharmacol Ther. 2013;37(2):204–13. Epub 20121123. doi: 10.1111/apt.12145 . 23173821

[35] Suzuki Y, Motoya S, Hanai H, Matsumoto T, Hibi T, Robinson AM, et al. Efficacy and safety of adalimumab in Japanese patients with moderately to severely active ulcerative colitis. J Gastroenterol. 2014;49(2):283–94. Epub 20131224. doi: 10.1007/s00535-013-0922-y ; PubMed Central PMCID: PMC3925299. 24363029 PMC3925299

[36] Rubin DT, Dotan I, DuVall A, Bouhnik Y, Radford-Smith G, Higgins PDR, et al. Etrolizumab versus adalimumab or placebo as induction therapy for moderately to severely active ulcerative colitis (HIBISCUS): two phase 3 randomised, controlled trials. Lancet Gastroenterol Hepatol. 2022;7(1):17–27. Epub 20211117. doi: 10.1016/S2468-1253(21)00338-1 . 34798036

