# Supplementary material for: An indirect comparison of efficacy including histologic assessment and safety in biologic therapy in ulcerative colitis: Systemic review and network meta-analysis
Source: PLoS One. 2023 Nov 2;18(11):e0293655. doi: 10.1371/journal.pone.0293655 (PMC10621919; doi:10.1371/journal.pone.0293655)
Supplement: S2 File — (DOCX) [file pone.0293655.s002.docx]

PICO framework

| **Parameter** | **Contents** |
| --- | --- |
| Population | Moderate to severe ulcerative colitis |
| Intervention | Infliximab, Adalimumab, Vedolizumab, Golimumab, Ustekinumab, Placebo |
| Comparator |  |
| Outcome | Clinical remission, Endoscopic improvement, Histologic remission, overall safety |
| Study design | Randomized controlled trials |

Abbreviation: PICO; population, intervention, comparator, outcome, and study design
