# Supplementary material for: An indirect comparison of efficacy including histologic assessment and safety in biologic therapy in ulcerative colitis: Systemic review and network meta-analysis
Source: PLoS One. 2023 Nov 2;18(11):e0293655. doi: 10.1371/journal.pone.0293655 (PMC10621919; doi:10.1371/journal.pone.0293655)
Supplement: S3 File — (DOCX) [file pone.0293655.s003.docx]

Search Strategies

| **Pubmed** | |
| --- | --- |
| **P** | "colitis, ulcerative"[MeSH Terms] OR ("colitis"[All Fields] AND "ulcerative"[All Fields]) OR "ulcerative colitis"[All Fields] OR ("colitis"[All Fields] AND "ulcerative"[All Fields]) OR "colitis ulcerative"[All Fields] |
| **I/C** | "infliximab"[MeSH Terms] OR "infliximab"[All Fields] OR "infliximab s"[All Fields] OR "adalimumab"[MeSH Terms] OR "adalimumab"[All Fields] OR "golimumab"[Supplementary Concept] OR "golimumab"[All Fields] OR "golimumab s"[All Fields] OR "vedolizumab"[Supplementary Concept] OR "vedolizumab"[All Fields] OR "ustekinumab"[MeSH Terms] OR "ustekinumab"[All Fields] OR "placeboes"[All Fields] OR "placebos"[MeSH Terms] OR "placebos"[All Fields] OR "placebo"[All Fields] |
| **O** | "efficacies"[All Fields] OR "efficacious"[All Fields] OR "efficaciously"[All Fields] OR "efficaciousness"[All Fields] OR "efficacy"[All Fields] OR (("ambulatory care facilities"[MeSH Terms] OR ("ambulatory"[All Fields] AND "care"[All Fields] AND "facilities"[All Fields]) OR "ambulatory care facilities"[All Fields] OR "clinic"[All Fields] OR "clinic s"[All Fields] OR "clinical"[All Fields] OR "clinically"[All Fields] OR "clinicals"[All Fields] OR "clinics"[All Fields]) AND ("remission"[All Fields] OR "remissions"[All Fields])) OR (("endoscope s"[All Fields] OR "endoscoped"[All Fields] OR "endoscopes"[MeSH Terms] OR "endoscopes"[All Fields] OR "endoscope"[All Fields] OR "endoscopical"[All Fields] OR "endoscopically"[All Fields] OR "endoscopy"[MeSH Terms] OR "endoscopy"[All Fields] OR "endoscopic"[All Fields]) AND ("remission"[All Fields] OR "remissions"[All Fields])) OR (("histologic"[All Fields] OR "histological"[All Fields] OR "histologically"[All Fields]) AND ("remission"[All Fields] OR "remissions"[All Fields])) OR (("mucosalization"[All Fields] OR "mucosalized"[All Fields] OR "mucosally"[All Fields] OR "mucose"[All Fields] OR "mucoses"[All Fields] OR "mucositis"[MeSH Terms] OR "mucositis"[All Fields] OR "mucositides"[All Fields] OR "mucous membrane"[MeSH Terms] OR ("mucous"[All Fields] AND "membrane"[All Fields]) OR "mucous membrane"[All Fields] OR "mucosal"[All Fields]) AND ("healed"[All Fields] OR "wound healing"[MeSH Terms] OR ("wound"[All Fields] AND "healing"[All Fields]) OR "wound healing"[All Fields] OR "healing"[All Fields] OR "healings"[All Fields] OR "heals"[All Fields])) OR ("biopsie"[All Fields] OR "biopsy"[MeSH Terms] OR "biopsy"[All Fields] OR "biopsied"[All Fields] OR "biopsies"[All Fields] OR "biopsy s"[All Fields] OR "biopsying"[All Fields] OR "biopsys"[All Fields] OR "pathology"[MeSH Subheading] OR "pathology"[All Fields]) OR "endo*"[All Fields] OR "histo*"[All Fields] OR ("remission"[All Fields] OR "remissions"[All Fields]) OR ("safety"[MeSH Terms] OR "safety"[All Fields] OR "safeties"[All Fields]) |
| **SD** | "random*"[All Fields] OR ("controlled"[All Fields] AND ("clinical trials as topic"[MeSH Terms] OR ("clinical"[All Fields] AND "trials"[All Fields] AND "topic"[All Fields]) OR "clinical trials as topic"[All Fields] OR "trial"[All Fields] OR "trial s"[All Fields] OR "trialed"[All Fields] OR "trialing"[All Fields] OR "trials"[All Fields])) OR ("clinical trial"[Publication Type] OR "clinical trials as topic"[MeSH Terms] OR "clinical trial"[All Fields]) OR ("clinical trials as topic"[MeSH Terms] OR ("clinical"[All Fields] AND "trials"[All Fields] AND "topic"[All Fields]) OR "clinical trials as topic"[All Fields] OR "trial"[All Fields] OR "trial s"[All Fields] OR "trialed"[All Fields] OR "trialing"[All Fields] OR "trials"[All Fields]) |
| **EMBASE** | |
| **P** | 'ulcerative colitis'/exp OR 'ulcerative colitis' |
| **I/C** | 'infliximab’ OR ‘adalimumab’ OR ‘golimumab’ OR ‘vedolizumab’ OR ‘ustekinumab’ |
| **O** | 'efficacy' OR 'clinical remission' OR 'endoscopic remission' OR 'histologic remission' OR 'mucosal healing' OR 'biopsy' OR 'remission' OR 'safety' |
| **SD** | 'randomized controlled trial'/exp OR 'controlled trial, randomized' OR 'randomised controlled study' OR 'randomised controlled trial' OR 'randomized controlled study' OR 'randomized controlled trial' OR 'trial, randomized controlled' |
| **The Cochrane Library** | |
| **P** | colitis, ulcerative [MeSH Term] |
| **I/C** | (infliximab) OR (adalimumab) OR (golimumab) OR (vedolizumab) OR (ustekinumab) |
| **O** | '(efficacy) OR (clinical remission) OR (endoscopic remission) OR (histologic remission) OR (mucosal healing) OR (biopsy) OR (endo*) OR (histo*) OR (remission) OR (safety) |
| **SD** | 'randomized controlled trial OR controlled trial, randomized OR randomised controlled study OR randomised controlled trial OR randomized controlled study OR randomized controlled trial OR trial, randomized controlled |
| **Clinicaltrial.gov** | |
| **P** | 'ulcerative colitis |
| **I** | 'infliximab OR adalimumab OR golimumab OR vedolizumab OR ustekinumab |
| **SD** | interventional studies |

Abbreviations: C; comparator, I; intervention, SD; study design, P; patient
