# Supplementary material for: An indirect comparison of efficacy including histologic assessment and safety in biologic therapy in ulcerative colitis: Systemic review and network meta-analysis
Source: PLoS One. 2023 Nov 2;18(11):e0293655. doi: 10.1371/journal.pone.0293655 (PMC10621919; doi:10.1371/journal.pone.0293655)
Supplement: S4 File — (DOCX) [file pone.0293655.s004.docx]

**Excluded studies**

Due to its volume, we could not describe all excluded papers, but we present representative excluded papers herein

| Author | Title | Rationale |
| --- | --- | --- |
| Abreu MT, Rowbotham DS, Danese S, Sandborn WJ, Miao Y, Zhang H, Tikhonov I, Panaccione R, Hisamatsu T, Scherl EJ, Leong RW, Arasaradnam RP, Afif W, Peyrin-Biroulet L, Sands BE, Marano C. | Efficacy and Safety of Maintenance Ustekinumab for Ulcerative Colitis Through 3 Years: UNIFI Long-term Extension | Unmet study design (not RCT, retrospective, prospective, observational, switching, case study) |
| Schreiber S., Galinsky K., Aubrecht J., Juarez J., Agboton C., Loftus E.V., Danese S. | Early modification of inflammatory burden through treatment with vedolizumab or adalimumab is predictive of long-term treatment success in patients with Ulcerative Colitis from the VARSITY Study | Review / Meta-Analysis / Survey/ Poster |
| Danese S, Sands BE, Abreu MT, O'Brien CD, Bravatà I, Nazar M, Miao Y, Wang Y, Rowbotham D, Leong RWL, Arasaradnam RP, Afif W, Marano C. | Early Symptomatic Improvement After Ustekinumab Therapy in Patients With Ulcerative Colitis: 16-Week Data From the UNIFI Trial | Review / Meta-Analysis / Survey/ Poster |
| Sands B.E., Feagan B.G., Sandborn W.J., Shipitofsky N., Marko M., Sheng S., Johanns J., Germinaro M., Vetter M., Vega P.J. | Efficacy and safety of combination induction therapy with guselkumab and golimumab in participants with moderately-to-severely active Ulcerative Colitis: Results through week 12 of a phase 2a randomized, double-blind, activecontrolled, parallel-group, multicenter, proof-ofconcept study | Result was not available (only Abstract / Letter / no Result / ongoing trial / not English) |
| Panés J., Colombel J.-F., D'Haens G.R., Schreiber S., Panaccione R., Peyrin-Biroulet L., Loftus E.V., Danese S., Tanida S., Okuyama Y., Louis E., Armuzzi A., Ferrante M., Vogelsang H., Hibi T., Watanabe M., Lefebvre J., Finney-Hayward T., Gonzalez Y.S., Doan T.T., Mostafa N.M., Ikeda K., Xie W., Huang B., Petersson J., Kalabic J., Robinson A.M., Sandborn W.J. | Higher vs Standard Adalimumab Induction and Maintenance Dosing Regimens for Treatment of Ulcerative Colitis: SERENE UC Trial Results | Unmet intervention (not ADA or VDZ or UST, biosimilar, combination) |
| Dulai PS, Wong ECL, Reinisch W, Colombel JF, Marshall JK, Narula N. | Decision Support Tool Identifies Ulcerative Colitis Patients Most Likely to Achieve Remission With Vedolizumab vs Adalimumab | Review / Meta-Analysis / Survey/ Poster |
| Kobayashi T, Ito H, Ashida T, Yokoyama T, Nagahori M, Inaba T, Shikamura M, Yamaguchi T, Hori T, Pinton P, Watanabe M, Hibi T. | Efficacy and safety of a new vedolizumab subcutaneous formulation in Japanese patients with moderately to severely active ulcerative colitis | Review / Meta-Analysis / Survey/ Poster |
| Nagahori M, Watanabe K, Motoya S, Ogata H, Kanai T, Matsui T, Suzuki Y, Pinton P, Ursos L, Sakamoto S, Shikamura M, Hori T, Fernandez J, Hibi T, Watanabe M. | Week 2 Symptomatic Response with Vedolizumab as a Predictive Factor in Japanese Anti-TNFα-Naive Patients with Ulcerative Colitis: A post hoc Analysis of a Randomized, Placebo-Controlled Phase 3 Trial | Review / Meta-Analysis / Survey/ Poster |
| Abreu M.T., Rowbotham D.S., Danese S., Sandborn W.J., Miao Y., Zhang H., Panaccione R., Hisamatsu T., Scherl E., Leong R., Arasaradnam R., Afif W., Peyrin-Biroulet L., Sands B.E., Marano C. | Efficacy of ustekinumab for ulcerative colitis in patients through 3 years: Unifi long-term extension | Unmet study design (not RCT, retrospective, prospective, observational, switching, case study) |
| Sandborn WJ, Feagan BG, Danese S, O'Brien CD, Ott E, Marano C, Baker T, Zhou Y, Volger S, Tikhonov I, Gasink C, Sands BE, Ghosh S. | Safety of Ustekinumab in Inflammatory Bowel Disease: Pooled Safety Analysis of Results from Phase 2/3 Studies | Review / Meta-Analysis / Survey/ Poster |
| Hisamatsu T, Kim HJ, Motoya S, Suzuki Y, Ohnishi Y, Fujii N, Matsushima N, Zheng R, Marano CW. | Efficacy and safety of ustekinumab in East Asian patients with moderately to severely active ulcerative colitis: a subpopulation analysis of global phase 3 induction and maintenance studies (UNIFI) | Review / Meta-Analysis / Survey/ Poster |
| Danese S., Costa Sa A.C., Marano C., Sands B., Peyrin-Biroulet L. | Effects of ustekinumab induction and maintenance therapy on disease clearance in the UNIFI phase 3 study in ulcerative colitis | Review / Meta-Analysis / Survey/ Poster |
| Ferrante M., Pivorunas V., Butler J., Mahi N., Mostafa N.M., Ruzek M., Fann J., Hong F., Cao X., Smaoui N., Davis J.W., Kalabic J., Song A., Guay H., Rieder F., Panés J., Colombel J.-F. | A higher induction dosing regimen of adalimumab does not enhance modulation of downstream blood molecular markers in patients with moderately to severely active crohn's disease or ulcerative colitis: Results from the serene-cd and serene-uc studies | Review / Meta-Analysis / Survey/ Poster |
| Chen J, Hunter S, Kisfalvi K, Lirio RA. | A hybrid approach of handling missing data under different missing data mechanisms: VISIBLE 1 and VARSITY trials for ulcerative colitis | Review / Meta-Analysis / Survey/ Poster |
| Nuñez F P., Mahadevan U., Quera R., Bay C., Ibañez P. | Treat-to-target approach in the management of inflammatory Bowel disease | Review / Meta-Analysis / Survey/ Poster |
| Abreu M.T., Peyrin-Biroulet L., Danese S., Marano C., Zhou Y., Zhang H., Leong R.W.L., Rowbotham D., Panaccione R., Sandborn W.J. | Effect of Ustekinumab Maintenance Therapy on Stool Frequency and Rectal Bleeding Through 2 Years in the UNIFI Phase 3 Study in Ulcerative Colitis | Unmet study design (not RCT, retrospective, prospective, observational, switching, case study) |
| Li K., Yang F., Marano C.W., Zhang H., Sandborn W.J., Sands B.E., Feagan B.G., Rubin D.T., Peyrin-Biroulet L., Friedman J., De Hertogh G. | ASSOCIATION OF HISTOLOGIC-ENDOSCOPIC MUCOSAL HEALING AFTER USTEKINUMAB INDUCTION OR MAINTENANCE THERAPY WITH 2-YEAR OUTCOMES IN THE UNIFI PHASE 3 STUDY IN ULCERATIVE COLITIS | Unmet study design (not RCT, retrospective, prospective, observational, switching, case study) |
| Abreu M.T., Sands B.E., Leong R.W., Marano C.W., O'Brien C.D., Zhang H., Zhou Y., Johanns J., Rowbotham D., Hisamatsu T., Arasaradnam R.P., Scherl E.J., Danese S., Peyrin-Biroulet L. | EFFICACY AND SAFETY OF LONG-TERM TREATMENT WITH USTEKINUMAB IN MODERATE-SEVERE ULCERATIVE COLITIS PATIENTS WITH DELAYED RESPONSE TO USTEKINUMAB INDUCTION: RESULTS FROM THE UNIFI 2-YEAR LONG-TERM EXTENSION | Unmet study design (not RCT, retrospective, prospective, observational, switching, case study) |
| Loftus E.V., Schreiber S., Danese S., Peyrin-Biroulet L., Colombel J.F., Sands B.E., Wang S., Chen J., Lirio R.A. | PATIENT-REPORTED HEALTH-RELATED QUALITY OF LIFE OUTCOMES WITH VEDOLIZUMAB VERSUS ADALIMUMAB TREATMENT OF ULCERATIVE COLITIS: RESULTS OF THE VARSITY TRIAL | Review / Meta-Analysis / Survey/ Poster |
| Sands B.E., Sandborn W.J., Panaccione R., O'Brien C.D., Zhang H., Johanns J., Zhou Y., Peyrin-Biroulet L., Scherl E.J., Leong R.W., Rowbotham D., Arasaradnam R.P., Hisamatsu T., Abreu M.T., Danese S., Marano C.W. | EFFICACY OF USTEKINUMAB FOR ULCERATIVE COLITIS IN BIOLOGIC NAÏVE, BIOLOGIC NON-FAILURE, AND BIOLOGIC FAILURE POPULATIONS THROUGH 2 YEARS: UNIFI LONG-TERM EXTENSION | Unmet study design (not RCT, retrospective, prospective, observational, switching, case study) |
| Sandborn W.J., Danese S., Jansson J.P., Chen J., Uddin S.M., Candela N., Lasch K., Kisfalvi K. | EFFICACY AND SAFETY OF VEDOLIZUMAB REINITIATION FOLLOWING TREATMENT INTERRUPTION: POST HOC ANALYSIS OF THE VISIBLE TRIAL DATA | Unmet intervention (not ADA or VDZ or UST, biosimilar, combination) |
| Sandborn W.J., Sands B.E., Panaccione R., Marano C.W., O'Brien C.D., Zhang H., Johanns J., Zhou Y., Peyrin-Biroulet L., Hisamatsu T., Danese S. | EFFICACY OF USTEKINUMAB FOR ULCERATIVE COLITIS THROUGH 2 YEARS: RESULTS OF THE UNIFI MAINTENANCE STUDY AND LONG-TERM EXTENSION | Unmet study design (not RCT, retrospective, prospective, observational, switching, case study) |
| Sandborn W.J., Peyrin-Biroulet L., Sands B.E., Scherl E.J., Marano C.W., O'Brien C.D., Zhang H., Johanns J., Zhou Y., Abreu M.T., Arasaradnam R.P., Rowbotham D., Leong R.W., Danese S. | CORTICOSTEROID SPARING EFFECTS OF USTEKINUMAB THERAPY FOR ULCERATIVE COLITIS THROUGH 2 YEARS: UNIFI LONG-TERM EXTENSION | Unmet study design (not RCT, retrospective, prospective, observational, switching, case study) |
| Adedokun OJ, Xu Z, Marano C, O'Brien C, Szapary P, Zhang H, Johanns J, Leong RW, Hisamatsu T, Van Assche G, Danese S, Abreu MT, Sands BE, Sandborn WJ. | Ustekinumab Pharmacokinetics and Exposure Response in a Phase 3 Randomized Trial of Patients With Ulcerative Colitis | Review / Meta-Analysis / Survey/ Poster |
| Xu Y, Hu C, Chen Y, Miao X, Adedokun OJ, Xu Z, Sharma A, Zhou H. | Population Pharmacokinetics and Exposure-Response Modeling Analyses of Ustekinumab in Adults With Moderately to Severely Active Ulcerative Colitis | Review / Meta-Analysis / Survey/ Poster |
| Colombel J.-F., Panés J., D'Haens G.R., Schreiber S., Panaccione R., Peyrin-Biroulet L., Loftus E.V., Danese S., Louis E., Armuzzi A., Ferrante M., Vogelsang H., Mostafa N., Doan T., Wangang X., Petersson J., Kalabic J., Robinson A., Sandborn W.J. | Therapeutic Drug Monitoring Dosing Regimen with Adalimumab in Patients with Moderately to Severely Active Ulcerative Colitis: Results from the SERENE-UC Maintenance Study | Review / Meta-Analysis / Survey/ Poster |
| Tanida S., Okuyama Y., Watanabe M., Hibi T., Kobayashi K., Doan T., Ikeda K., Huang B., Petersson J., Kalabic J., Robinson A.M., Panés J. | Higher vs standard adalimumab induction and maintenance treatment in patients with moderately-to-severely active ulcerative colitis: Results from the phase 3 serene-UC Japan study | Review / Meta-Analysis / Survey/ Poster |
| Bhattacharya A., Osterman M.T. | Biologic Therapy for Ulcerative Colitis | Review / Meta-Analysis / Survey/ Poster |
| D’amico F., Danese S., Peyrin-Biroulet L. | Adaptive designs: Lessons for inflammatory bowel disease trials | Review / Meta-Analysis / Survey/ Poster |
| Pouillon L., Travis S., Bossuyt P., Danese S., Peyrin-Biroulet L. | Head-to-head trials in inflammatory bowel disease: past, present and future | Review / Meta-Analysis / Survey/ Poster |
| Johnson C., Barnes E.L., Zhang X., Long M.D. | Trends and characteristics of clinical trials participation for inflammatory bowel disease in the United States: A report from IBD partners | Review / Meta-Analysis / Survey/ Poster |
| Allen P.B., Bonovas S., Danese S., Peyrin-Biroulet L. | Evolving primary and secondary endpoints in randomized controlled trials leading to approval of biologics and small molecules in IBD: an historical perspective | Review / Meta-Analysis / Survey/ Poster |
| Sands B.E., Abreu M.T., Marano C., Baker T., O'Brien C., Zhang H., Johanns J., Rowbotham D., Leong R.W.L., Danese S. | Early improvement after intravenous ustekinumab induction in patients with ulcerative colitis: Results from the UNIFI induction trial | Review / Meta-Analysis / Survey/ Poster |
| Panaccione R., Peyrin-Biroulet L., Danese S., Marano C., O'Brien C.D., Szapary P., Zhang H., Johanns J., Leong R.W.L., Arasaradnam R.P., Rowbotham D., Abreu M.T., Sands B.E. | Impact of response and inflammatory burden at start of maintenance therapy on clinical efficacy of ustekinumab dosing regimen in UC: Week 44 results from UNIFI | Review / Meta-Analysis / Survey/ Poster |
| Sands B., Sandborn W., Pannacione R., O'Brien C., Leong R., Zhang H., Johanns J., Peyrin-Biroulet L., Van Assche G., Danese S., Targan S., Abreu M., Hisamatsu T., Szapary P., Marano C. | Efficacy and safety of ustekinumab as maintenance therapy in ulcerative colitis: Week 44 results from the UNIFI study | Review / Meta-Analysis / Survey/ Poster |
| Danese S., Sands B., O'Brien C., Zhang H., Johanns J., Sloan S., Izanek J., Szapary P., Marano C., Leong R., Rowbotham D., Targan S., Van Assche G. | Efficacy and safety of ustekinumab through week 16 in patients with moderate to severe ulcerative colitis: Results from the UNIFI induction trial | Review / Meta-Analysis / Survey/ Poster |
| Sands B., Peyrin-Biroulet L., Marano C., O'Brien C., Zhang H., Johanns J., Szapary P., Rowbotham D., Leong R., Arasaradnam R., Danese S., Van Assche G., Targan S., Sandborn W. | Efficacy in biological therapy failure and nonbiological therapy failure populations in a Phase III study of ustekinumab in moderate-severe ulcerative colitis: UNIFI study | Review / Meta-Analysis / Survey/ Poster |
| Hanauer S, Sandborn WJ, Colombel JF, Vermeire S, Petersson J, Kligys K, Zhou Q, Lazar A, Reinisch W. | Rapid Changes in Laboratory Parameters and Early Response to Adalimumab: A Pooled Analysis From Patients With Ulcerative Colitis in Two Clinical Trials | Review / Meta-Analysis / Survey/ Poster |
| Wolf D., Danese S., Matthews B., Jones S., Chen J., Kisfalvi K., Sandborn W.J. | Transitioning from vedolizumab iv to vedolizumab SC in patients with ulcerative colitis: Results from the visible program | Unmet intervention (not ADA or VDZ or UST, biosimilar, combination) |
| Li K., Yang F., Hayden K., Strawn D., Wadman E., Bhagat S., Marano C., Friedman J.R. | Molecular response to ustekinumab in moderate-to-severe ulcerative colitis by serum protein and colon transcriptomic analysis: Results from the UNIFI phase 3 maintenance study | Review / Meta-Analysis / Survey/ Poster |
| Panés J., Colombel J.-F., D'Haens G.R., Schreiber S., Panaccione R., Peyrin-Biroulet L., Loftus E., Danese S., Louis E., Armuzzi A., Ferrante M., Vogelsang H., Lefebvre J., Doan T., Kwatra N.V., Mostafa N.M., Xie W., Huang B., Petersson J., Kalabic J., Robinson A.M., Sandborn W.J. | High versus standard adalimumab induction dosing regime ns in patients with moderately to severely active ulcerative colitis : Results from the SERENE-UC induction study | Review / Meta-Analysis / Survey/ Poster |
| Danese S., Sands B.E., Peyrin-Biroulet L., Marano C., O'Brien C., Zhang H., Oortwijn A., Rowbotham D., Leong R.W.L., Arasaradnam R.P., Van Assche G., Sandborn W.J., Panaccione R. | Corticosteroid sparing effects of ustekinumab therapy in uc patients: Results from the UNIFI program | Review / Meta-Analysis / Survey/ Poster |
| Danese S., Loftus E.V., Colombel J.-F., Peyrin-Biroulet L., Abhyankar B., Chen J., Rogers R., Lirio R.A., Bornstein J.D., Schreiber S., Sands B.E. | Early clinical response and remission with vedolizumab versus adalimumab in ulcerative colitis: Results from varsity | Review / Meta-Analysis / Survey/ Poster |
| Adedokun O.J., Xu Z., Marano C., O'Brien C., Szapary P., Zhang H., Johanns J., Leong R.W.L., Hisamatsu T., Van Assche G., Danese S., Abreu M.T., Sands B.E., Sandborn W.J. | Pharmacokinetics and exposure-response relationships of ustekinumab in patients with ulcerative colitis: Results from the UNIFI induction and maintenance studies | Review / Meta-Analysis / Survey/ Poster |
| Danese S., Sands B.E., Leong R.W., Zhang H., Johanns J., Szapary P., Marano C., Han C. | General health status in patients with moderate to severe ulcerative colitis receiving ustekinumab: Results from the Phase 3 UNIFI induction and maintenance studies | Review / Meta-Analysis / Survey/ Poster |
| Danese S., Sands B., Leong R., Zhang H., Johanns J., Szapary P., Marano C., Han P. | General health status in patients with moderate to severe ulcerative colitis receiving ustekinumab: Results from the Phase III UNIFI induction and maintenance studies | Review / Meta-Analysis / Survey/ Poster |
| Adedokun O., Xu Z., Marano C., O'Brien C., Szapary P., Zhang H., Johanns J., Leong R., Hisamatsu T., Van Assche G., Danese S., Abreu M., Sands B., Sandborn W. | Pharmacokinetics and exposure-response relationships of intravenously administered ustekinumab during induction treatment in patients with ulcerative colitis: Results from the UNIFI induction study | Review / Meta-Analysis / Survey/ Poster |
| Sands B., Han C., Johanns J., Szapary P., Marano C., Leong R., Danese S. | Ustekinumab therapy induced clinically meaningful improvement and remission as measured by the inflammatory bowel disease questionnaire in patients with moderate to severe ulcerative colitis: Results from the phase III UNIFI induction and maintenance studies | Review / Meta-Analysis / Survey/ Poster |
| Sands B.E., Han C., Zhang H., Johanns J., Szapary P., Marano C., Leong R.W., Danese S. | Ustekinumab therapy induced clinically meaningful improvement and remission as measured by the Inflammatory Bowel Disease Questionnaire: Results from the phase 3 UNIFI induction and maintenance studies | Review / Meta-Analysis / Survey/ Poster |
| Sandborn W.J., Baert F., Danese S., Krznarić Z., D'Haens G., Kobayashi T., Yao X., Chen J., Kisfalvi K., Vermeire S. | Efficacy and safety of vedolizumab subcutaneous formulation for ulcerative colitis: Results of the visible trial | Review / Meta-Analysis / Survey/ Poster |
| Loftus E.V., Sandborn W.J., Wolf D.C., Danese S., Chen J., Yao X., Kisfalvi K., Vermeire S. | EFFICACY AND SAFETY OF 2 OR 3 VEDOLIZUMAB INTRAVENOUS INFUSIONS AS INDUCTION THERAPY FOR ULCERATIVE COLITIS AND CROHN'S DISEASE: RESULTS FROM VISIBLE 1 AND 2 | Review / Meta-Analysis / Survey/ Poster |
| Danese S., Sands B.E., Sandborn W.J., Marano C., O'Brien C., Zhang H., Johanns J., Peyrin-Biroulet L., Scherl E., Hisamatsu T., Panaccione R. | Efficacy of ustekinumab subcutaneous maintenance treatment by induction-dose subgroup in the unifi study of patients with ulcerative colitis | Review / Meta-Analysis / Survey/ Poster |
| Vermeire S., Krznarić Ž., Kobayashi T., Chen J., Agboton C., Kisfalvi K., Patel H., Sandborn W. | Effects of subcutaneous vedolizumab on healthrelated quality of life and work productivity in patients with ulcerative colitis: Results from the Phase 3 VISIBLE 1 trial | Review / Meta-Analysis / Survey/ Poster |
| Laoun R., Hofmann R. | UC trial designed more than 5 years ago in the light of the EMA guideline on the development of new medicinal products for the treatment of ulcerative colitis | Review / Meta-Analysis / Survey/ Poster |
| Rosario M., Polhamus D., Dirks N., Lock R., Yao X., Chen J., Chen C., Sun W., Feagan B., Sandborn W., D'Haens G. | Exposure-response relationship of vedolizumab subcutaneous treatment in patients with ulcerative colitis: VISIBLE 1 | Review / Meta-Analysis / Survey/ Poster |
| Vermeire S., Krznaric Z., Kobayashi T., Chen J., Agboton C., Kisfalvi K., Patel H., Sandborn W.J. | EFFECTS OF SUBCUTANEOUS VEDOLIZUMAB ON HEALTH-RELATED QUALITY OF LIFE AND WORK PRODUCTIVITY IN PATIENTS WITH ULCERATIVE COLITIS: RESULTS FROM THE PHASE 3 VISIBLE 1 TRIAL | Review / Meta-Analysis / Survey/ Poster |
| Sands B.E., Sandborn W.J., Panaccione R., O'Brien C., Zhang H., Johanns J., Zhou Y., Tikhonov I., Peyrin-Biroulet L., Van Assche G., Danese S., Targan S., Abreu M.T., Hisamatsu T., Scherl E., Leong R.W., Rowbotham D., Arasaradnam R.P., Marano C. | Efficacy and safety of ustekinumab for ulcerative colitis through 2 years: Unifi long-term extension | Unmet study design (not RCT, retrospective, prospective, observational, switching, case study) |
| Magro F, Lopes S, Silva M, Coelho R, Portela F, Branquinho D, Correia L, Fernandes S, Cravo M, Caldeira P, Sousa HT, Patita M, Lago P, Ramos J, Afonso J, Redondo I, Machado P, Cornillie F, Lopes J, Carneiro F; Portuguese IBD Group [GEDII]. | Low Golimumab Trough Levels at Week 6 Are Associated With Poor Clinical, Endoscopic and Histological Outcomes in Ulcerative Colitis Patients: Pharmacokinetic and Pharmacodynamic Sub-analysis of the Evolution Study | Unmet study design (not RCT, retrospective, prospective, observational, switching, case study) |
| Li K, Strauss R, Marano C, Greenbaum LE, Friedman JR, Peyrin-Biroulet L, Brodmerkel C, De Hertogh G. | A Simplified Definition of Histologic Improvement in Ulcerative Colitis and its Association With Disease Outcomes up to 30 Weeks from Initiation of Therapy: Post Hoc Analysis of Three Clinical Trials | I Unmet intervention (not ADA or VDZ or UST, biosimilar, combination) |
| Dickson I. | New trials in ulcerative colitis therapies | Review / Meta-Analysis / Survey/ Poster |
| Van Assche G., Targan S.R., Baker T., O'Brien C.D., Zhang H., Johanns J., Szapary P., Marano C., Rupert L.W., Rowbotham D., Hisamatsu T., Danese S., Sands B.E., Peyrin-Biroulet L. | Sustained remission in patients with moderate to severe ulcerative colitis: Results from the phase 3 UNIFI maintenance study | Review / Meta-Analysis / Survey/ Poster |
| Sandborn W.J., Strauss R., Zhang H., Johanns J., Szapary P., Marano C.W., Danese S. | CLINICAL REMISSION BY LEGACY VERSUS FDA DEFINITIONS: DEFINITION JUSTIFICATION AND RESULTS FROM UNIFI STUDY | Review / Meta-Analysis / Survey/ Poster |
| Sands B.E., Sandborn W.J., Panaccione R., O'Brien C., Zhang H., Johanns J., Peyrin-Biroulet L., Van Assche G., Danese S., Targan S., Abreu M.T., Hisamatsu T., Szapary P., Marano C.W. | Safety and efficacy of ustekinumab induction therapy in patients with moderate to severe ulcerative colitis: Results from the phase 3 unifi study | Review / Meta-Analysis / Survey/ Poster |
| Motoya S., Watanabe K., Ogata H., Kanai T., Matsui T., Suzuki Y., Shikamura M., Sugiura K., Oda K., Hori T., Araki T., Watanabe M., Hibi T. | A phase 3 study of vedolizumab in japanese patients with ulcerative colitis: Effects on time to disease worsening and treatment failure | Review / Meta-Analysis / Survey/ Poster |
| Arijs I., De Hertogh G., Lemmens B., Van Lommel L., De Bruyn M., Vanhove W., Cleynen I., MacHiels K., Ferrante M., Schuit F., Van Assche G., Rutgeerts P., Vermeire S. | Effect of vedolizumab (anti-α4β7-integrin) therapy on histological healing and mucosal gene expression in patients with UC | Review / Meta-Analysis / Survey/ Poster |
| Mshimesh B.A.R. | Efficacy and safety of adalimumab versus infliximab in patients suffered from moderate to severe active ulcerative colitis | Review / Meta-Analysis / Survey/ Poster |
| Neurath M.F. | Current and emerging therapeutic targets for IBD | Review / Meta-Analysis / Survey/ Poster |
| Abraham B.P., Ahmed T., Ali T. | Inflammatory bowel disease: Pathophysiology and current therapeutic approaches | Review / Meta-Analysis / Survey/ Poster |
| Yarur AJ, Jain A, Hauenstein SI, Quintero MA, Barkin JS, Deshpande AR, Sussman DA, Singh S, Abreu MT. | Higher Adalimumab Levels Are Associated with Histologic and Endoscopic Remission in Patients with Crohn's Disease and Ulcerative Colitis | Unmet study design (not RCT, retrospective, prospective, observational, switching, case study) |
| Magro F, Lopes SI, Lopes J, Portela F, Cotter J, Lopes S, Moreira MJ, Lago P, Peixe P, Albuquerque A, Rodrigues S, Silva MR, Monteiro P, Lopes C, Monteiro L, Macedo G, Veloso L, Camila C, Afonso J, Geboes K, Carneiro F; Portuguese IBD group [GEDII]. | Histological Outcomes and Predictive Value of Faecal Markers in Moderately to Severely Active Ulcerative Colitis Patients Receiving Infliximab | Unmet study design (not RCT, retrospective, prospective, observational, switching, case study) |
| Stallmach A., Schmidt C., Teich N. | Vedolizumab for the treatment of ulcerative colitis | Unmet intervention (not ADA or VDZ or UST, biosimilar, combination) |
| Reinisch W, Sandborn WJ, Panaccione R, Huang B, Pollack PF, Lazar A, Thakkar RB. | 52-week efficacy of adalimumab in patients with moderately to severely active ulcerative colitis who failed corticosteroids and/or immunosuppressants | Unmet study design (not RCT, retrospective, prospective, observational, switching, case study) |
| Villanacci V., Antonelli E., Geboes K., Casella G., Bassotti G. | Histological healing in inflammatory bowel disease: A still unfulfilled promise | Review / Meta-Analysis / Survey/ Poster |
