# Supplementary material for: An indirect comparison of efficacy including histologic assessment and safety in biologic therapy in ulcerative colitis: Systemic review and network meta-analysis
Source: PLoS One. 2023 Nov 2;18(11):e0293655. doi: 10.1371/journal.pone.0293655 (PMC10621919; doi:10.1371/journal.pone.0293655)
Supplement: S5 File — (DOCX) [file pone.0293655.s005.docx]

Definition of efficacy and safety outcome

| **Category** | **Endpoint** | **Definition** |
| --- | --- | --- |
| **Efficacy** | Histologic remission | Histologic remission is defined as Robarts histopathology index (RHI) of less than or equal to 3 point in UNIFI^17^ study and less than 3 point in VARSITY and VISIBLE1 studies^14,22^. The RHI is composed of 4 categories (1: lamina propria chronic inflammation; 2: lamina propria neutrophils; 3: epithelial neutrophils; 4: surface epithelial injury) rated from 1-4 that are summed with different weights to give a total score ranges from 0 to 33.^27^  Histologic remission is defined as Nancy histopathology index (NHI) of less than or equal to 1 point in in HIBISCUS1, 2 studies^26^. The NHI score ranges from 0 to 4, with the following definitions for each grade: 0 is no histologically significant disease; 1 is chronic inflammatory infiltrate with no acute inflammatory infiltrate; and 2, 3, and 4 are mildly, moderately, and severely active disease, respectively.^28^ |
|  | Clinical remission | Clinical remission is defined as total Mayo score of less than or equal to 2 with no individual subscore greater than 1. The Mayo score is composed of 4 categories (stool frequency, rectal bleeding, endoscopic appearance and physician’s global assessment) rated from 0 to 3 that are summed to give a total score that range from 0 to 12 and is the most commonly used activity index in clinical trials for UC.^29^ |
|  | Corticosteroid-free remission | Corticosteroid free remission is defined as clinical remission (defined as total Mayo score of less than or equal to 2 with no individual subscore greater than 1) without concomitant corticosteroids at the end of the maintenance phase in patients who were using corticosteroids at baseline. |
|  | Endoscopic improvement | Endoscopic improvement (previously termed as Mucosal healing) is defined as endoscopic subscore of Mayo score (MES) of less than or equal to 1 point. The MES evaluates the degree of endoscopic rectal inflammation based on a 4-point scale according to flexible proctosigmoidoscopy findings^29^ |
| **Safety** | TEAE | A TEAE is defined as any event not present before exposure to study drug or any event already present that worsens in either intensity or frequency after exposure to study drug, regardless of its causal relationship to study drug |
|  | TESAE | A TESAE is defined as any event considered serious by the investigator or that meet serious adverse event criteria of each clinical trial, not present before exposure to study drug or any event already present that worsens in either intensity or frequency after exposure to study drug, regardless of its causal relationship to study drug. |
|  | Infection | Infection is defined as an adverse event classified as ‘infections and infestations’ by system organ class of Medical Dictionary for Regulatory Activities (MedDRA), regardless of its causal relationship to study drug. |

Abbreviations: MES; endoscopic subscore of Mayo score, NHI; Nancy histopathology index, RHI; Robarts histopathology index, TEAE; treatment emergent adverse event, TESAE; treatment emergent serious adverse event
