# Supplementary material for: An indirect comparison of efficacy including histologic assessment and safety in biologic therapy in ulcerative colitis: Systemic review and network meta-analysis
Source: PLoS One. 2023 Nov 2;18(11):e0293655. doi: 10.1371/journal.pone.0293655 (PMC10621919; doi:10.1371/journal.pone.0293655)
Supplement: S6 File — (DOCX) [file pone.0293655.s006.docx]

Network Meta-analysis result

**Table 1.** Network Meta-analysis result of Histologic Remission

| **Induction** | |  | |
| --- | --- | --- | --- |
| **Network plot** | **Inconsistency** | **Network plot** | **Inconsistency** |
| **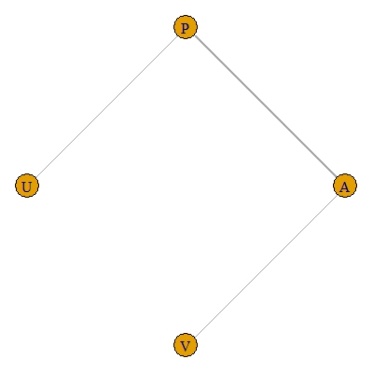** | **N/A** | **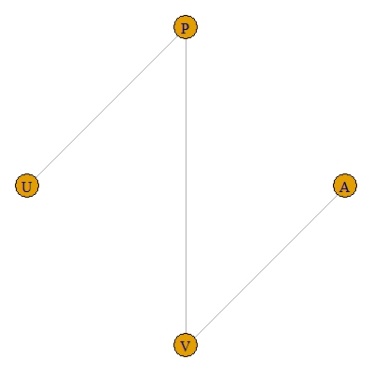** | **N/A** |
| **Forest plot (vs PBO)** | **Forest plot (ADA vs UST vs VDZ)** | **Forest plot (vs PBO)** | **Forest plot (ADA vs UST vs VDZ)** |
| **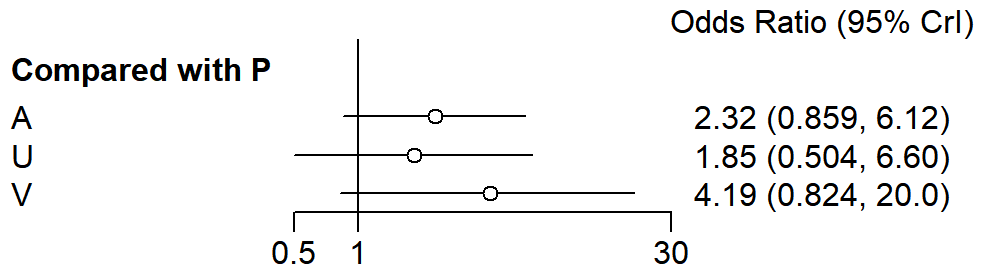** | **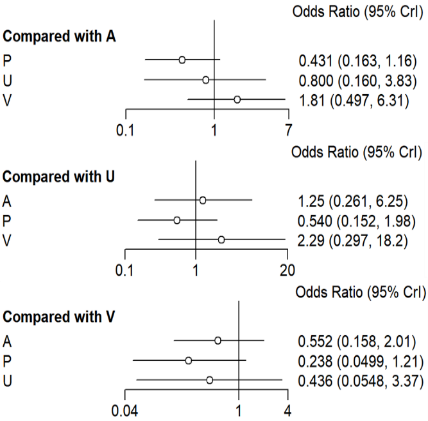** | **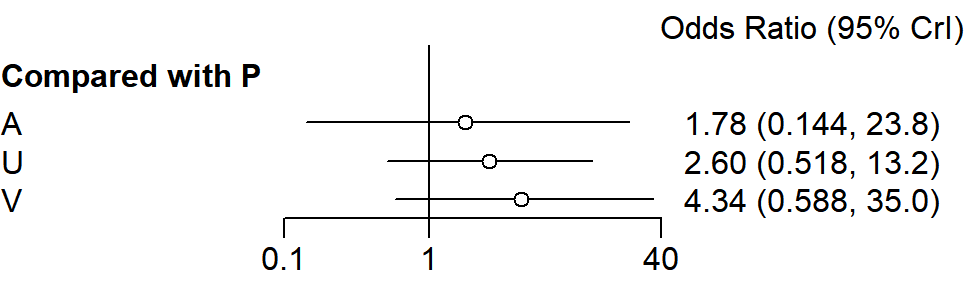** | **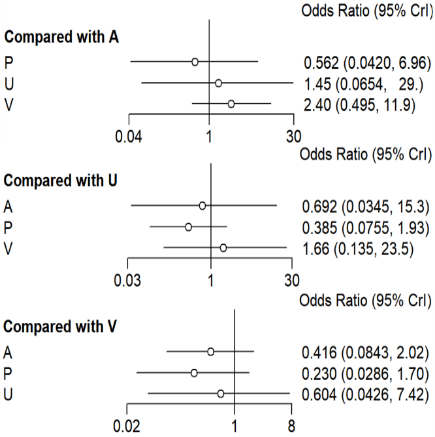** |
| **Rank Graph with Rank Probability** |  | **Rank Graph with Rank Probability** |  |
| **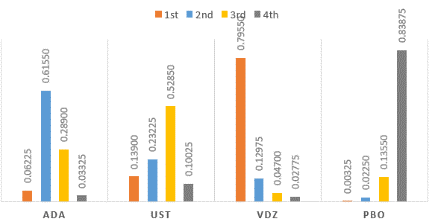** |  | **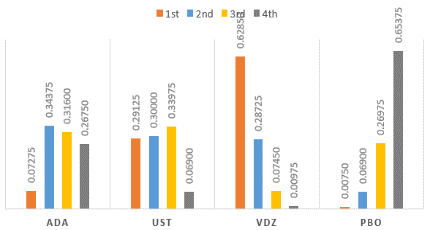** |  |

Table 2. Network Meta-analysis result of Clinical Remission

| **Induction** | |  | |
| --- | --- | --- | --- |
| **Network plot** | **Inconsistency** | **Network plot** | **Inconsistency** |
| **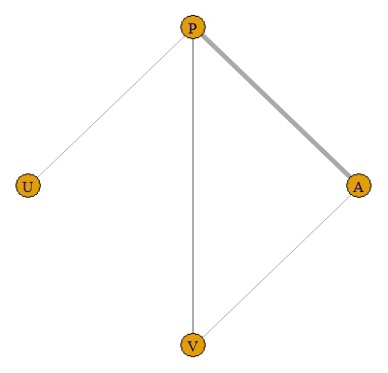** | **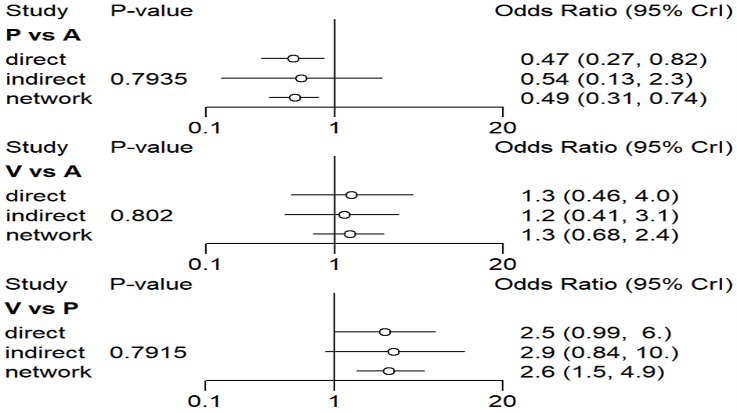** | **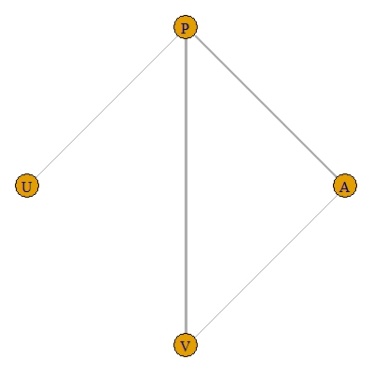** | **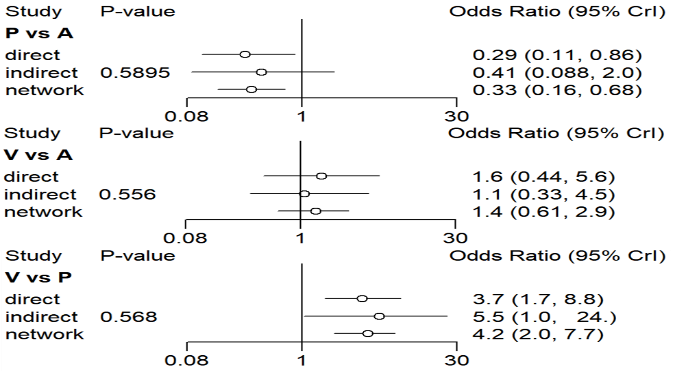** |
| **Forest plot (vs PBO)** | **Forest plot (ADA vs UST vs VDZ)** | **Forest plot (vs PBO)** | **Forest plot (ADA vs UST vs VDZ)** |
| **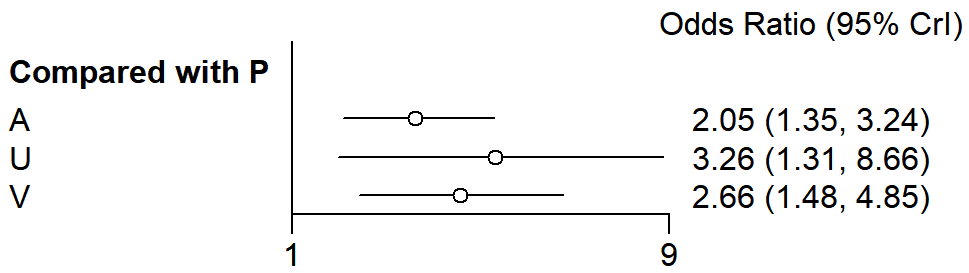** | **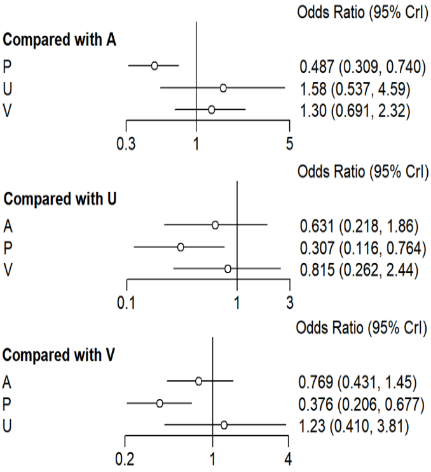** | **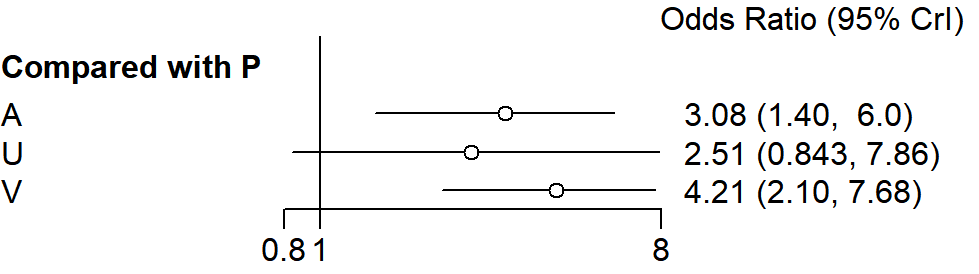** | **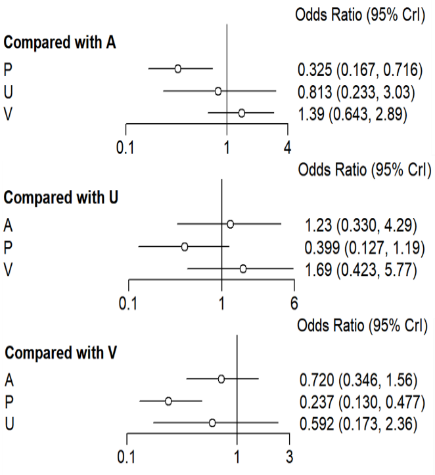** |
| **Rank Graph with Rank Probability** |  | **Rank Graph with Rank Probability** |  |
| **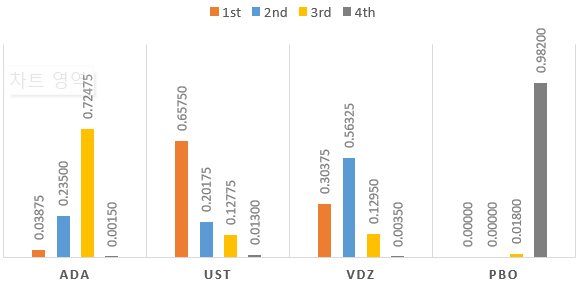** |  | **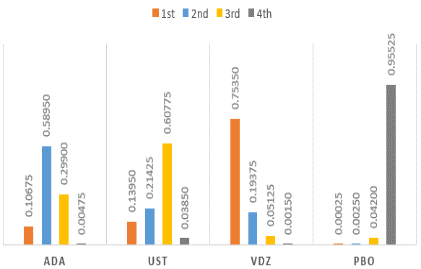** |  |

Table 3. Network Meta-analysis result of Corticosteroid-free Remission

| **Maintenance** | |
| --- | --- |
| **Network plot** | **Inconsistency** |
| **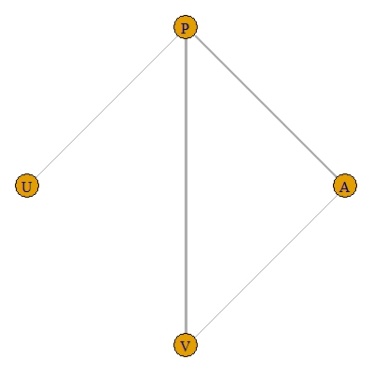** | **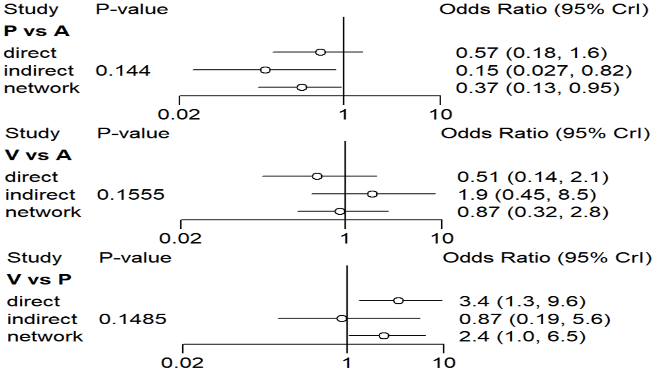** |
| **Forest plot (vs PBO)** | **Forest plot (ADA vs UST vs VDZ)** |
| **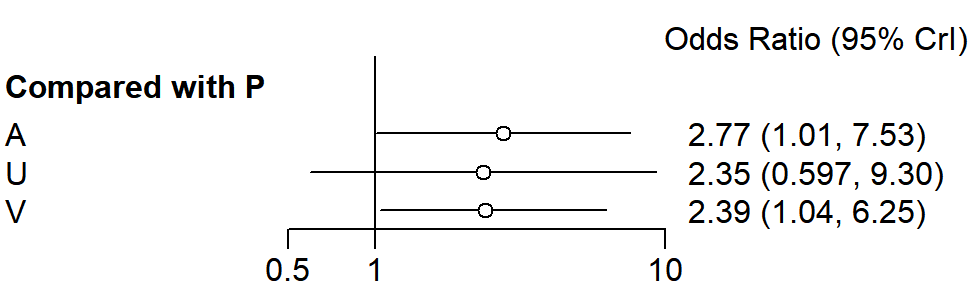** | **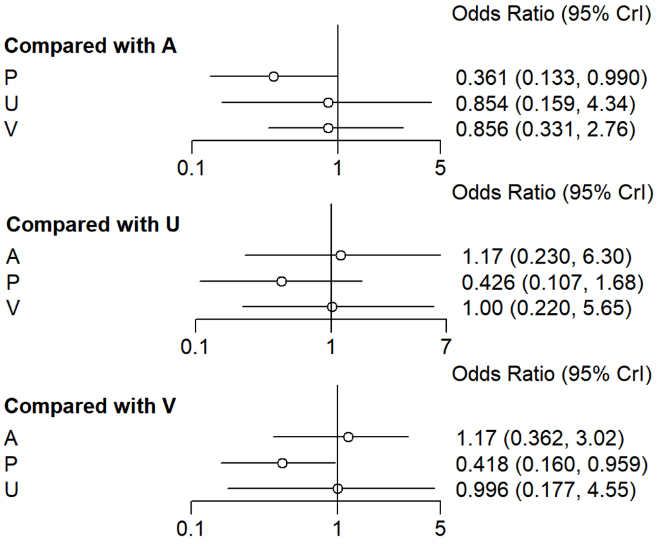** |
| **Rank Graph with Rank Probability** |  |
| **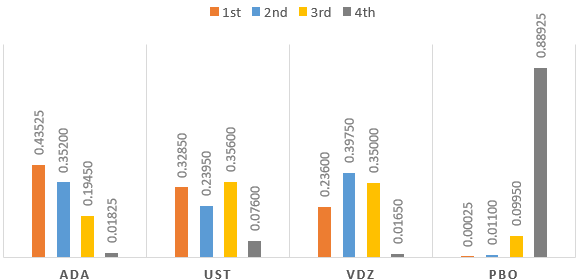** |  |

Table 4. Network Meta-analysis result of Endoscopic Improvement

| **Induction** | |  | |
| --- | --- | --- | --- |
| **Network plot** | **Inconsistency** | **Network plot** | **Inconsistency** |
| **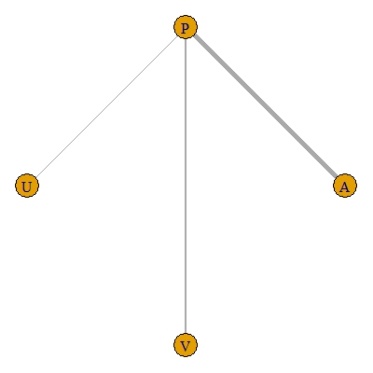** | **N/A** | **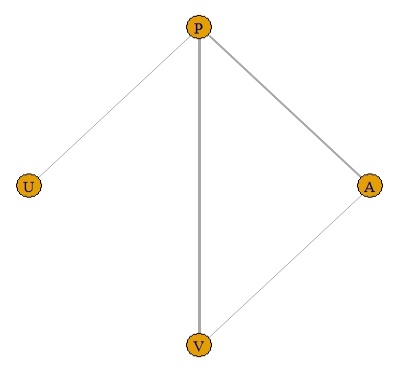** | **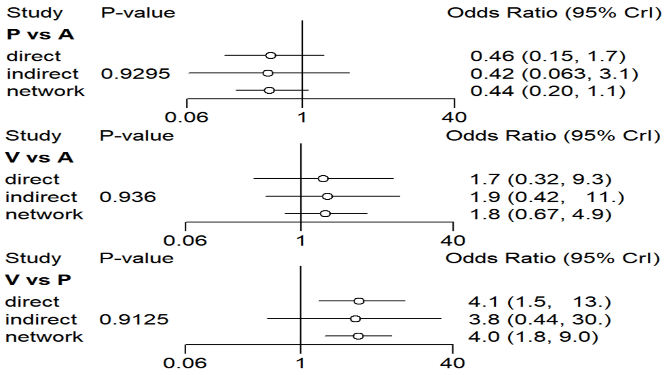** |
| **Forest plot (vs PBO)** | **Forest plot (ADA vs UST vs VDZ)** | **Forest plot (vs PBO)** | **Forest plot (ADA vs UST vs VDZ)** |
| **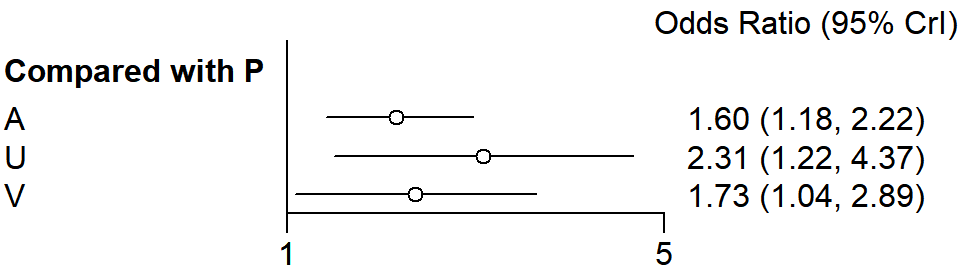** | **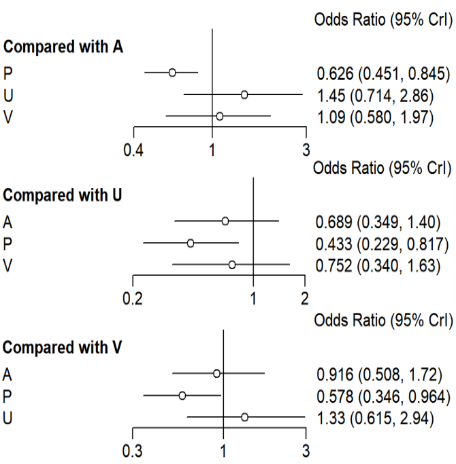** | **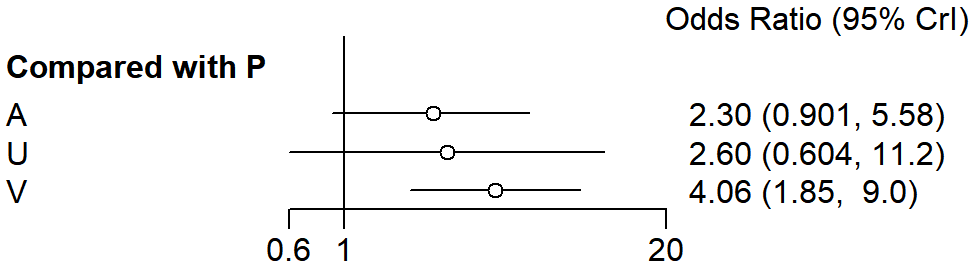** | **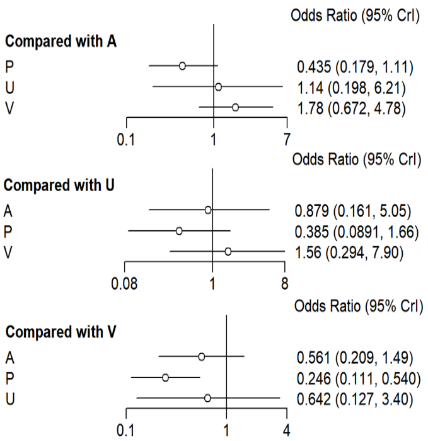** |
| **Rank Graph with Rank Probability** |  | **Rank Graph with Rank Probability** |  |
| **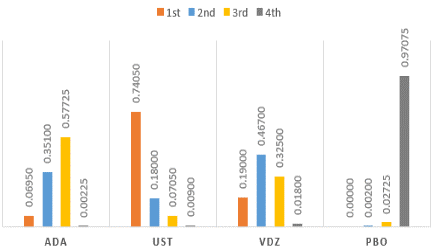** |  | **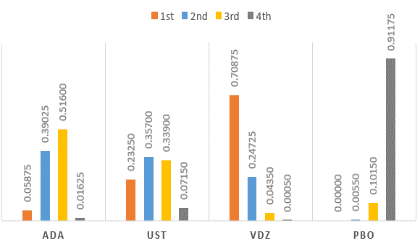** |  |

Table 5. Network Meta-analysis result of TEAE

| **Induction** | |  | |
| --- | --- | --- | --- |
| **Network plot** | **Inconsistency** | **Network plot** | **Inconsistency** |
| **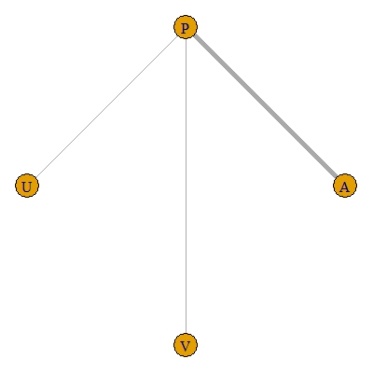** | **N/A** | **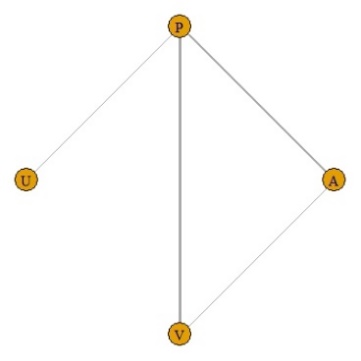** | **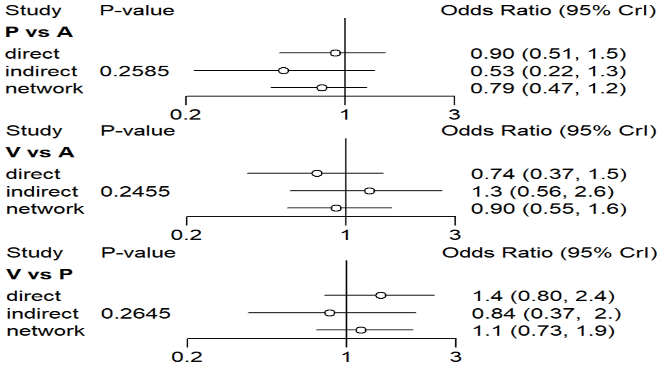** |
| **Forest plot (vs PBO)** | **Forest plot (ADA vs UST vs VDZ)** | **Forest plot (vs PBO)** | **Forest plot (ADA vs UST vs VDZ)** |
| **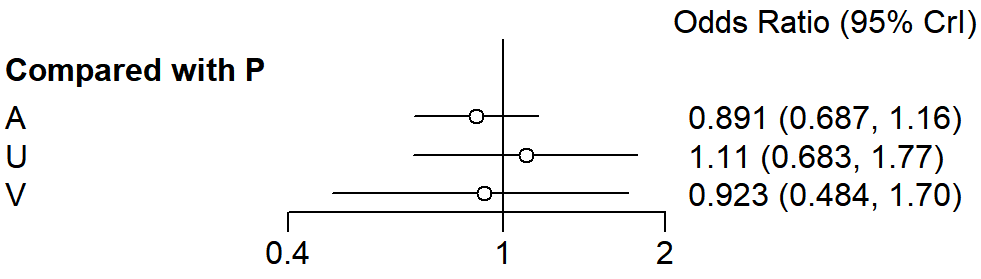** | **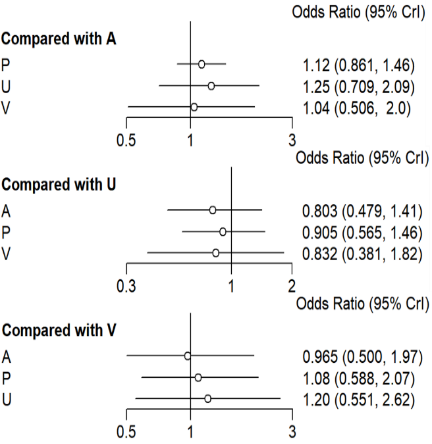** | **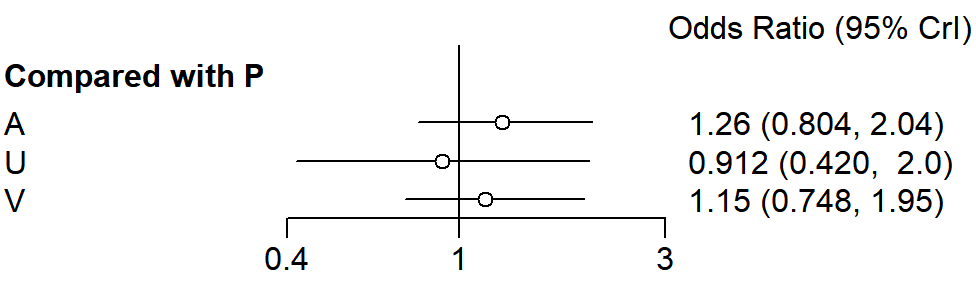** | **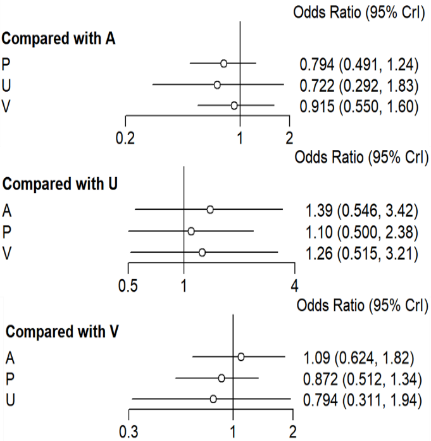** |
| **Rank Graph with Rank Probability** |  | **Rank Graph with Rank Probability** |  |
| **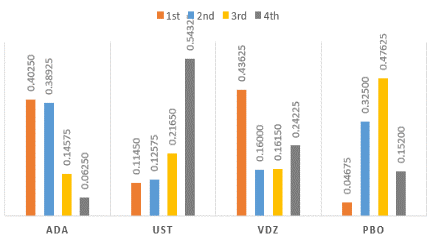** |  | **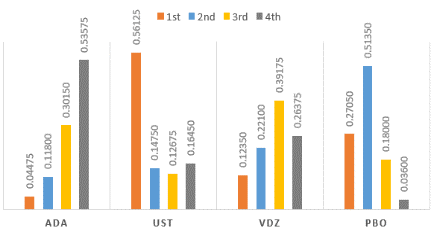** |  |

Table 6. Network Meta-analysis result of TESAE

| **Induction** | |  | |
| --- | --- | --- | --- |
| **Network plot** | **Inconsistency** | **Network plot** | **Inconsistency** |
| **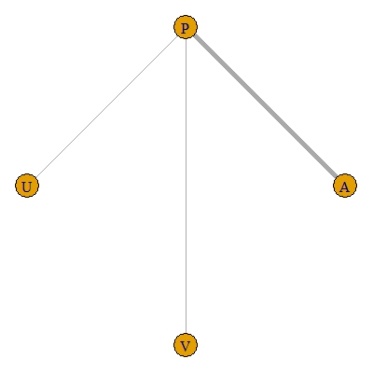** | **N/A** | **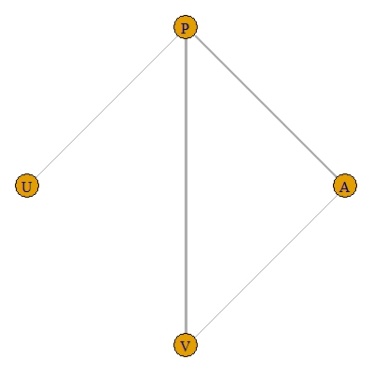** | **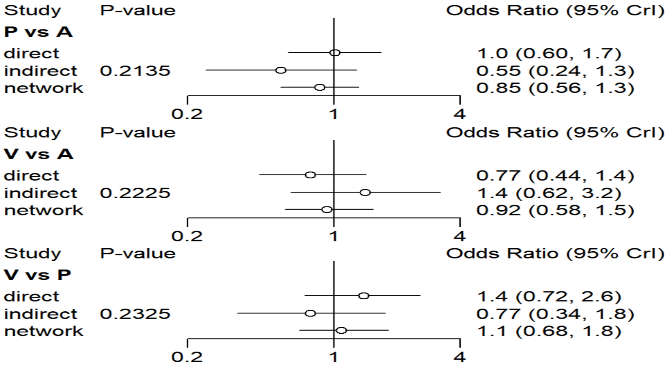** |
| **Forest plot (vs PBO)** | **Forest plot (ADA vs UST vs VDZ)** | **Forest plot (vs PBO)** | **Forest plot (ADA vs UST vs VDZ)** |
| **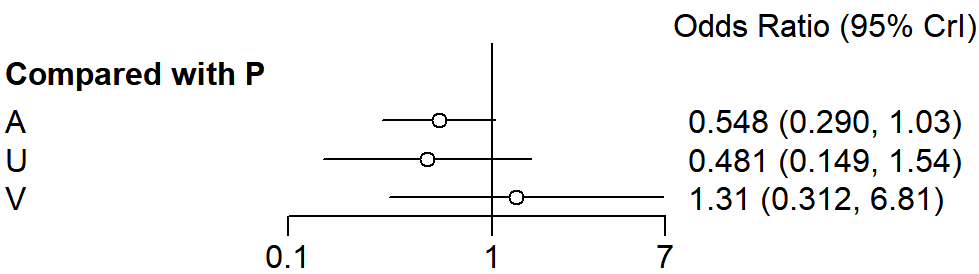** | **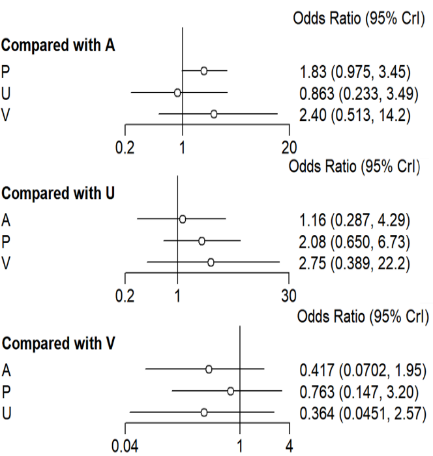** | **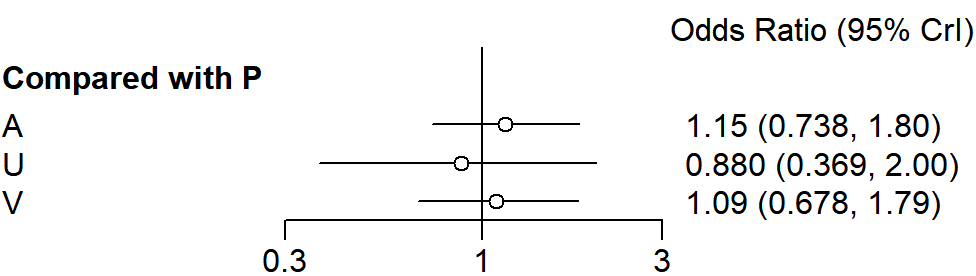** | **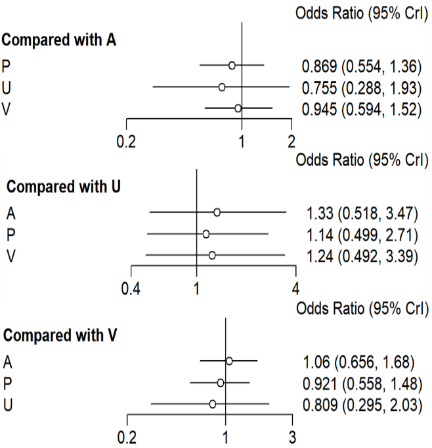** |
| **Rank Graph with Rank Probability** |  | **Rank Graph with Rank Probability** |  |
| **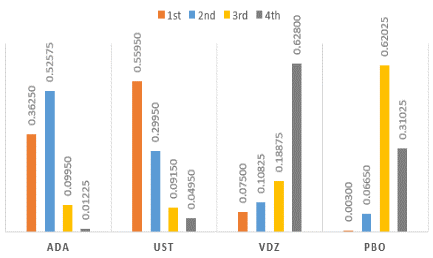** |  | **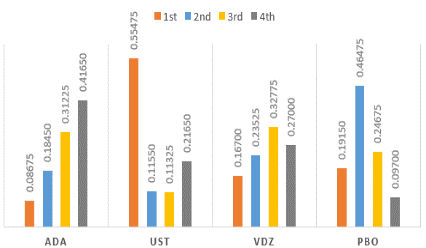** |  |

Table 7. Network Meta-analysis result of Infection

| **Induction** | |  | |
| --- | --- | --- | --- |
| **Network plot** | **Inconsistency** | **Network plot** | **Inconsistency** |
| **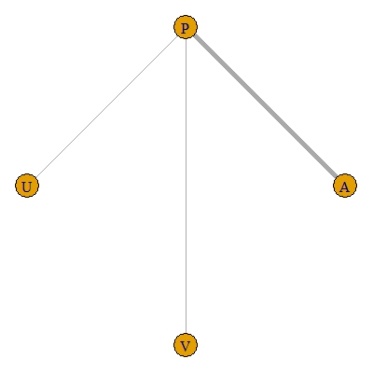** | **N/A** | **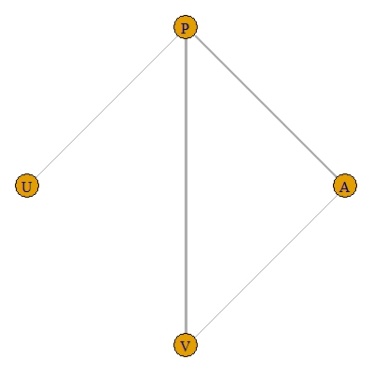** | **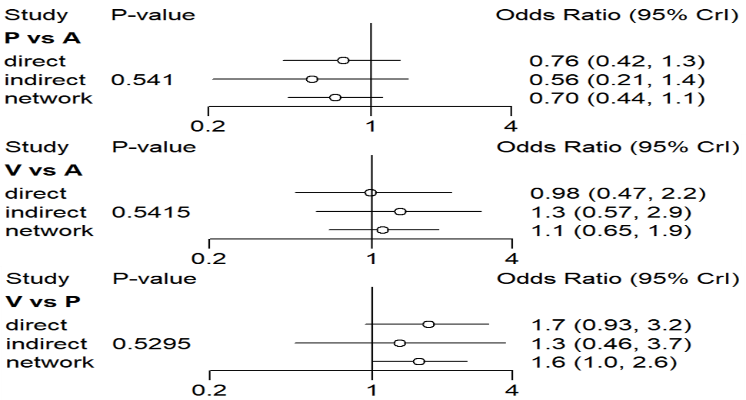** |
| **Forest plot (vs PBO)** | **Forest plot (ADA vs UST vs VDZ)** | **Forest plot (vs PBO)** | **Forest plot (ADA vs UST vs VDZ)** |
| **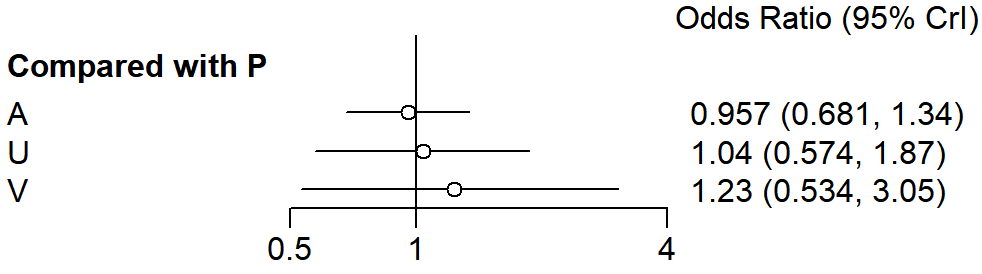** | **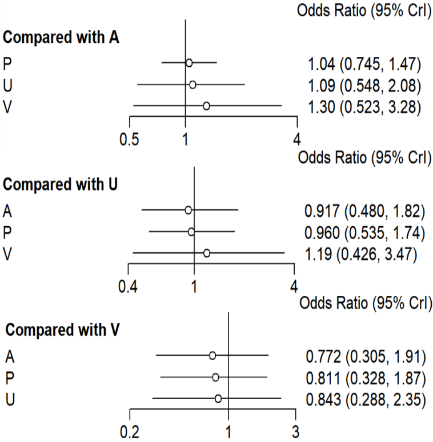** | **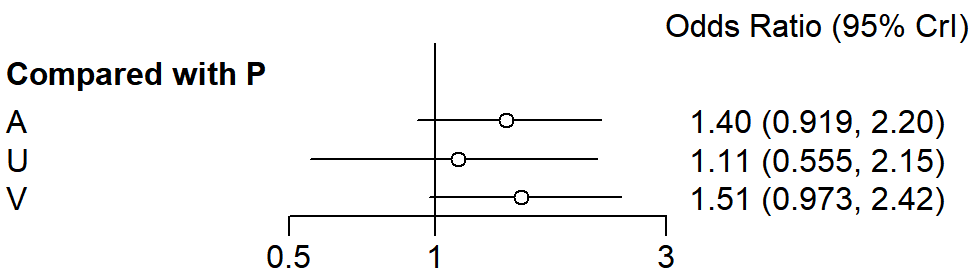** | **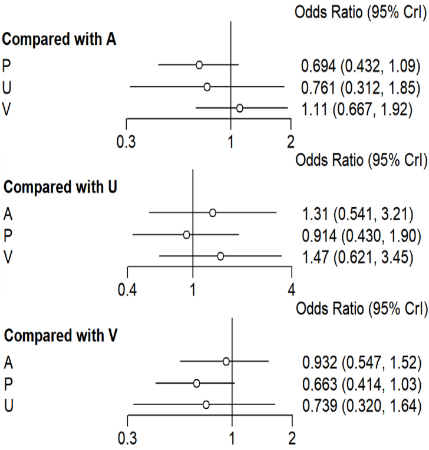** |
| **Rank Graph with Rank Probability** |  | **Rank Graph with Rank Probability** |  |
| **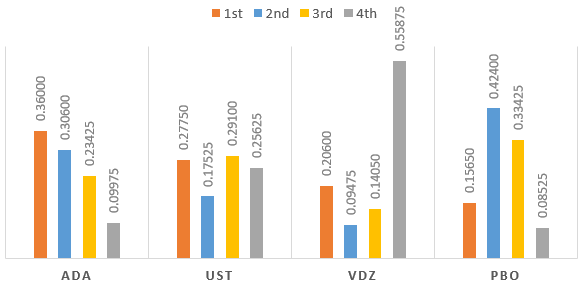** |  | **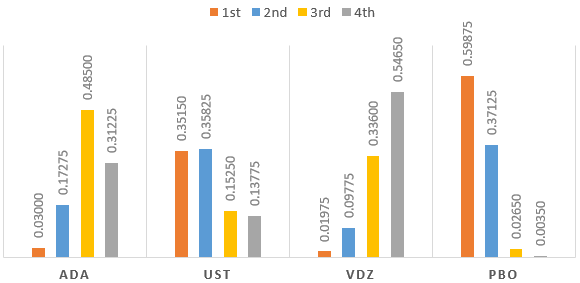** |  |
