# Supplementary figures and images for: An indirect comparison of efficacy including histologic assessment and safety in biologic therapy in ulcerative colitis: Systemic review and network meta-analysis
Source: PLoS One. 2023 Nov 2;18(11):e0293655. doi: 10.1371/journal.pone.0293655 (PMC10621919; doi:10.1371/journal.pone.0293655)

Risk of Bias 2 assessment


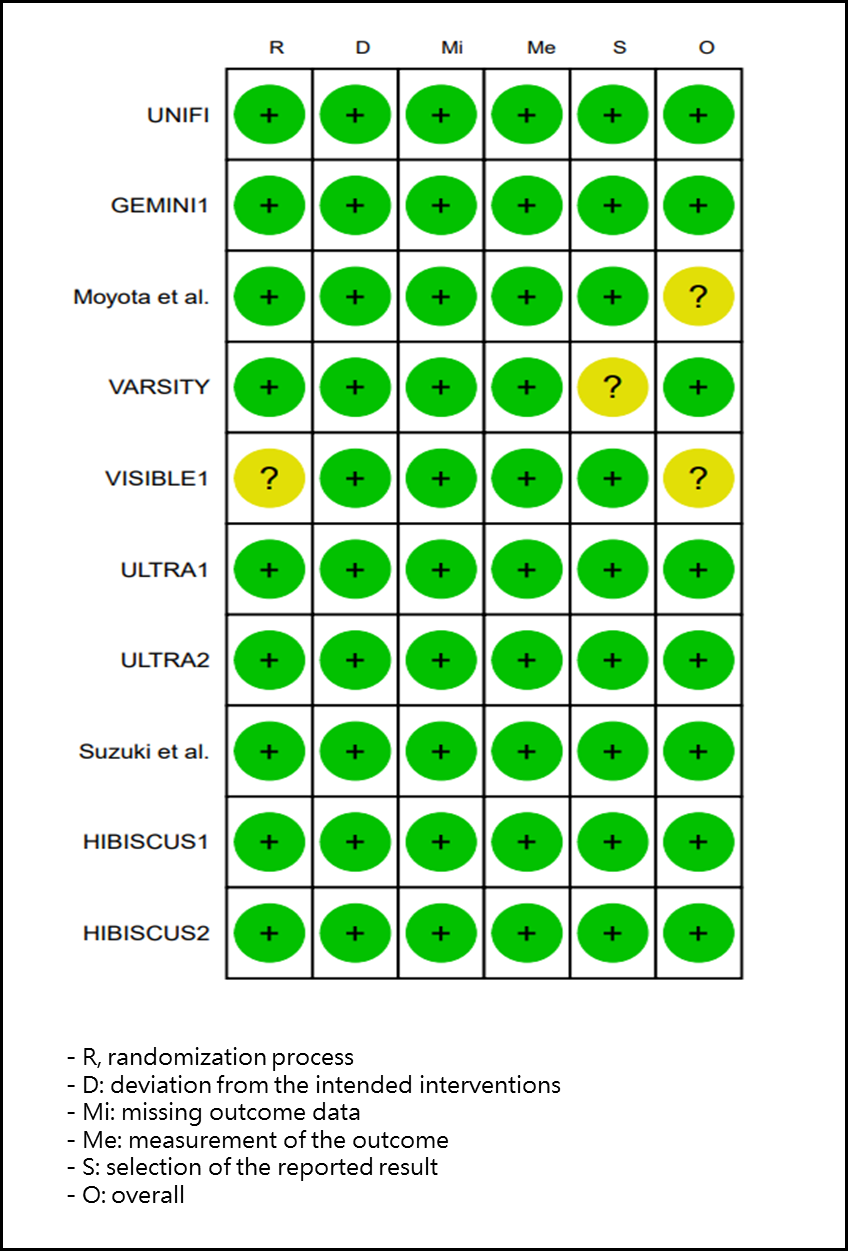

Supplement: S7 File — (DOCX) [file pone.0293655.s007.docx]
