# Supplementary material for: An indirect comparison of efficacy including histologic assessment and safety in biologic therapy in ulcerative colitis: Systemic review and network meta-analysis
Source: PLoS One. 2023 Nov 2;18(11):e0293655. doi: 10.1371/journal.pone.0293655 (PMC10621919; doi:10.1371/journal.pone.0293655)
Supplement: S8 File — (DOCX) [file pone.0293655.s008.docx]

Result of Sensitivity analysis

| **Clinical Remission (Induction) – excluding Motoya et al. 2019** | |
| --- | --- |
| **Network plot** | **Inconsistency** |
| **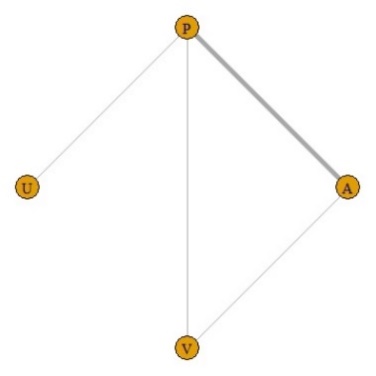** | **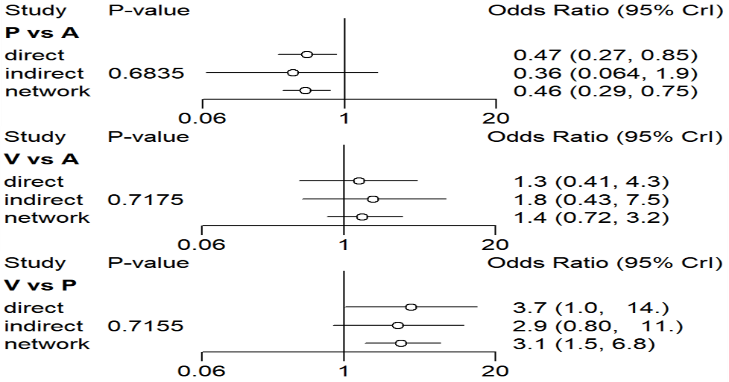** |
| **Forest plot (vs PBO)** | **Forest plot (ADA vs UST vs VDZ)** |
| **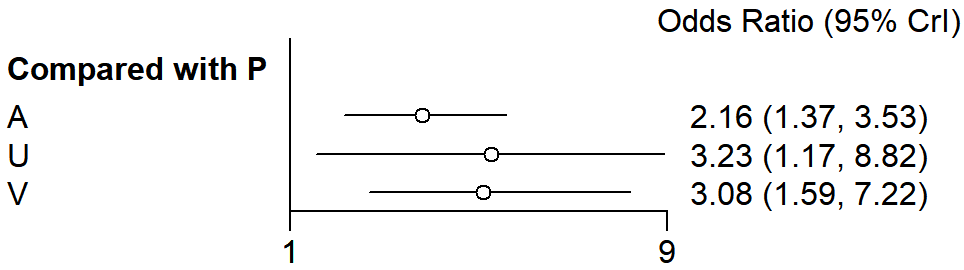** | **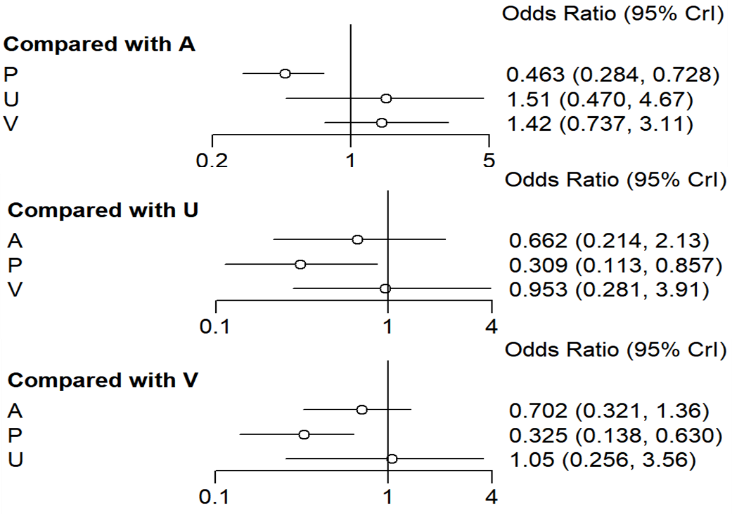** |
| **Rank Graph with Rank Probability** |  |
| **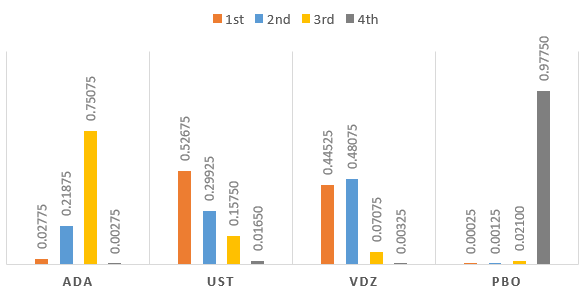** |  |
| **Clinical Remission (Induction) – excluding Suzuki et al. 2014** | |
| **Network plot** | **Inconsistency** |
| **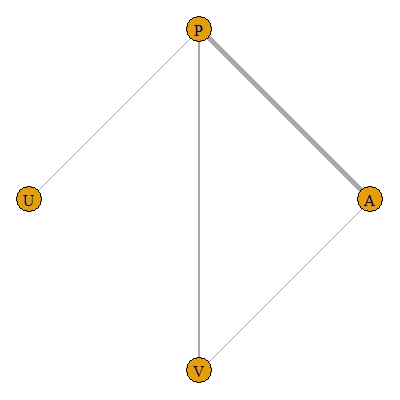** | **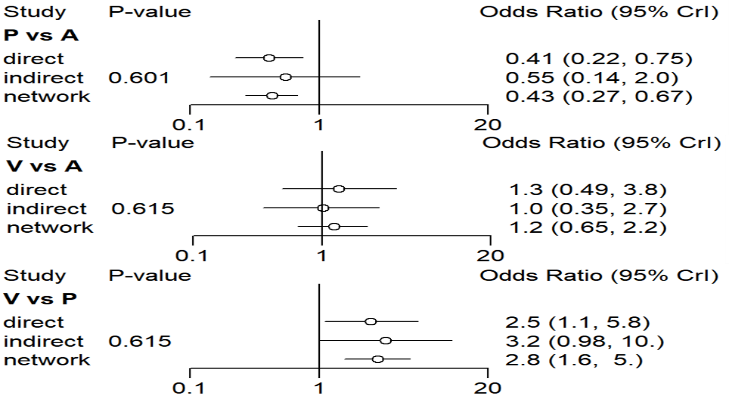** |
| **Forest plot (vs PBO)** | **Forest plot (ADA vs UST vs VDZ)** |
| **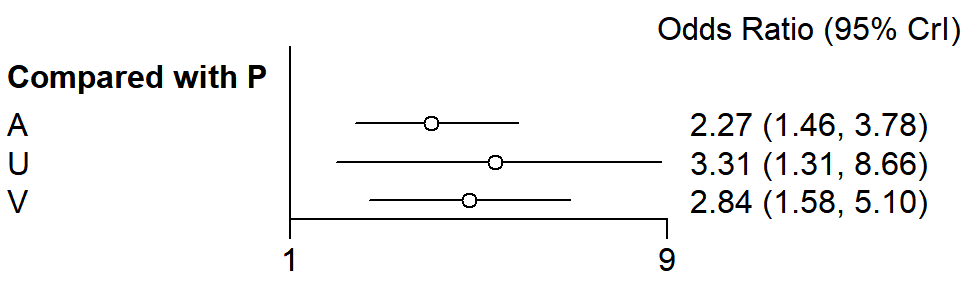** | **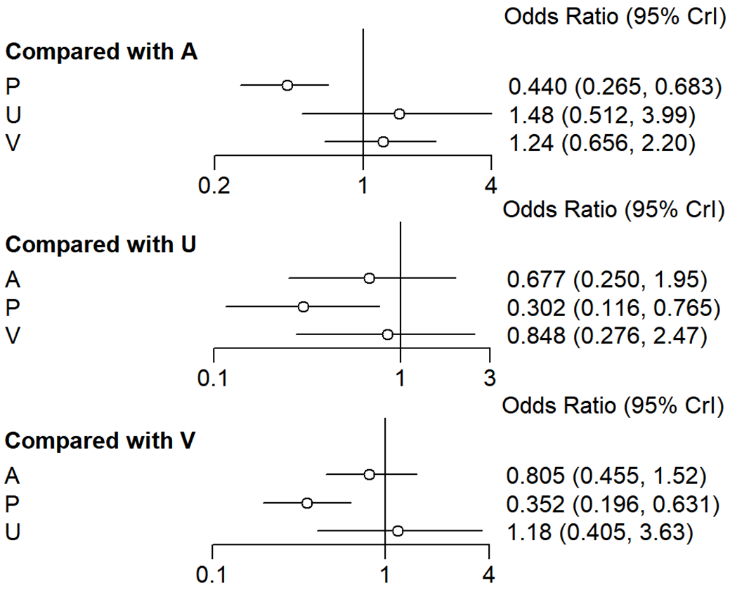** |
| **Rank Graph with Rank Probability** |  |
| **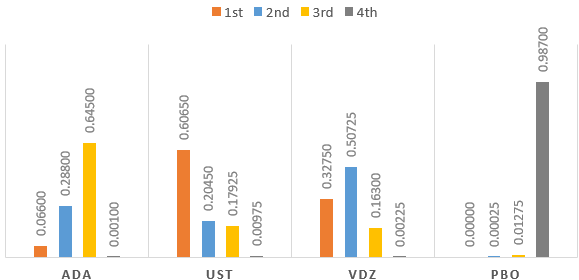** |  |
| **Clinical Remission (Induction) – excluding Motoya et al. 2019 and Suzuki et al. 2014** | |
| **Network plot** | **Inconsistency** |
| **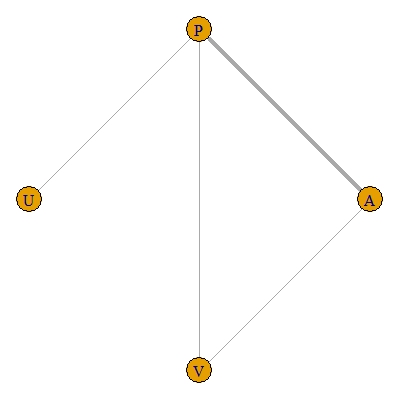** | **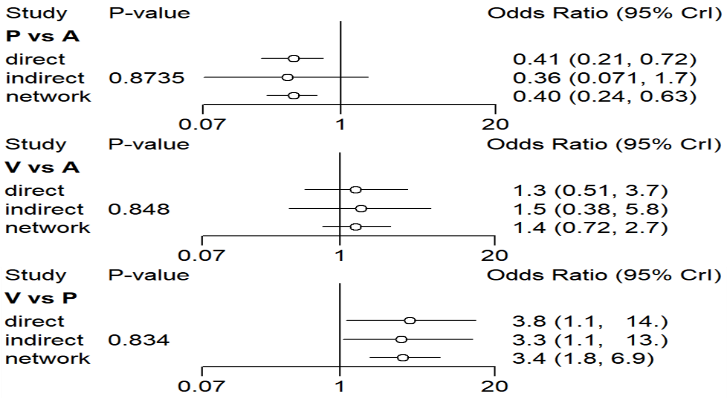** |
| **Forest plot (vs PBO)** | **Forest plot (ADA vs UST vs VDZ)** |
| **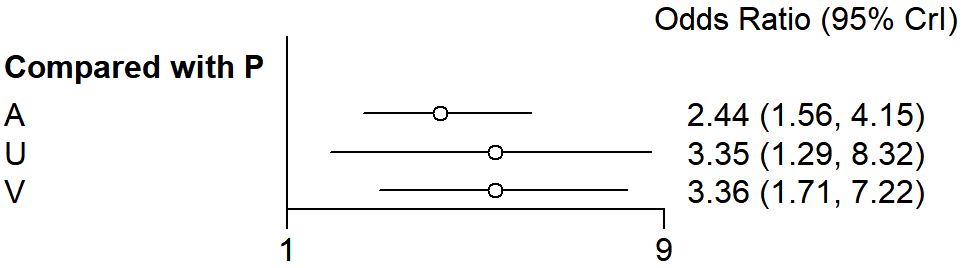** | **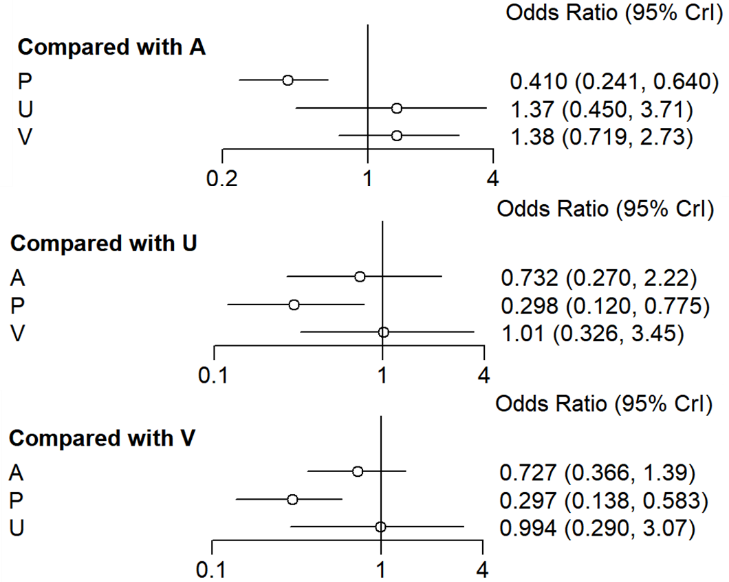** |
| **Rank Graph with Rank Probability** |  |
| **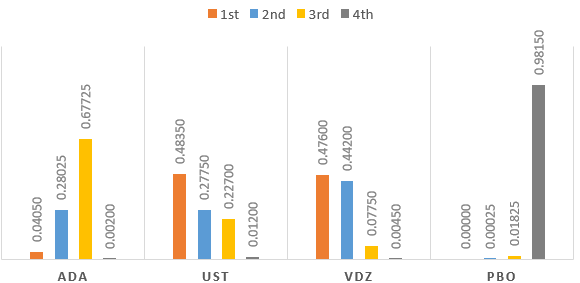** |  |
| **Clinical Remission (Maintenance) – excluding Motoya et al. 2019** | |
| **Network plot** | **Inconsistency** |
| **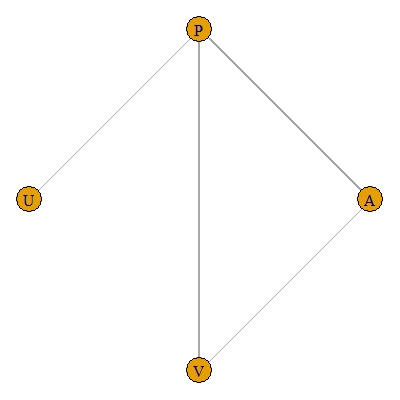** | **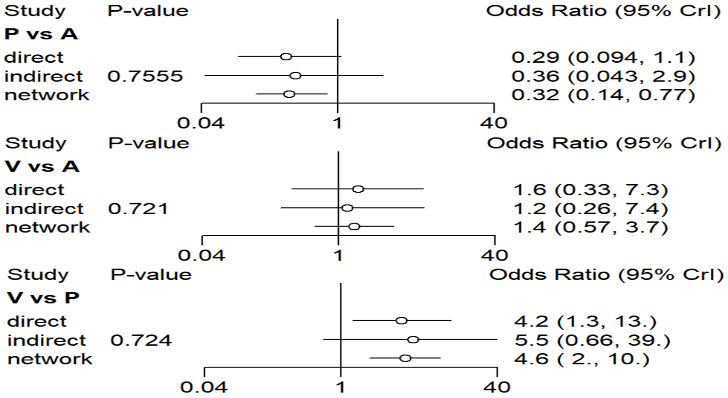** |
| **Forest plot (vs PBO)** | **Forest plot (ADA vs UST vs VDZ)** |
| **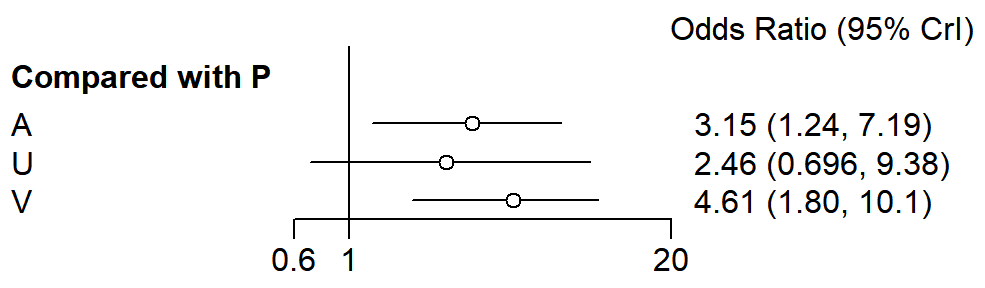** | **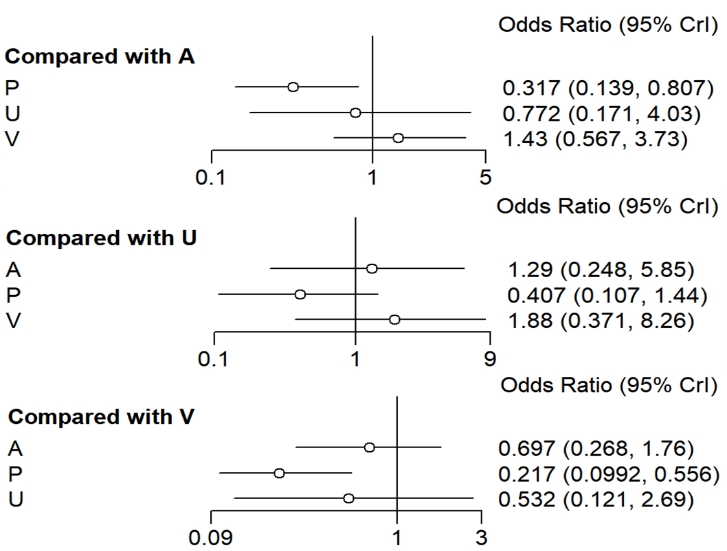** |
| **Rank Graph with Rank Probability** |  |
| **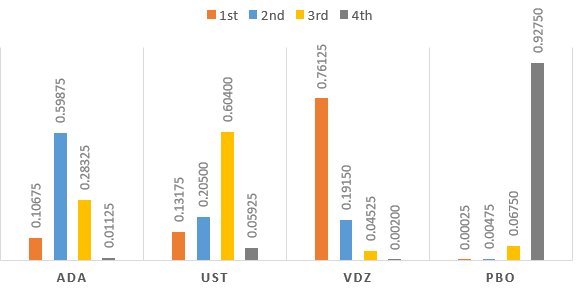** |  |
| **Clinical Remission (Maintenance) – excluding Suzuki et al. 2014** | |
| **Network plot** | **Inconsistency** |
| **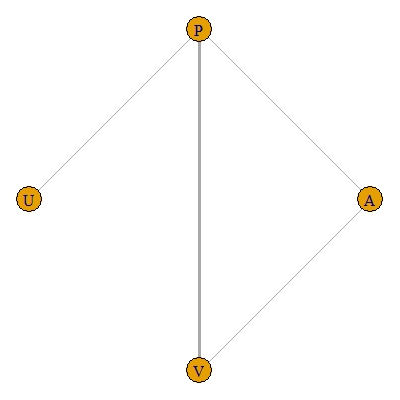** | **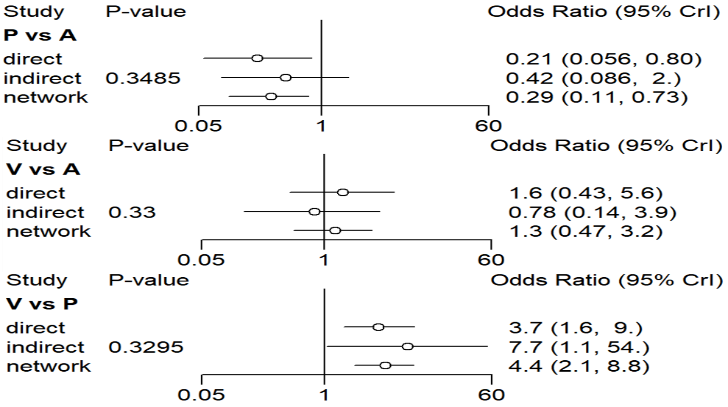** |
| **Forest plot (vs PBO)** | **Forest plot (ADA vs UST vs VDZ)** |
| **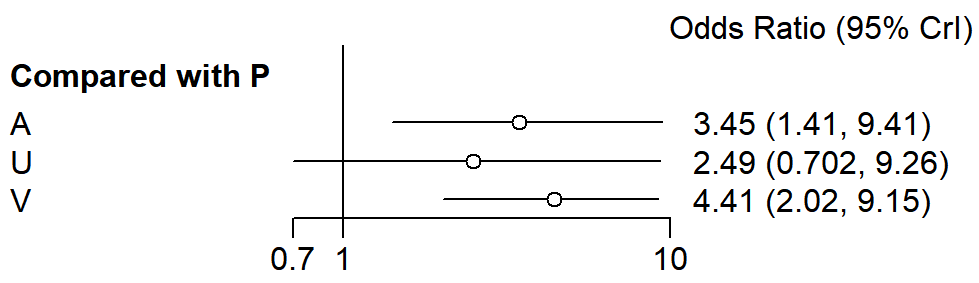** | **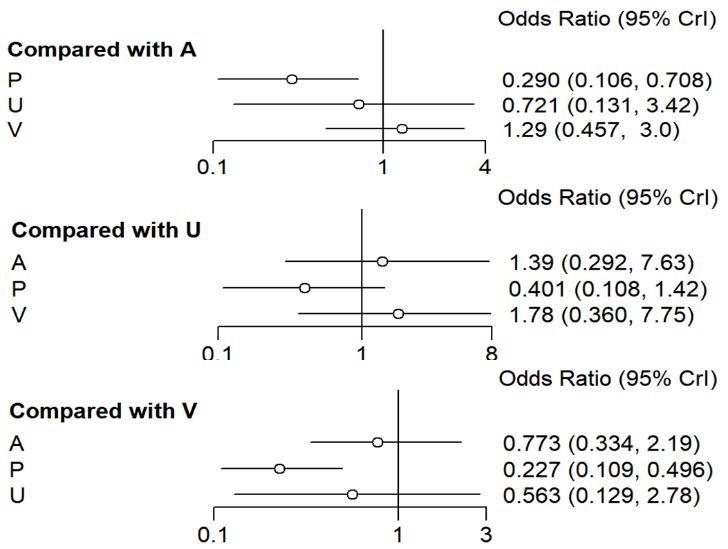** |
| **Rank Graph with Rank Probability** |  |
| **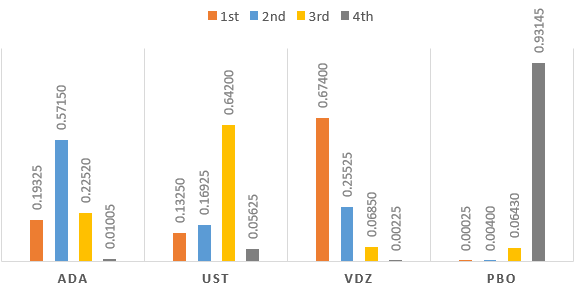** |  |
| **Clinical Remission (Maintenance) – excluding Motoya et al. 2019 and Suzuki et al. 2014** | |
| **Network plot** | **Inconsistency** |
| **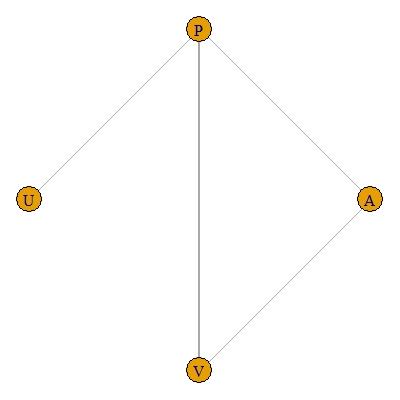** | **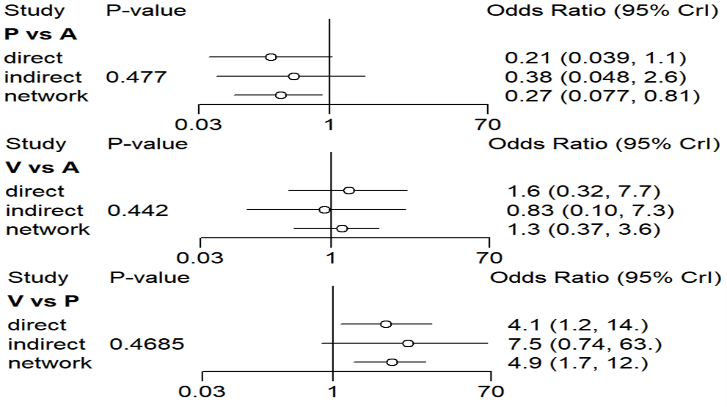** |
| **Forest plot (vs PBO)** | **Forest plot (ADA vs UST vs VDZ)** |
| **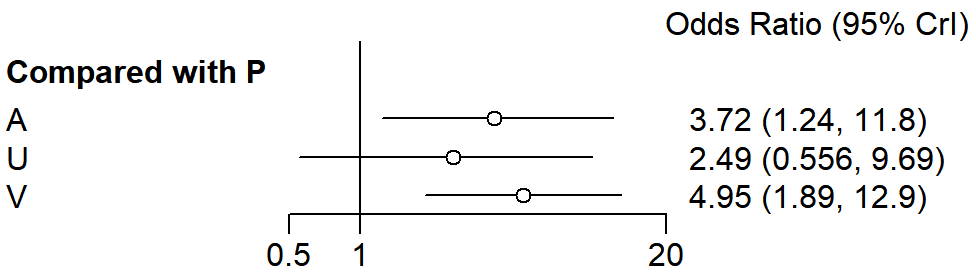** | **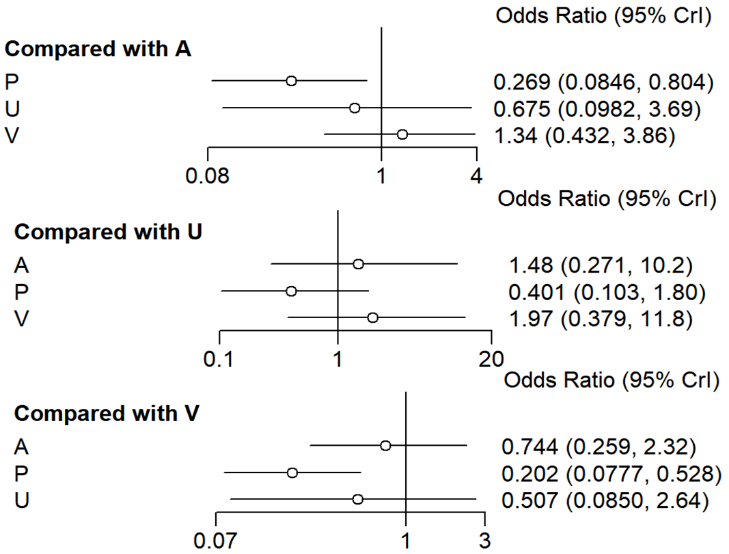** |
| **Rank Graph with Rank Probability** |  |
| **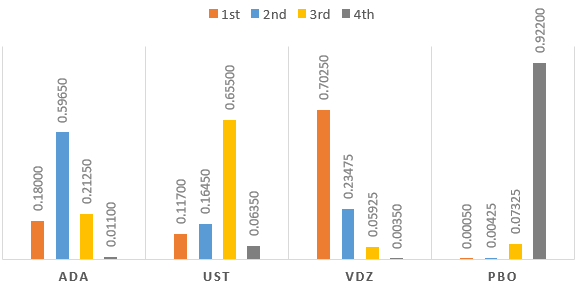** |  |
| **Corticosteroid-free Remission (Maintenance) – excluding Motoya et al. 2019** | |
| **Network plot** | **Inconsistency** |
| **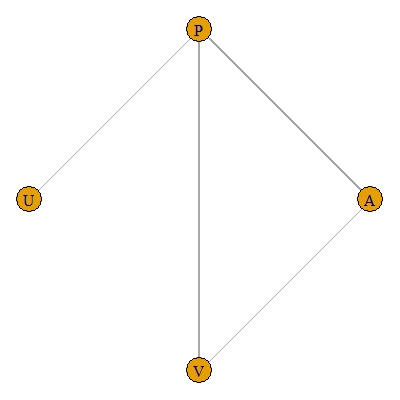** | **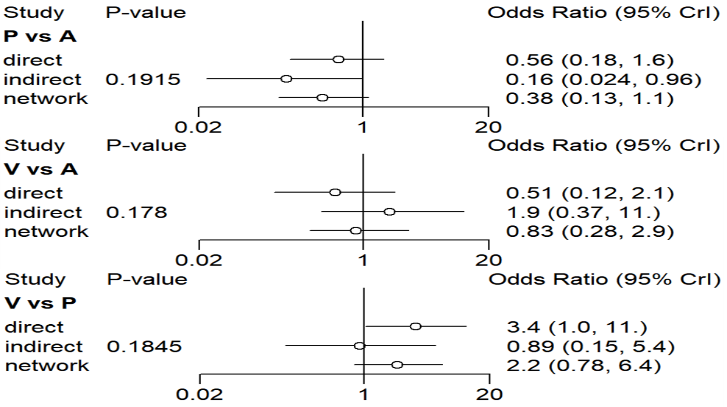** |
| **Forest plot (vs PBO)** | **Forest plot (ADA vs UST vs VDZ)** |
| **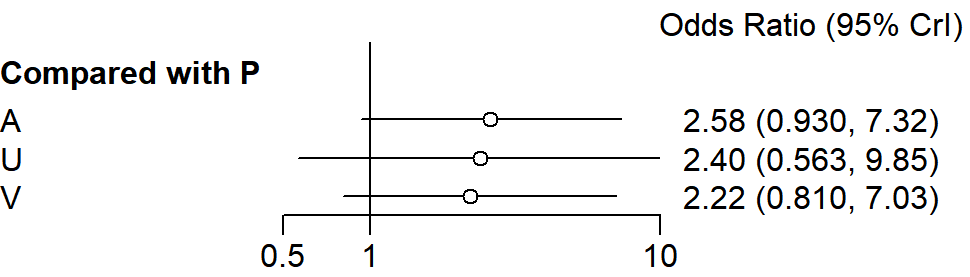** | **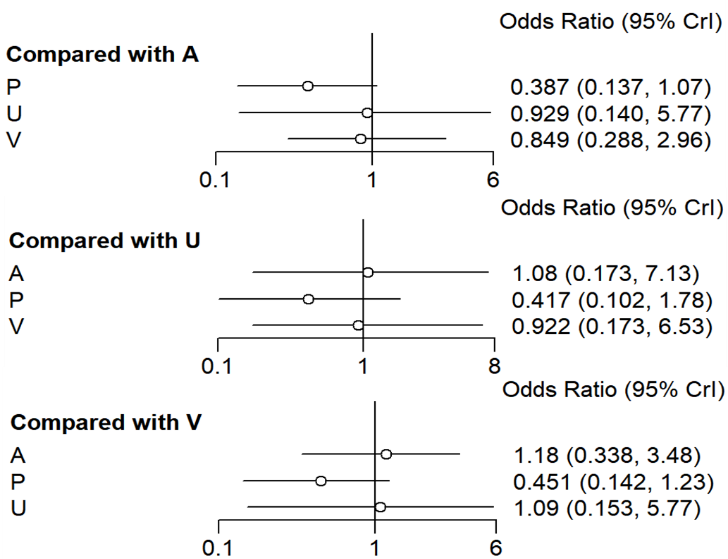** |
| **Rank Graph with Rank Probability** |  |
| **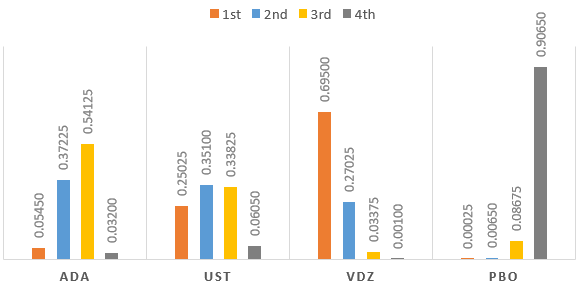** |  |
| **Corticosteroid-free Remission (Maintenance) – excluding Suzuki et al. 2014** | |
| **Network plot** | **Inconsistency** |
| **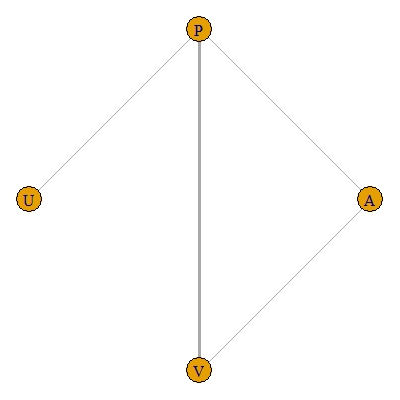** | **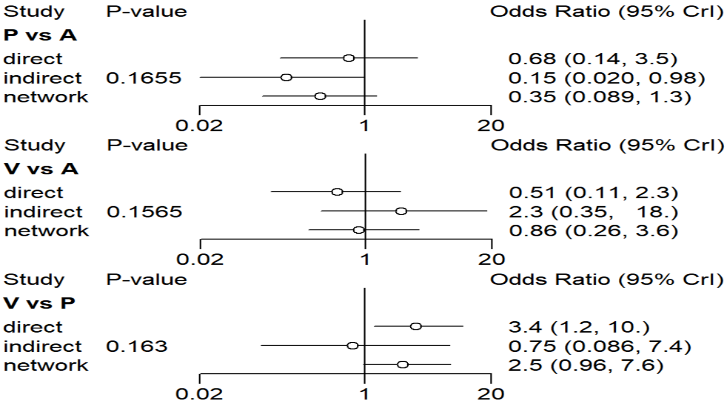** |
| **Forest plot (vs PBO)** | **Forest plot (ADA vs UST vs VDZ)** |
| **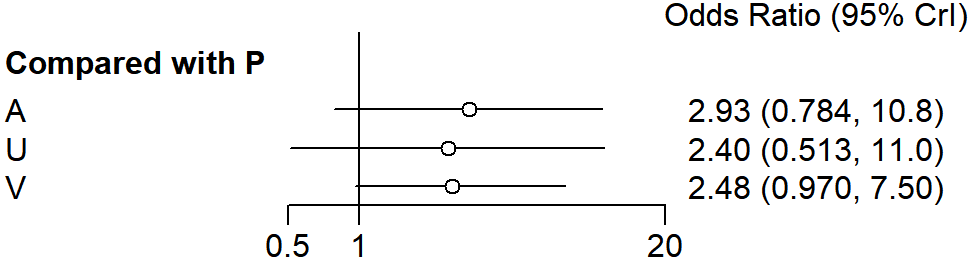** | **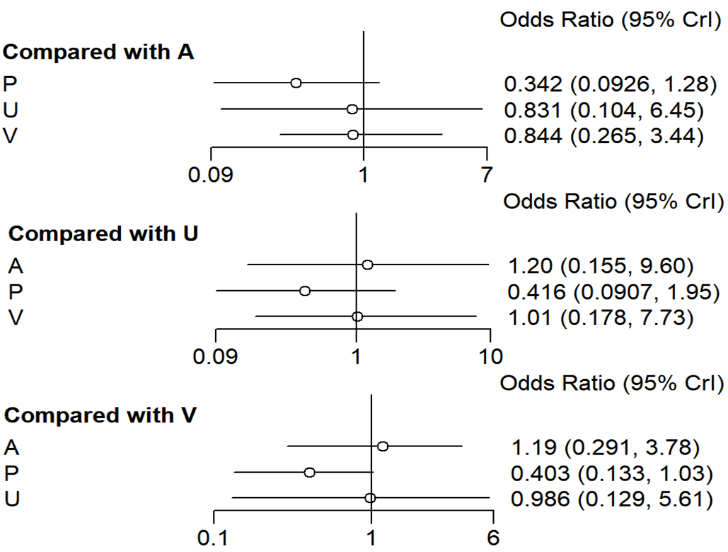** |
| **Rank Graph with Rank Probability** |  |
| **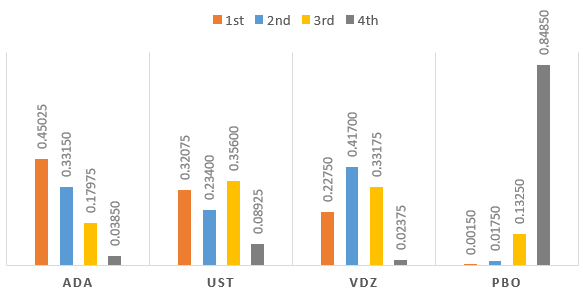** |  |
| **Corticosteroid-free Remission (Maintenance) – excluding Motoya et al. 2019 and Suzuki et al. 2014** | |
| **Network plot** | **Inconsistency** |
| **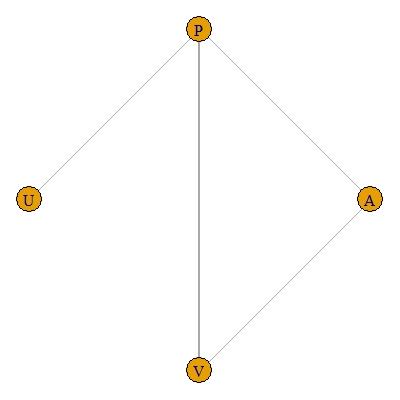** | **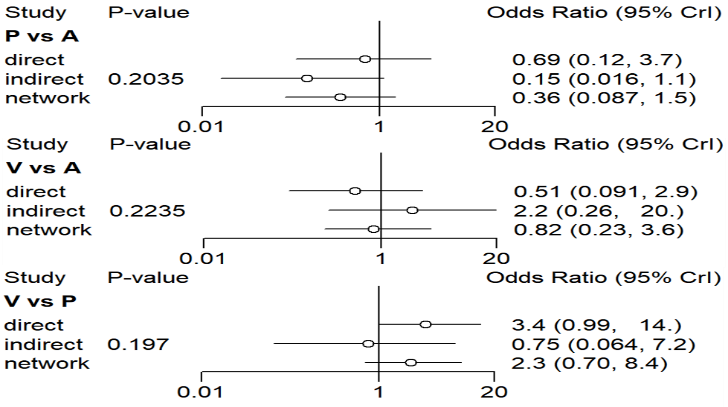** |
| **Forest plot (vs PBO)** | **Forest plot (ADA vs UST vs VDZ)** |
| **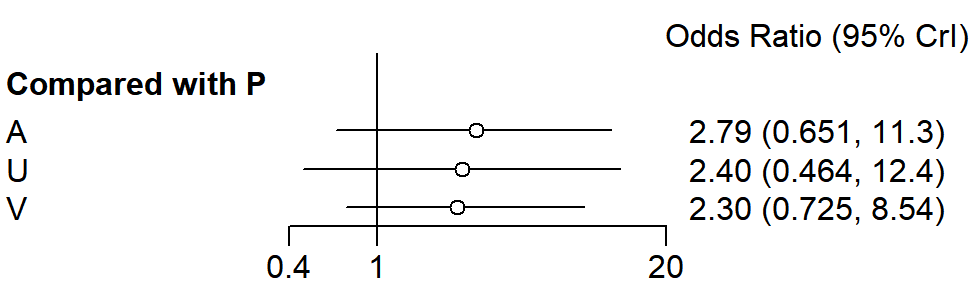** | **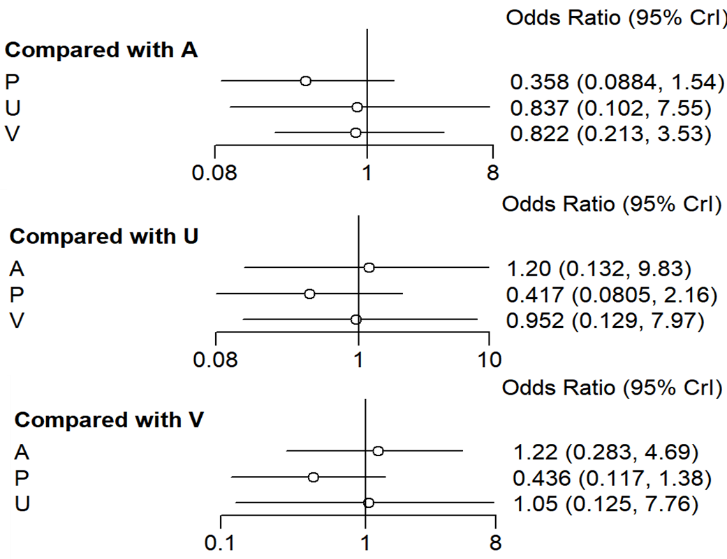** |
| **Rank Graph with Rank Probability** |  |
| **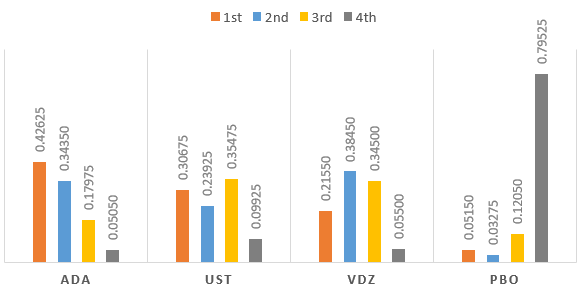** |  |
| **Endoscopic Improvement (Induction) – excluding Motoya et al. 2019** | |
| **Network plot** | **Inconsistency** |
| **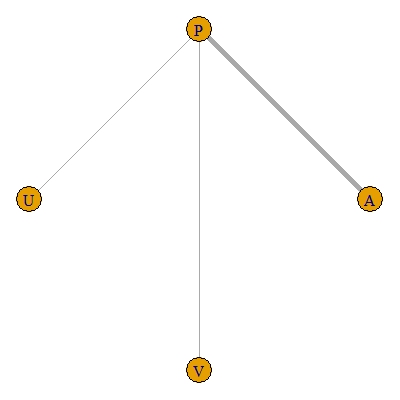** | **N/A** |
| **Forest plot (vs PBO)** | **Forest plot (ADA vs UST vs VDZ)** |
| **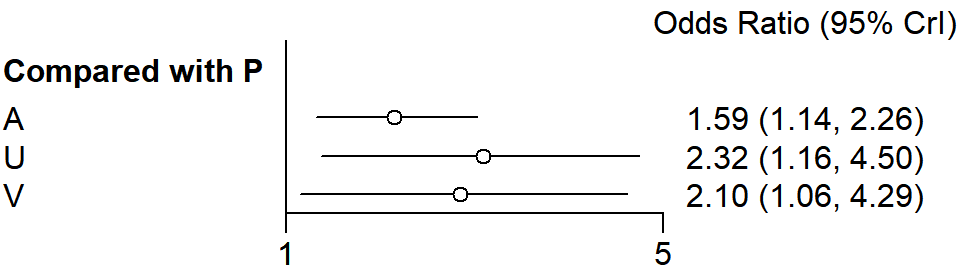** | **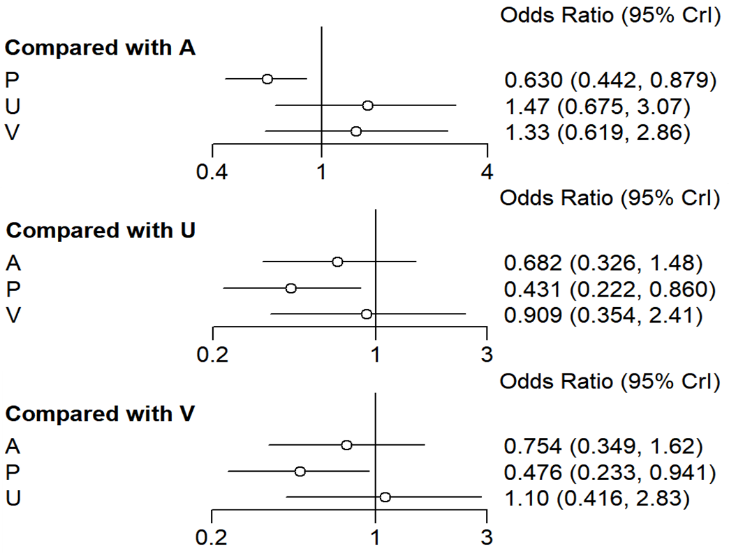** |
| **Rank Graph with Rank Probability** |  |
| **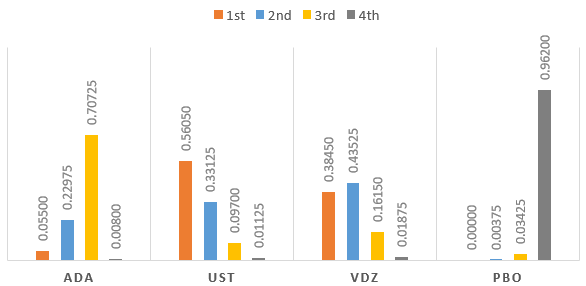** |  |
| **Endoscopic Improvement (Induction) – excluding Suzuki et al. 2014** | |
| **Network plot** | **Inconsistency** |
| **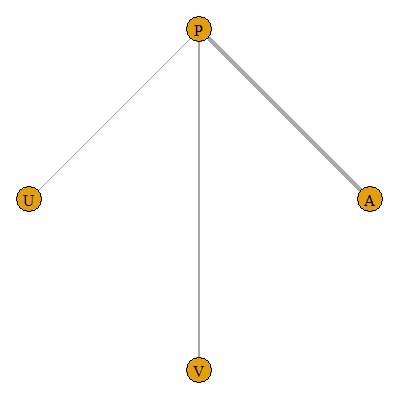** | **N/A** |
| **Forest plot (vs PBO)** | **Forest plot (ADA vs UST vs VDZ)** |
| **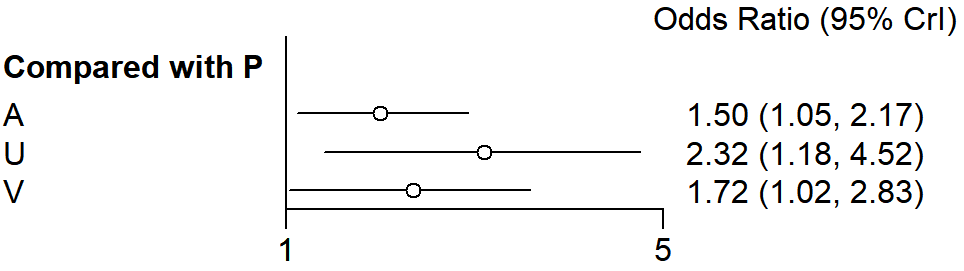** | **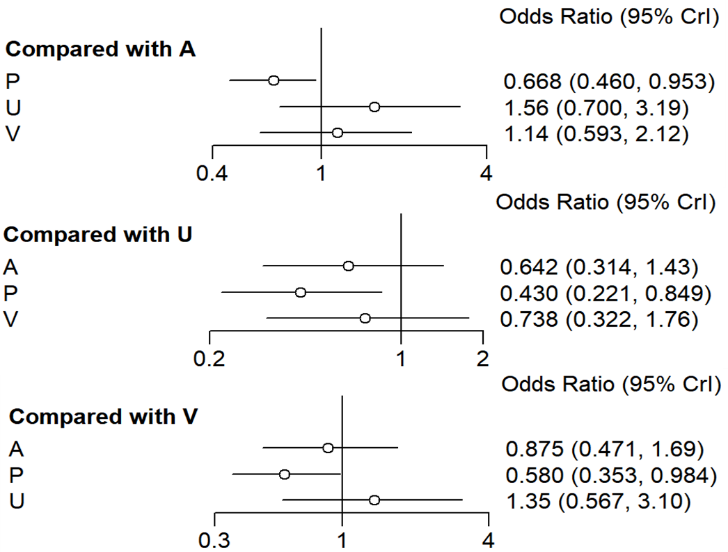** |
| **Rank Graph with Rank Probability** |  |
| **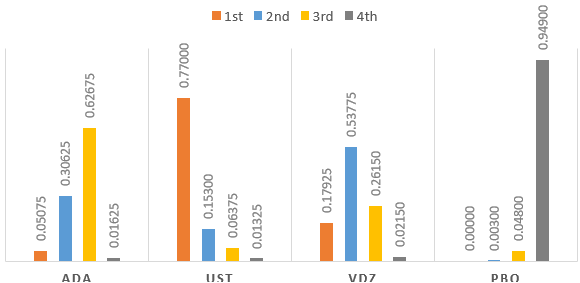** |  |
| **Endoscopic Improvement (Induction) – excluding Motoya et al. 2019 and Suzuki et al. 2014** | |
| **Network plot** | **Inconsistency** |
| **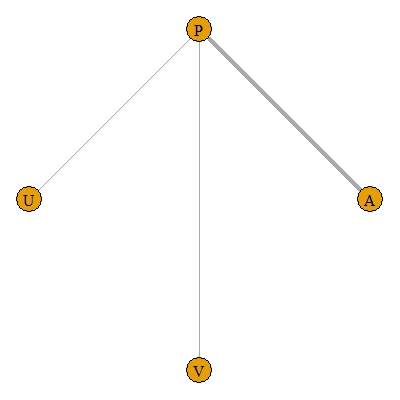** | **N/A** |
| **Forest plot (vs PBO)** | **Forest plot (ADA vs UST vs VDZ)** |
| **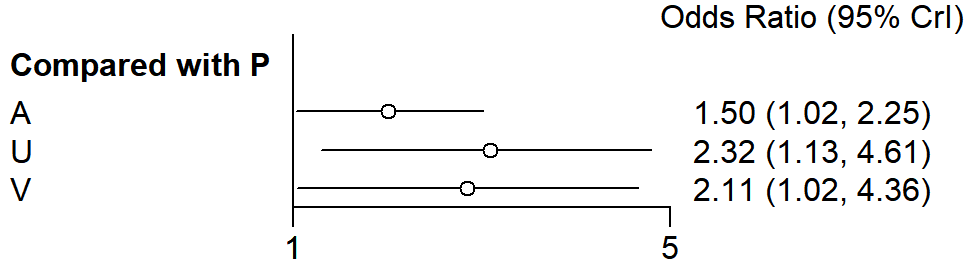** | **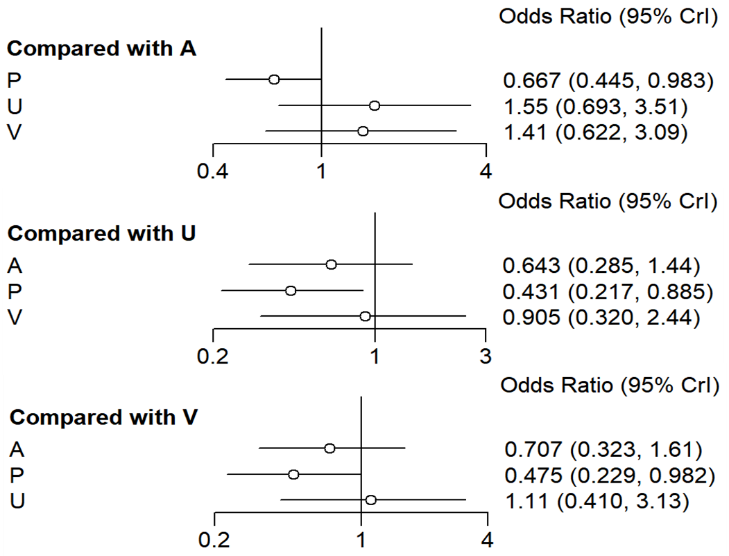** |
| **Rank Graph with Rank Probability** |  |
| **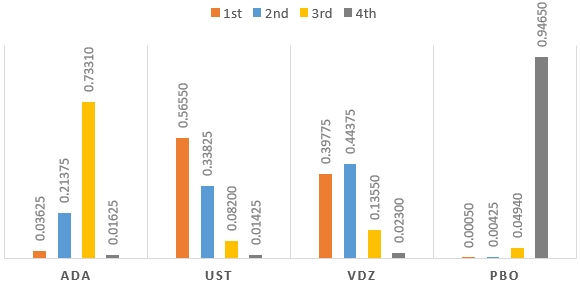** |  |
| **Endoscopic Improvement (Maintenance) – excluding Motoya et al. 2019** | |
| **Network plot** | **Inconsistency** |
| **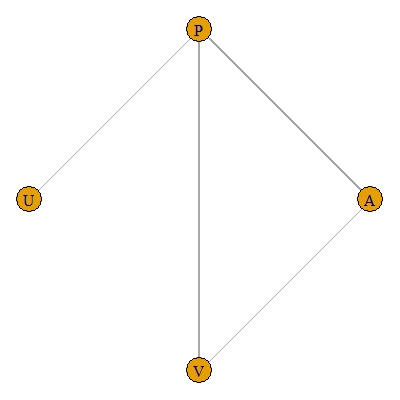** | **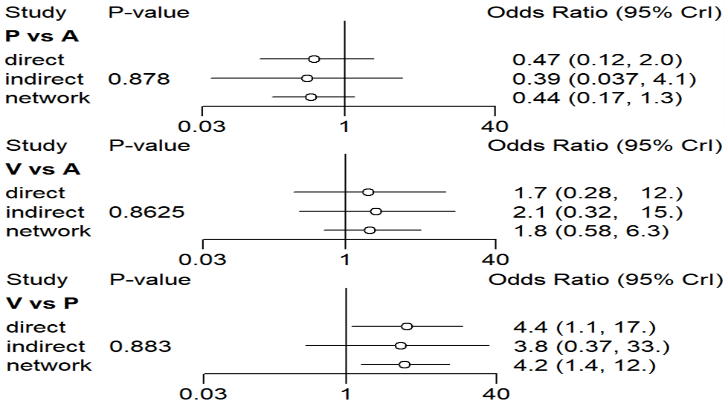** |
| **Forest plot (vs PBO)** | **Forest plot (ADA vs UST vs VDZ)** |
| **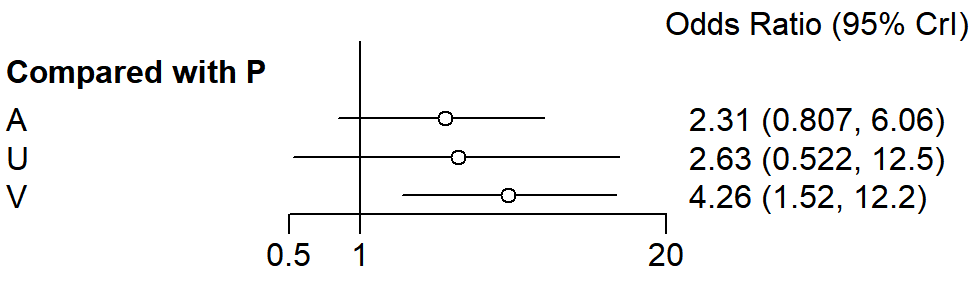** | **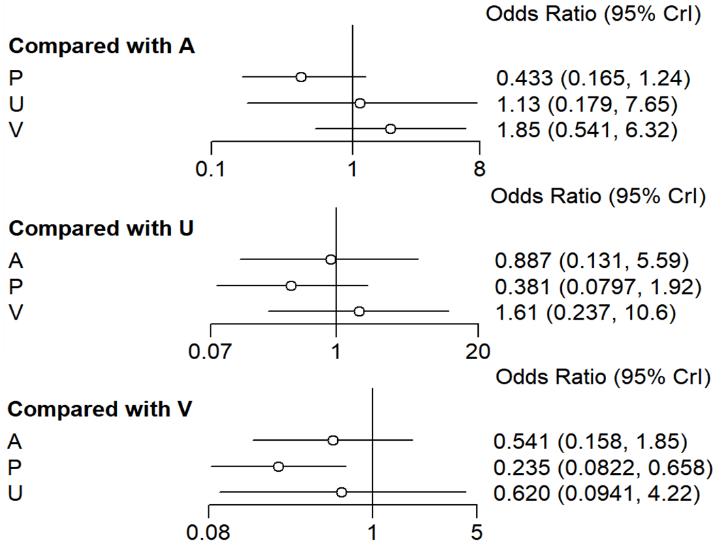** |
| **Rank Graph with Rank Probability** |  |
| **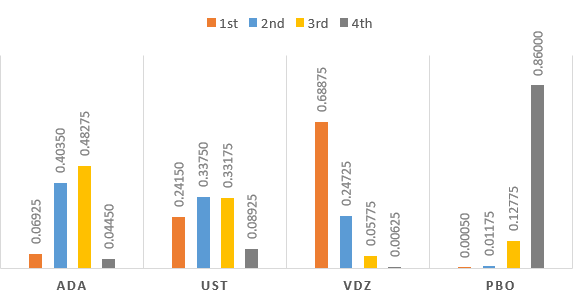** |  |
| **Endoscopic Improvement (Maintenance) – excluding Suzuki et al. 2014** | |
| **Network plot** | **Inconsistency** |
| **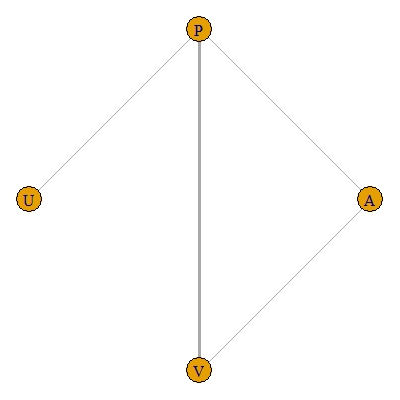** | **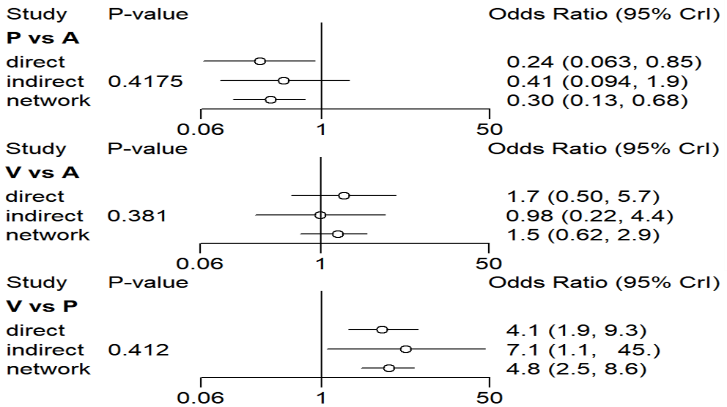** |
| **Forest plot (vs PBO)** | **Forest plot (ADA vs UST vs VDZ)** |
| **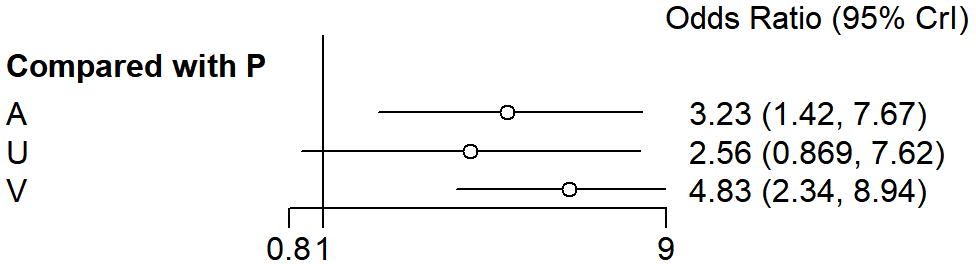** | **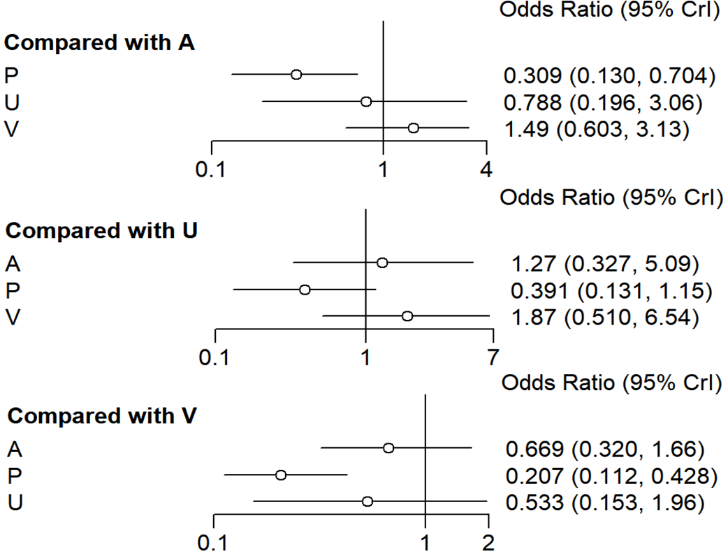** |
| **Rank Graph with Rank Probability** |  |
| **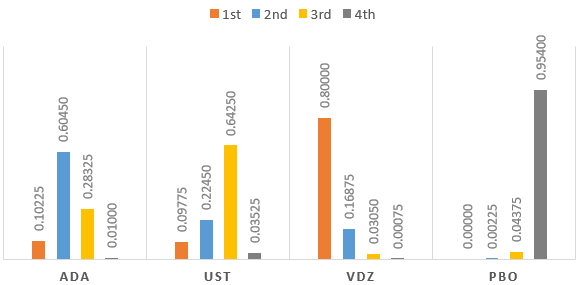** |  |
| **Endoscopic Improvement (Maintenance) – excluding Motoya et al. 2019 and Suzuki et al. 2014** | |
| **Network plot** | **Inconsistency** |
| **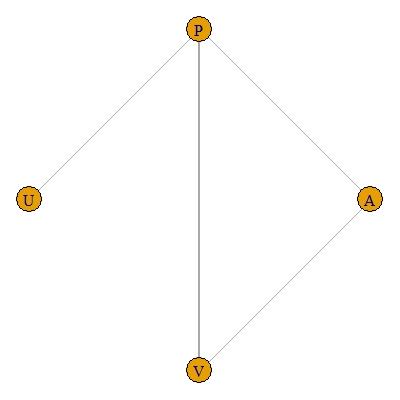** | **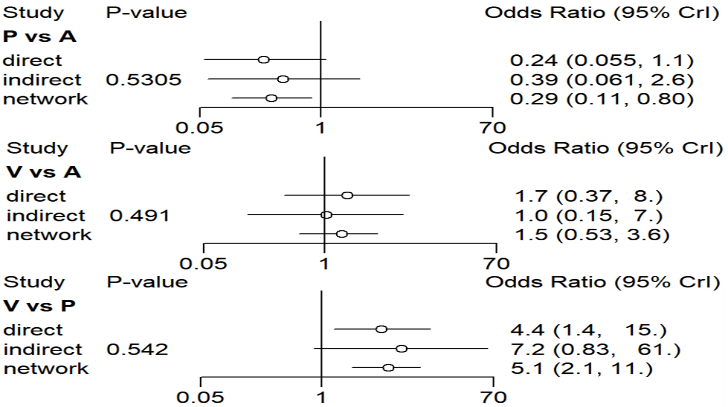** |
| **Forest plot (vs PBO)** | **Forest plot (ADA vs UST vs VDZ)** |
| **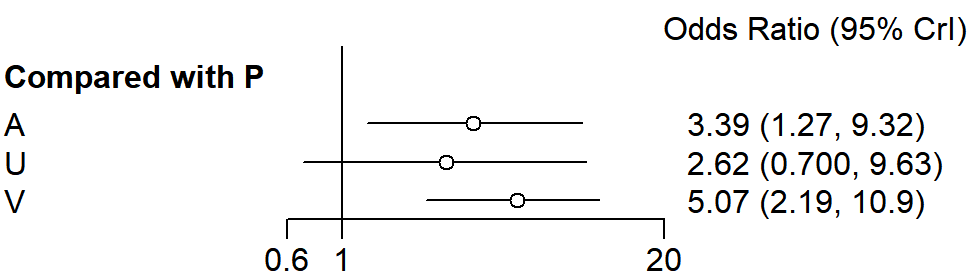** | **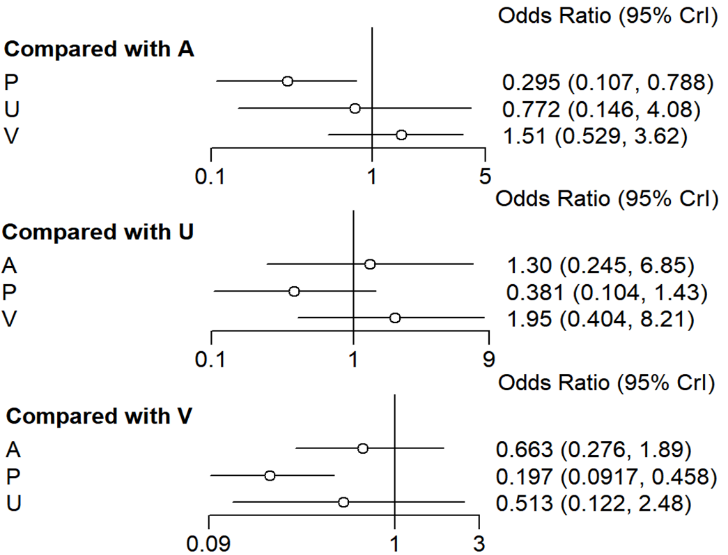** |
| **Rank Graph with Rank Probability** |  |
| **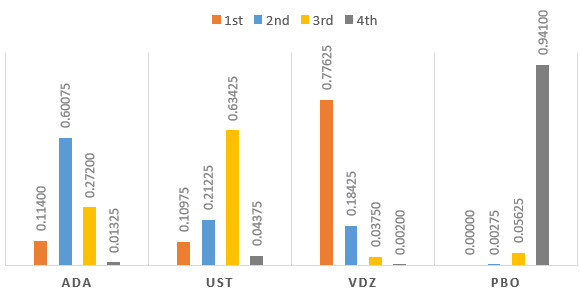** |  |
| **TEAE (Induction) – excluding Suzuki et al. 2014** | |
| **Network plot** | **Inconsistency** |
| **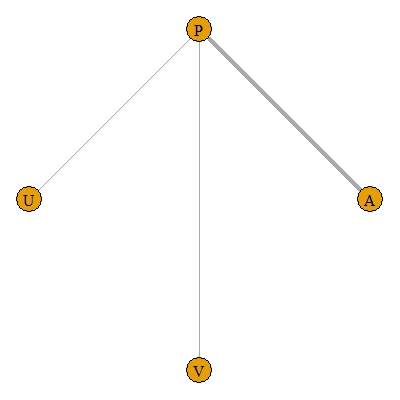** | **N/A** |
| **Forest plot (vs PBO)** | **Forest plot (ADA vs UST vs VDZ)** |
| **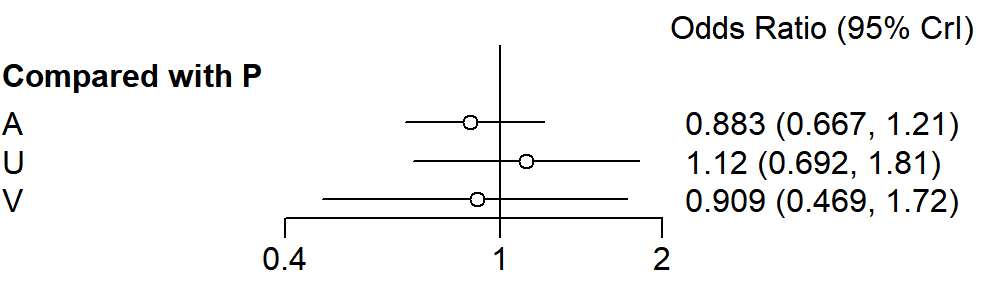** | **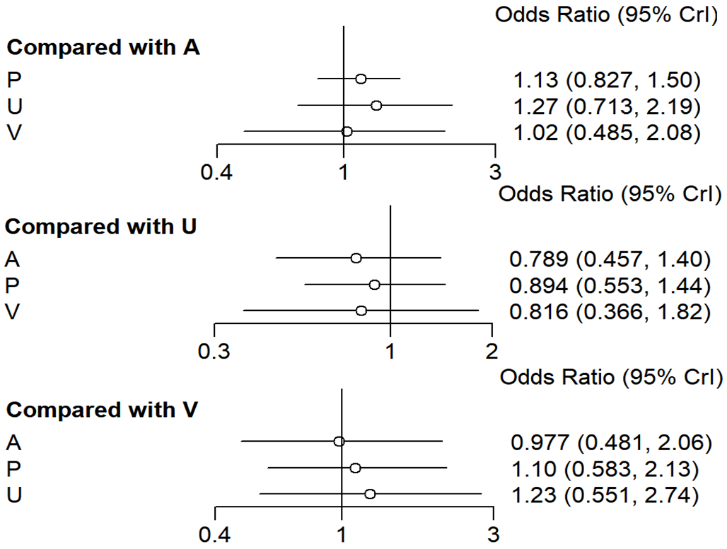** |
| **Rank Graph with Rank Probability** |  |
| **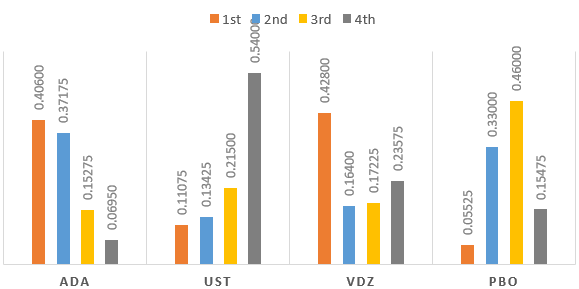** |  |
| **TEAE (Maintenance) – excluding Motoya et al. 2019** | |
| **Network plot** | **Inconsistency** |
| **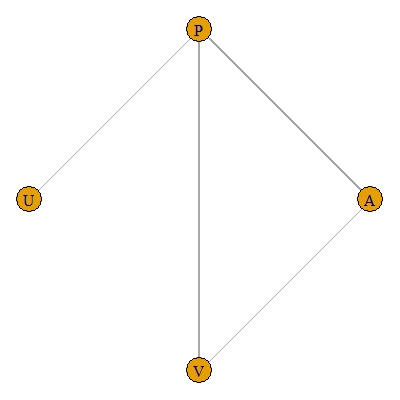** | **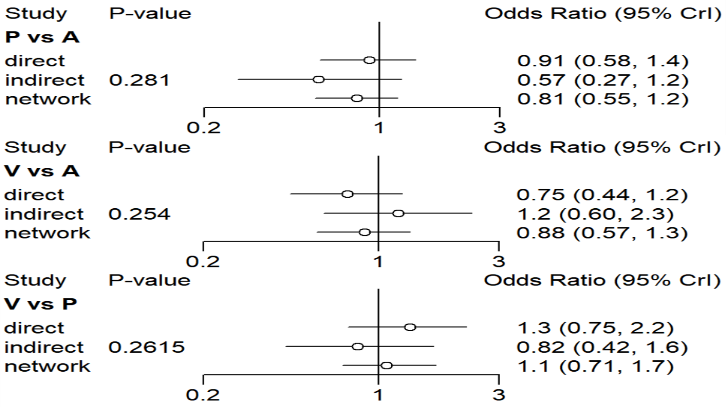** |
| **Forest plot (vs PBO)** | **Forest plot (ADA vs UST vs VDZ)** |
| **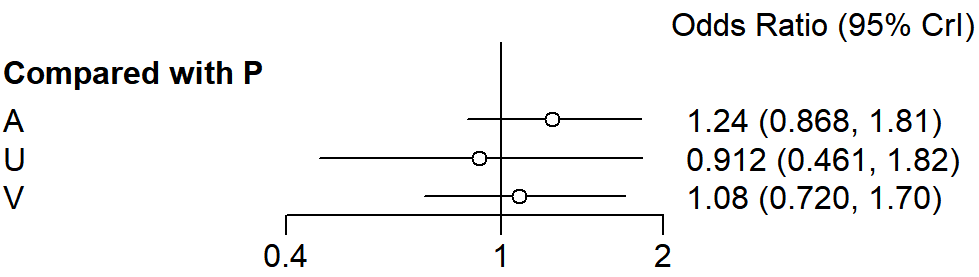** | **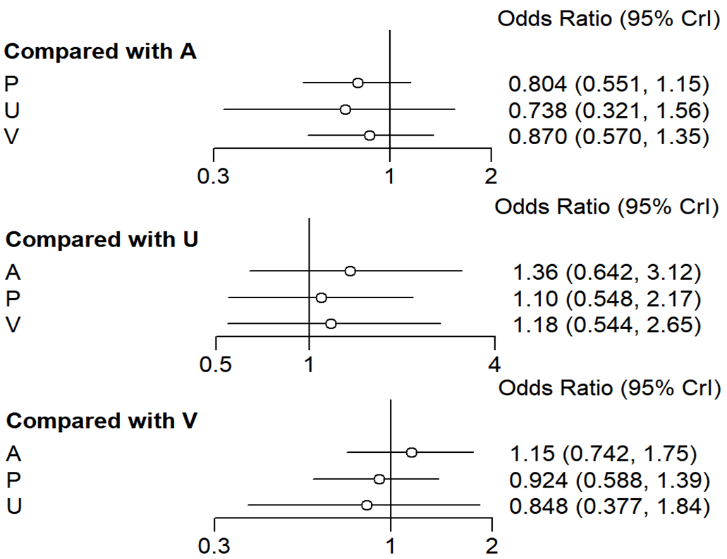** |
| **Rank Graph with Rank Probability** |  |
| **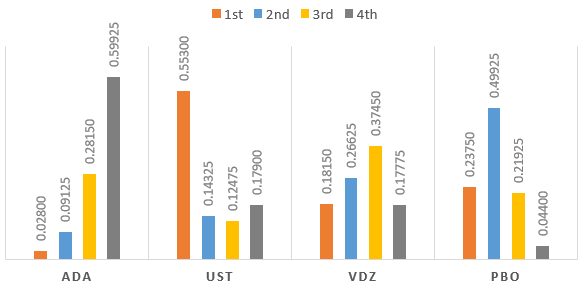** |  |
| **TEAE (Maintenance) – excluding Suzuki et al. 2014** | |
| **Network plot** | **Inconsistency** |
| **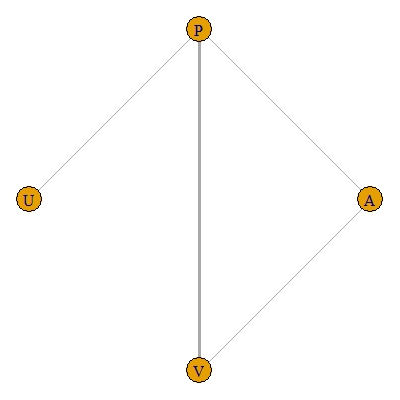** | **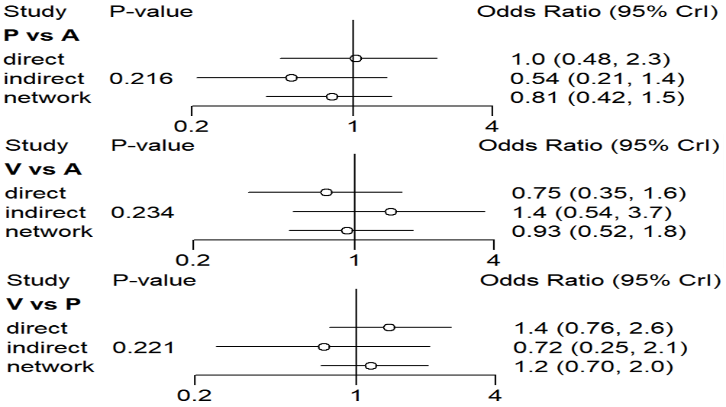** |
| **Forest plot (vs PBO)** | **Forest plot (ADA vs UST vs VDZ)** |
| **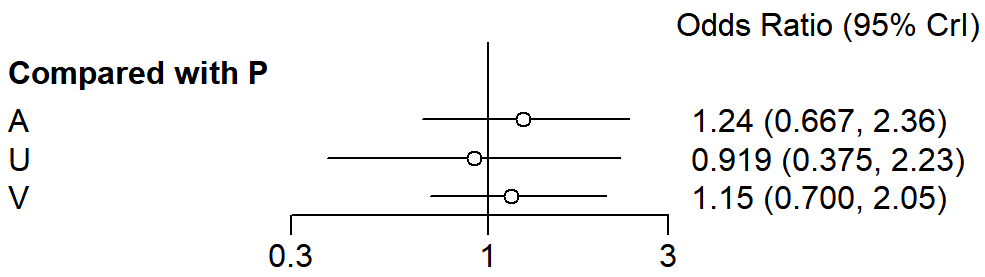** | **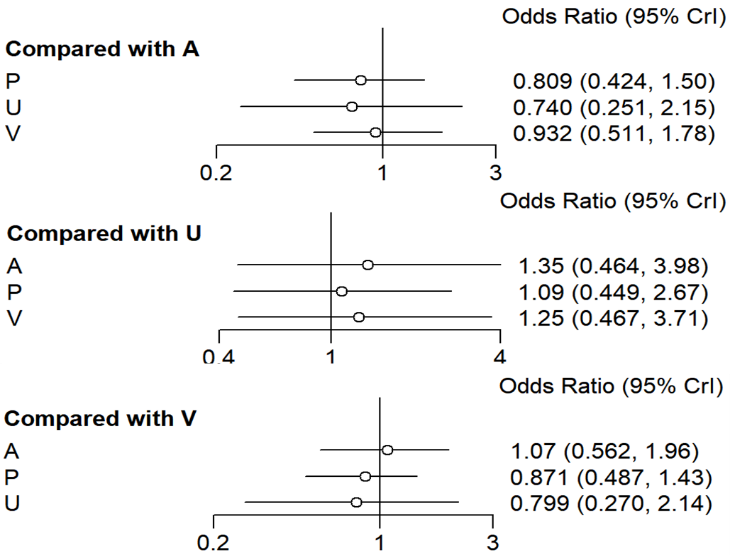** |
| **Rank Graph with Rank Probability** |  |
| **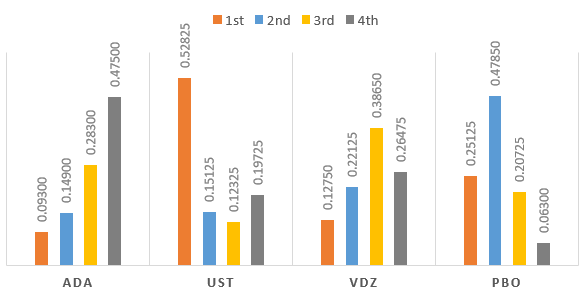** |  |
| **TEAE (Maintenance) – excluding Motoya et al. 2019 and Suzuki et al. 2014** | |
| **Network plot** | **Inconsistency** |
| **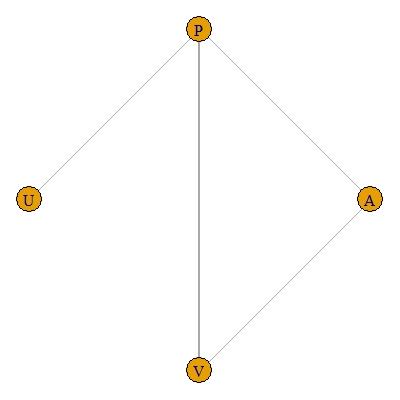** | **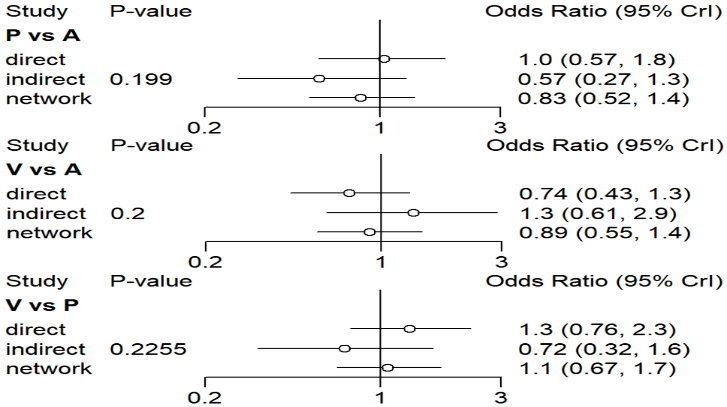** |
| **Forest plot (vs PBO)** | **Forest plot (ADA vs UST vs VDZ)** |
| **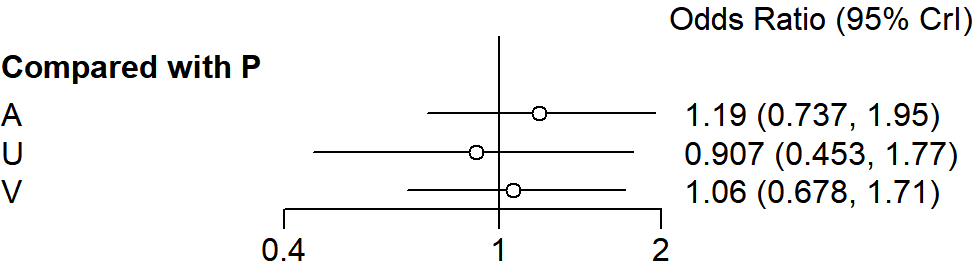** | **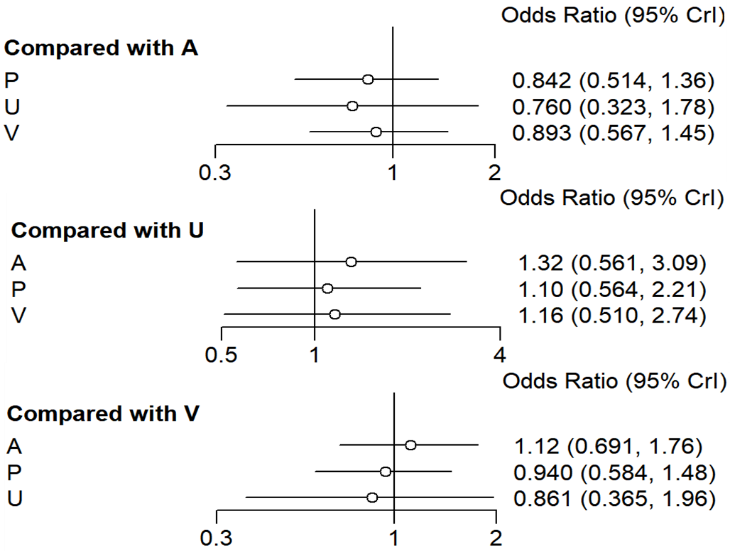** |
| **Rank Graph with Rank Probability** |  |
| **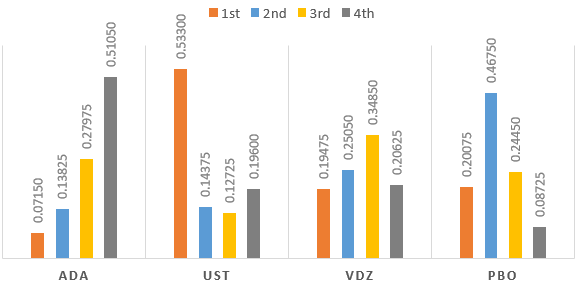** |  |
| **TESAE (Induction) – excluding Suzuki et al. 2014** | |
| **Network plot** | **Inconsistency** |
| **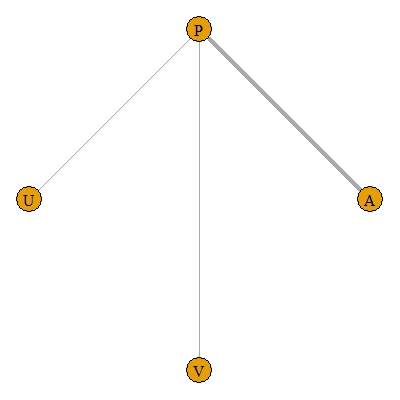** | **N/A** |
| **Forest plot (vs PBO)** | **Forest plot (ADA vs UST vs VDZ)** |
| **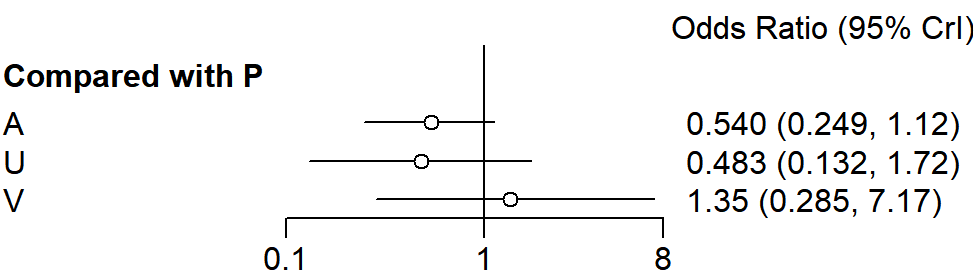** | **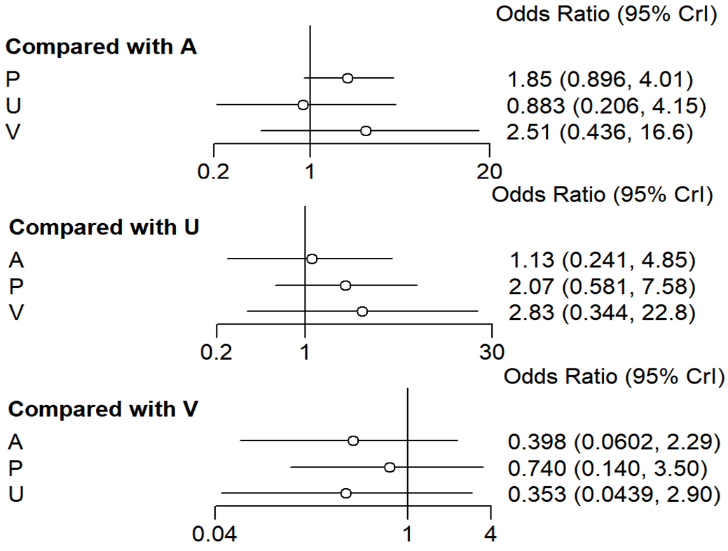** |
| **Rank Graph with Rank Probability** |  |
| **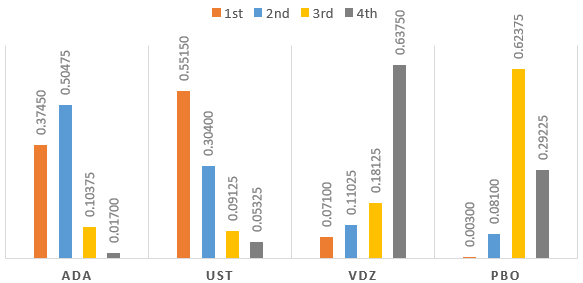** |  |
| **TESAE (Maintenance) – excluding Motoya et al. 2019** | |
| **Network plot** | **Inconsistency** |
| **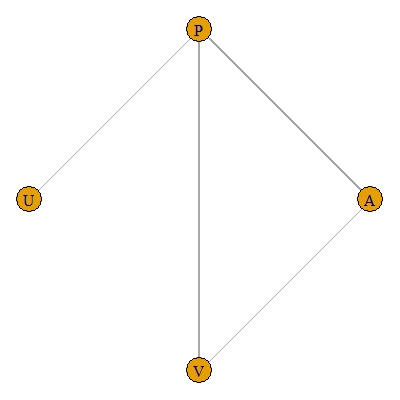** | **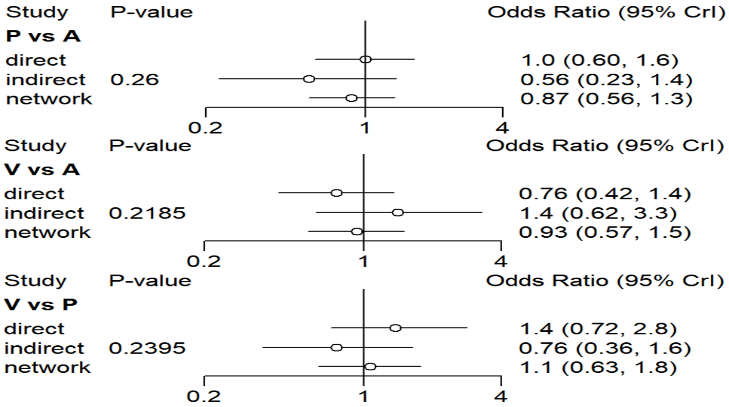** |
| **Forest plot (vs PBO)** | **Forest plot (ADA vs UST vs VDZ)** |
| **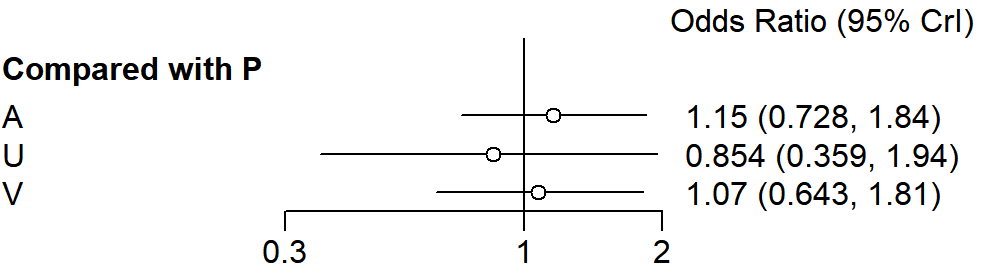** | **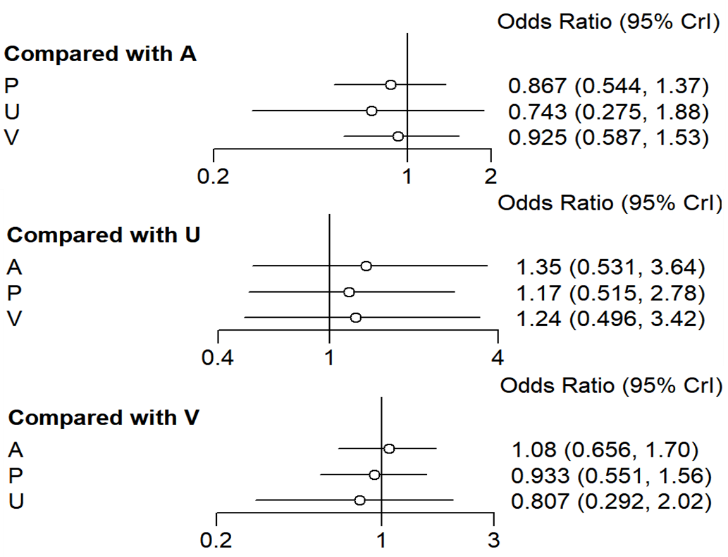** |
| **Rank Graph with Rank Probability** |  |
| **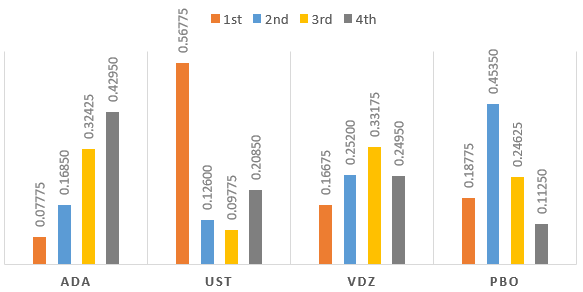** |  |
| **TESAE (Maintenance) – excluding Suzuki et al. 2014** | |
| **Network plot** | **Inconsistency** |
|  |  |
| **Forest plot (vs PBO)** | **Forest plot (ADA vs UST vs VDZ)** |
|  |  |
| **Rank Graph with Rank Probability** |  |
|  |  |
| **TESAE (Maintenance) – excluding Motoya et al. 2019 and Suzuki et al. 2014** | |
| **Network plot** | **Inconsistency** |
|  |  |
| **Forest plot (vs PBO)** | **Forest plot (ADA vs UST vs VDZ)** |
|  |  |
| **Rank Graph with Rank Probability** |  |
|  |  |

| **Infection (Induction) – excluding Suzuki et al. 2014** | |
| --- | --- |
| **Network plot** | **Inconsistency** |
|  | **N/A** |
| **Forest plot (vs PBO)** | **Forest plot (ADA vs UST vs VDZ)** |
|  |  |
| **Rank Graph with Rank Probability** |  |
|  |  |
| **Infection (Maintenance) – excluding Motoya et al. 2019** | |
| **Network plot** | **Inconsistency** |
|  |  |
| **Forest plot (vs PBO)** | **Forest plot (ADA vs UST vs VDZ)** |
|  |  |
| **Rank Graph with Rank Probability** |  |
|  |  |
| **Infection (Maintenance) – excluding Suzuki et al. 2014** | |
| **Network plot** | **Inconsistency** |
|  |  |
| **Forest plot (vs PBO)** | **Forest plot (ADA vs UST vs VDZ)** |
|  |  |
| **Rank Graph with Rank Probability** |  |
|  |  |
| **Infection (Maintenance) – excluding Motoya et al. 2019 and Suzuki et al. 2014** | |
| **Network plot** | **Inconsistency** |
|  |  |
| **Forest plot (vs PBO)** | **Forest plot (ADA vs UST vs VDZ)** |
|  |  |
| **Rank Graph with Rank Probability** |  |
|  |  |
